# Information Revolutions and Information Transitions: Counting, Sealing, Writing in Iran 10,000–300 BC

**DOI:** 10.1515/janeh-2024-0027

**Published:** 2025-04-23

**Authors:** Roger Matthews, Amy Richardson, Hassan Fazeli Nashli, Donna de Groene

**Affiliations:** Department of Archaeology, University of Reading, Reading, UK; Institute of Archaeology, University of Tehran, Tehran, Iran; Department of Archaeology, University College Cork, Cork, Ireland

**Keywords:** information, revolution, Iran, counting, writing, sealing

## Abstract

In this paper, we present a comprehensive analysis of counting, sealing, and writing practices in ancient Iran, spanning approximately 9000 years from the Neolithic to the Iron Age. The survival of clay (and occasionally stone or metal) media for administration in early Iran provides direct evidence for the development of bureaucratic practices. These materials reveal how such practices were situated within a broad range of socio-political, cultural, and environmental circumstances. Through systematic review and statistical analysis of the surviving material residues of Iranian bureaucracy, we identify distinctive deep-time diachronic trends and patterns. Our findings examine the ways in which Iranian societies exhibited a more hesitant and episodic engagement with sealing and writing compared to their Mesopotamian neighbours. We consider how these differences may be contingent on the inherent fragility of the agricultural systems that underpinned Iranian societies from the Neolithic onwards. This research underscores the interconnectedness of environmental factors, social organization, and technological development in ancient Iran. By understanding the interplay of these factors, we gain valuable insights into the formation and evolution of Iranian societies over millennia.

## 1 Introduction and Research Framework

The ancient past of Iran, *c*. 10,000–300 BC, provides special opportunities for investigating long-term perspectives on a wide range of issues of critical importance, as outlined in a recent pan-Iranian synthetic study (Matthews and Fazeli Nashli 2022). Such issues include human-environment interactions, including climate change (Sharifi et al. 2015; Kehl, Rafiei-Alavi, and Lahijani 2023), the development of social and genetic identities (Mehrjoo et al. 2019), including as related to gender and age (Daems 2018), and the origins, rise and collapse of complex social formations, including villages, urban centres, states, and empires (Meyer et al. 2019; Squitieri and Altaweel 2022). Within these broad contexts, the evidence from Iran for early administration and bureaucracy in the form of tokens, seals, seal impressions, sealings and inscribed objects of various types, made principally of clay, together constitute a unique deep-time assemblage of material culture that invites investigation through the application of synthetic and analytical approaches. The widespread use of clay, and occasionally stone or metal, as physical media for administration in early Iran means that we are blessed with a wealth of surviving material as direct evidence of what may broadly be termed ‘bureaucracy’. These materials have considerable potential to inform us on the practices of counting, sealing, and writing through periods from the Early Neolithic onwards, and on how such practices were situated within a broad range of socio-political, cultural, and environmental circumstances.

The material evidence for counting, sealing, and writing in Iran 10,000–300 BC has featured in a host of previous studies, as cited throughout this article, but never at the scale and scope attempted here. While researchers have focused, and continue to focus, on aspects of this wealth of bureaucratic evidence, usually within period-specific or material-specific brackets, there has not previously been a systematic study involving diachronic, synthetic, and holistic approaches, such as we undertake here. With regard to the Iranian context of the development of bureaucracy, a host of major issues might profitably be addressed. What were the special circumstances within which the many and varied manifestations of counting, sealing, and writing were developed by human actors from the Neolithic onwards? How can the study of the material residues of Iranian bureaucracy and the specifics of their archaeological and architectural contexts, inform us on the nature of Iran’s past societies and on the practices of counting, sealing, and writing within those societies? How best do we account for the major lacunae in the Iranian evidence, in particular for the practice of writing period by period and region and region? A recent study of sealing practices in Mesopotamia and in the Indus civilisation characterises the use of seals and sealings in Mesopotamia as providing “coercive institutions with a means of dressing up extractive debt to look like balanced reciprocity, creating a moral cover that allowed elites to begin collecting interest” (Green 2020: 7). The practice of seal use in the Indus civilisation, by contrast, is interpreted by Green as more associated with household activity and lacking in elite connection. How might the Iranian evidence fit within these two sharply contrasting schemes, if it fits at all?

The rich Iranian evidence allows us to examine such issues at the birth of Early Neolithic village societies of the region and at the origins of state-level societies in the Chalcolithic and Early Bronze Age, as well as within the context of more mature states and empires of the Iron Age. Arguably no region on earth enables a fuller and more detailed investigation of the deep-time trends and patterns in the practices of counting, sealing, and writing than the lands of early Iran.

Furthermore, the contexts and circumstances lacking in archaeological evidence for bureaucratic activity also draw our attention. Thus, despite being chronologically and geographically bracketed by numerate, literate, seal-using societies, the peoples of the so-called Early Transcaucasian Bronze Age world, which included regions of western and northern Iran, resolutely eschewed use of any form of bureaucracy involving the material attributes in clay so richly attested in other periods and places of Iran and its neighbours (Matthews and Fazeli Nashli 2022: 236–259). These and other indications that some societies may have chosen not to engage in writing, sealing or other forms of bureaucratic activity (Lamberg-Karlovsky 2003), even while previous and contemporary neighbouring societies fully indulged in such practices, hint at an ideological or cultural basis underpinning such society-wide practices and avoidances, in need of further articulation and investigation.

Iranian societies have never evolved in isolation from the worlds around them. The development of villages, urban centres, states, and empires across Iran over the millennia can be apprehended only through situation of the Iranian evidence within broad geographical and historical contexts, whether they lie in Mesopotamia and Anatolia to the west and northwest, the Caucasus to the north, Central Asia to the east and northeast, South Asia to the southeast, or the Persian Gulf shores and beyond to the south and southwest. Even the so-called Proto-Elamite phenomenon of the late fourth and early third millennia BC (Abdi 2003), whose specific archaeological remains are restricted to sites lying within the borders of modern Iran, has to be understood within the context of early state formation in Mesopotamia to the west in preceding and contemporary centuries. The study of Iran, ancient or modern, demands a transregional approach, as well as consideration of regional diversity within Iran.

All the material evidence featuring in our study certainly or plausibly relates to ancient practices of counting, sealing, or writing, or a combination of two or all three of the above. Our studied items take the form of material residues of early bureaucratic activity, focused on the need or desire to record information that by virtue of its physicality could be transmitted through time and space, as an enduring record. The act of recording information in such ways has to be situated within a matrix of social interactions, whereby specific pieces of information are agreed (or insisted) upon, materialised in some form, and referred to during interactions of varying types. Contextual and associational analysis of the material residues of counting, sealing, and writing therefore has the potential to inform us on the nature of the social interactions in the societies within which such practices took place (Bierschenk 2019; Boyes, Steele, and Astoreca 2021). While tokens, seals, sealings, and inscribed objects might serve a spectrum of social purposes, including symbolic, religious, and ideological (Ameri et al. 2018; Palka 2021; Bennison-Chapman 2023), in prehistoric and early historic Southwest Asia their greatest role was within the context of a vast range of material or virtual transactions, many of which, such as the recording of temple offerings, may also have borne cultic or religious connotations. In sum, we treat the corpora of material evidence for counting, sealing, and writing as access points for articulating and investigating administrative systems through deep time in Iran. These corpora represent, essentially, the ‘paperwork’ of ancient bureaucratic practices that underpinned administrative systems (Basello and Giovinazzo 2018), which in turn were situated within varying socio-political structures ranging from small villages to entire empires.

What might be the key features of these administrative systems? From previous studies of corpora of tokens, seals, sealings, and inscribed objects, we know that they are likely to be dominated by a meticulous concern to oversee and record the inflow, storage, and outflow of a wide range of resources, including animals, animal products, and agricultural produce, attesting in short “the farm as an accounting laboratory” (Giraudeau 2017). They may also relate to the feeding, housing, and work allocation of dependent or slave labour, and to the construction and maintenance of centrally organised projects ranging from canal digging to temple building. We might also expect them to be closely tied to festivals and cultic occasions whereby offerings were made to specific deities and carefully recorded as such. They will also constitute records of accountability whereby individuals are identified as responsible for overseeing specific actions or activities. Many inherent attributes of such systems may not feature at all in the content and form of the material evidence, as they will have been known and understood by all the system’s participants and therefore not needful of explicit articulation (Bierschenk and de Sardan 2019). Such implicit features might include hierarchies of administrative office(r)s, protocols for receiving, storing, and disbursing resources, chains of communication, and details of the physical spaces within which bureaucracies were operating. These significant ‘absences’ stress the potential value of archaeology in augmenting and contextualising the information obtainable directly from the material residues of counting, sealing, and writing. As we will see throughout the article, the ability of archaeological evidence to help us in this regard is highly variable through the space and time of ancient Iran.

## 2 Methods

**Figure 1:**
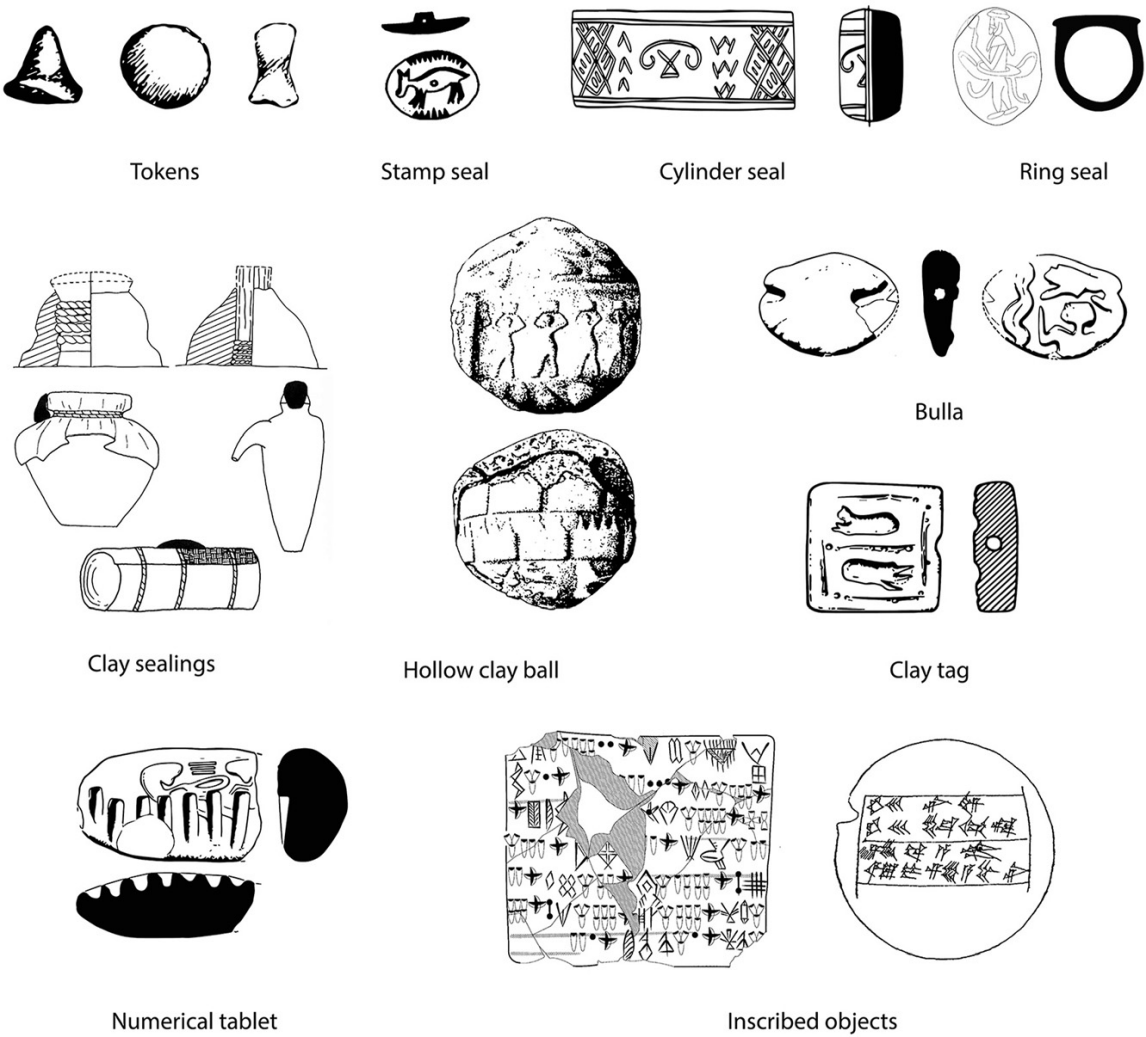
Types of CSWO featured in this study (tokens after Fazeli Nashli and Moghimi 2013; stamp seal after Alizadeh 2008: fig. 76; cylinder seal after Abedi 2016: fig. 15; ring seal after Hasanpur, Hashemi, and Overlaet 2015: pl. 6; clay sealing after Matthews and Richardson 2018: fig. 12; hollow clay ball after Delougaz, Kantor, and Alizadeh 1996: pl. 133; bulla and clay tag after Le Brun 2021: fig. 17; numerical tablet after Le Brun 2021: fig. 16; inscribed objects after Mutin 2013: fig. 5.1 and Malayeri 2013: fig. 5).

We start with the definition of key terms used throughout the article. By **token**, we refer to deliberately modelled shapes, which appear from the Early Neolithic onwards, in a range of forms, including cones, spheres, cylinders, disks, and bicones (Figure 1). Tokens are most commonly made of clay, often fire-hardened, but may also be made of stone. Wooden tokens may have been used but have not been recovered from archaeological sites. While tokens are first attested in the Early Neolithic period in Iran and across the Middle East, from *c*. 8000 BC (Richardson 2019; Palka 2021; Bennison-Chapman 2023), they continue in sporadic use throughout the periods featuring in this study. From *c*. 4000 BC, so-called ‘complex tokens’, distinguished by more elaborate shapes and by incisions or other markings added to the basic forms, are found at a limited range of major Iranian sites almost exclusively situated in the Susiana region of Khuzestan, in step with comparable developments at the early urban site of Uruk across the border in Iraq (Schmandt-Besserat 2018: 372).

It is possible that our restriction here of the term token to define a range of geometric shapes is too narrow, and that other modelled clay and stone shapes such as figurines of vessels, animals, and humans also fulfilled roles within early systems of counting and recording, but because of the many suggested connections between geometric token forms and early instances of counting and writing on clay tablets from Iran and Mesopotamia (Schmandt-Besserat 1996; Valério and Ferrara 2022; see also discussion in Englund 1993; Zimansky 1993), we are here focusing on those forms of token that can more directly be associated with bureaucratic activities of some kind.

By **seal**, we refer to two basic types. The **stamp seal** (Figure 1) is attested in Iran from at least *c*. 6000 BC, with the earliest excavated example from the Neolithic site of Khaleseh (Valipour et al. 2013). As with tokens, stamp seals continue in episodic use across the millennia of this study’s timespan. The **cylinder seal** (Figure 1) is attested over Iran and Mesopotamia from *c*. 3500 BC and continues in use throughout the study period, yielding a wealth of information through its rich figurative and geometric iconographies as well as through its uses across a wide range of bureaucratic activity (Pittman 2013). **Signet or ring seals** are found exclusively in the Achaemenid period, within the chronological remit of this article.

By **clay sealing** (Figure 1), we refer to those pieces of clay that were originally affixed to a solid item, which could range from a door closure device to a pot-mouth covering, or from the tied neck of a sack to the knotted wrapping of a split-reed basket or bale. Clay sealings typically, but by no means always, bear impression(s) of one or more stamp seals and/or cylinder seals on their outer, obverse surface(s). On their reverse faces, they frequently bear impressions of the objects or items to which they had been affixed, yielding negative images of profiles of wooden or stone door pegs, plastered surfaces of walls or door jambs, profiles of ceramic vessel rims, necks, and shoulders covered in leather or fine sacking, or knots in string wound to secure container closures, for example (Figure 1). Functional analysis of assemblages of clay sealings, often found in large quantities from discrete archaeological contexts such as rubbish dumps, can shed light on administrative practices including the relative frequencies of storeroom (door) sealing actions, likely local to the site where recovered, as compared to container sealings, affixed to portable items that may have originated from outside the site where archaeologically recovered, for example (Matthews and Richardson 2018). Seals and sealings were thus employed within an integrated system of recording of and accounting for the receipt, storage, and distribution of a range of commodities. One component of that system seems to have been the use of clay test strips or identity tags, bearing a single clear impression of a stamp or cylinder seal as a referent for the identification of sealed goods or as ‘calling cards’ of individual identity. Such pieces are attested as early as the fourth millennium BC in Middle Uruk deposits at Sharafabad in Susiana (Wright, Miller, and Redding 1980: 277), and in many later assemblages.

**Hollow clay balls** (also called ‘envelopes’ by Schmandt-Besserat 2018: 374) (Figure 1) form a type of sealed clay object with a limited geographical and chronological distribution, but widely interpreted as a critical developmental stage across the fields of counting, sealing, and writing (Dittmann 2012; Sauer and Sürenhagen 2016; Schmandt-Besserat 2018: 374–377; Charvát 2019). Hollow clay balls bear impressions of cylinder and/or stamp seals across their outer surfaces in some cases, especially at Susa, accompanied by impressed marks that, at least occasionally, correlate with groups of geometric shaped clay tokens contained in the hollow interior space of the sealed clay ball (Woods 2010). Following Delougaz, Kantor, and Alizadeh (1996, 119–120), we use the term **bulla** (pl. bullae) to refer exclusively to ovoid or facetted clay pieces that have almost always been shaped around a length of string which would have been attached to something else. Bullae have also been classed as labels or ‘fusiform tags’ (Pittman 2013: 321), and as ‘solid ovoid cretulae’ in the exemplary publication of the Arslantepe sealings (Fiandra and Frangipane 2007: 19). Common use of the term bullae to refer to hollow clay balls (Bennison-Chapman 2023: 215) is misleading and deprives the terminology of a significant term to describe a special and distinctive type of clay object.

A further important and distinctive group of objects are so-called ‘**clay tags**’, taking the form of small rectangular or pillow-shaped pieces. Such objects are found largely in the Late Chalcolithic period and are characterised by being deliberately baked – uniquely amongst clay bureaucratic objects from this and many other periods – by being pierced longitudinally, with evidence for attachment by string to some type of container, and by displaying a small number of proto-cuneiform signs (Schmandt-Besserat 1992a: 218–219; Szarzynska 1994). While the majority of clay tags come from Uruk IV–III levels at Uruk (Englund 1998: 57), at least two examples come from unknown contexts at Susa, each with a single simple proto-cuneiform sign (Szarzynska 1994: 7).

**Numerical tablets** (Figure 1) are solid, generally rectangular pieces of clay bearing number signs impressed with a stylus, and often displaying seal impressions. They are broadly found across the Late Chalcolithic, later fourth millennium BC worlds of Mesopotamia and western Iran (Englund 1998: 50–51). Finally, by **inscribed object** (Figure 1) we mean an item bearing an inscription of any kind. The earliest inscribed objects from Iran include so-called numero-ideographic tablets, which share features with numerical tablets but with the addition of 1–2 ideographic signs representing discrete objects such as herded animals or dairy products (Englund 1998: 51–56). Contemporary or slightly later in date are Proto-Elamite tablets, found so far at eight sites of late fourth-early third millennia BC date across Iran, but with a heavy emphasis on Susa as the major provenance (Dahl 2019). Later forms of inscribed objects include clay tablets in Akkadian or Elamite scripts, principally found in western and southwestern Iran, plus clay bricks, stone statues, stelae, and seals, items of jewellery, and any other form of object upon which script has been inscribed. In the text and figure captions that follow, we use the abbreviation ‘CSWO’ (counting, sealing, writing object) as a catch-all term for any of the above categories of object.

With this set of key definitions in mind, and drawing initially on the extensive bibliography compiled for Matthews and Fazeli Nashli (2022), we began by producing a summary Excel spreadsheet with data arranged chronologically and by material type. This spreadsheet was used as a platform for a more systematic and exhaustive compilation of relevant data in an Access database. Upon completion, the database comprises a total of almost 45,000 material objects from a total of 99 archaeological sites distributed across Iran through the period 10,000–300 BC. The charts, maps, and figures presented in this article have all been generated using data from this source. Online Supplementary Data 1 presents all CSWO data collated for this project.

We faced several challenges in compiling data for this study, similar to those encountered in a bold synthesis of the entirety of the cuneiform textual corpus and its spatial and temporal distribution (Rattenborg et al. 2021, 2023). Extremely few of the 99 archaeological sites featured in our database have been published to an extent that allows confidence in the completeness and accuracy of the available evidence. We have often had to ‘guesstimate’ the precise quantities of types of objects, where definitive numbers are not available, and have frequently found information on archaeological contexts and associated materials to be lacking or inadequate. We have also had to guard against double counting of objects, a particular danger when dealing with major sites such as Susa, where publication of relevant items, such as seals and sealings, has been distributed across a diverse range of sources published by many authors over many decades, often with limited or no quantitative or contextual information. Nevertheless, we are confident that despite these possible shortcomings, our data are resilient at least at the large scale and robust enough for demonstrating and investigating major trends in bureaucratic activity through space and time across the past of Iran.

A further concern is the issue of how representative the surviving material evidence is of the original bureaucratic assemblages themselves. While we are indeed blessed with a wealth of evidence in the form of the predominantly clay items treated here, we are aware that significant use was made of organic materials including parchment and papyrus as vehicles for documents such as those written in Aramaic during the Achaemenid period (Black 2008), none of which survive from Iran. There is also the possibility that wood and other organic materials may have been used within bureaucratic systems in all periods of Iran’s early past. While we can do little more than acknowledge this potential taphonomic concern, we note that from the surviving available evidence we have no indication of the original presence of parallel systems of bureaucratic activities relying on organic materials that have failed to survive. As regards the evidence from the later fourth millennium BC onwards, the predominant use of the cuneiform script through application of a stylus into leather-hard clay, combined with the persistent use of the stamp and cylinder seal rolled over clay, all suggest a bureaucratic system dominated by the use of clay as its principal vehicle.

In the following sections we present and discuss the CSWO evidence from Iran period by period, region by region, and site by site. We end each period section with a summary discussion of key points relating to that period, before concluding the article with a broader interrogation of the evidence across the deep-time past of Iran, and an articulation of possible future directions in CSWO research as it relates both to the past of Iran and more broadly across the discipline.

## 3 Neolithic Numeracy, 10,000–5200 BC

A significant information transition attends the sedentarisation of human communities at the dawn of the agricultural age throughout the Neolithic period, an episode of fundamental change in the human condition and in the nature of relationships between humans and all aspects of their environments, including climate, material resources, plants, and animals (Matthews and Fazeli Nashli 2013). Increasing quantities and regularisation of tokens, focused on spheres, cylinders, discs, and cones, at least suggest a concern to model clay into significant and recurrent forms that persist across time and space in the Neolithic of Iran and adjacent regions. As highlighted by Palka (2021) and Bennison-Chapman (2019, 2023) and discussed below, tokens served a diverse range of functions within Neolithic communities, possibly including monitoring of agricultural produce, use in games or other social activities, and as tokens of identity, emotion, or feelings when deposited in human graves, for example. If we include the evidence from the Transitional Chalcolithic site of Zagheh in this information revolution, we can also associate the systematic use of tokens both with structured counting systems and with activities involved in early craft production such as pottery manufacture. All these attributes can be viewed within the context of the sedentarisation of human communities, with its increased opportunities for social engagement within and between early farming and herding societies of Iran, as well as for the accumulation of resources by individuals or groups of individuals who might be concerned to keep account of those resources by some material means.

Material evidence for the development of numeracy begins in the Neolithic of Iran, a period of transformative change (Matthews and Fazeli Nashli 2013, 2022: 54–110). Between 10,000 and 5200 BC, significant environmental changes set the stage for shifts in human behaviour. An amelioration in climate from the start of the Holocene, *c*. 9750 BC, coincided with the earliest evidence for the use of clay tokens in the Zagros. The warmer, wetter conditions had an impact on habitable areas, promoting the increase in grass species and drought resistant *Pistacia* (Bosomworth, Fleitmann, and Rabbani 2020). This changing environment facilitated early sedentism and management of plants and animals, encouraging new approaches to social structures and the curation of resources.

At Early Neolithic sites, small clay geometric objects were shaped and lightly baked in their hundreds, in the form of balls, cones, cylinders, and discs. These humble objects have been connected with later developments in numeracy and literacy, drawing parallels with the tokens enfolded in bullae in the Chalcolithic, and similarities in the symbols used in early cuneiform documents (Schmandt-Besserat 2010). There remains debate over whether these tokens can be considered as numerical devices prior to the late seventh millennium BC, and the simple geometric forms may have had multiple uses that were variable and contextually dependent (Bennison-Chapman 2019). Numerosity, abstract conceptions, and material scaffolding for counting have been identified in human activity from the Upper Palaeolithic onwards, most often in association with time-marking (De Smedt and De Cruz 2011; Overmann 2013; Bacon et al. 2023). Certainly, a well-established material practice for counting was in place by 6000 BC, and it is highly likely that clay tokens played a significant role in its development. Clay tokens may have been used as mnemonic devices by individuals from the Early Neolithic onwards, spread between communities throughout Iran and beyond, carrying with them incipient numeracy.

**Figure 2:**
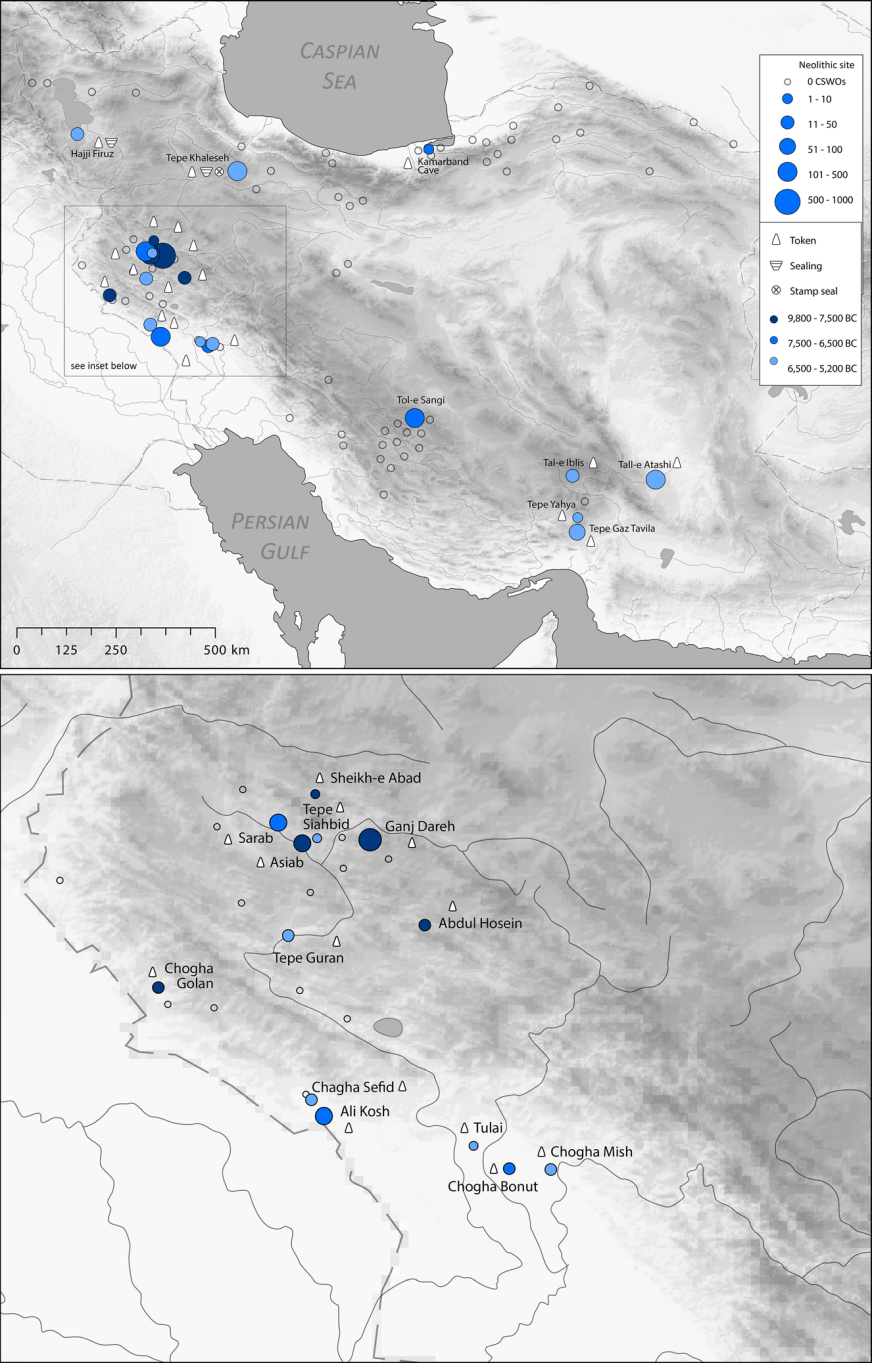
Map of CSWOs from Neolithic sites of Iran.

**Figure 3:**
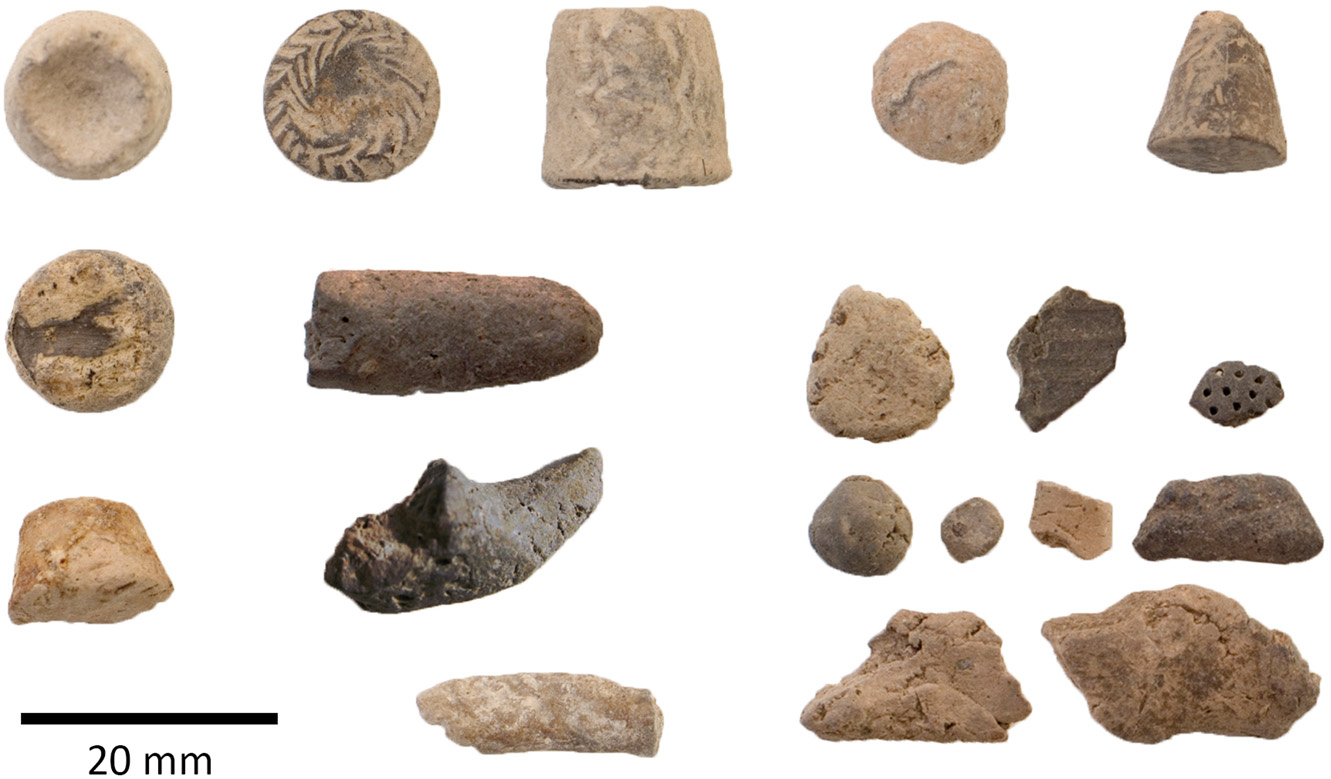
Clay tokens from Sheikh-e Abad.

**Figure 4:**
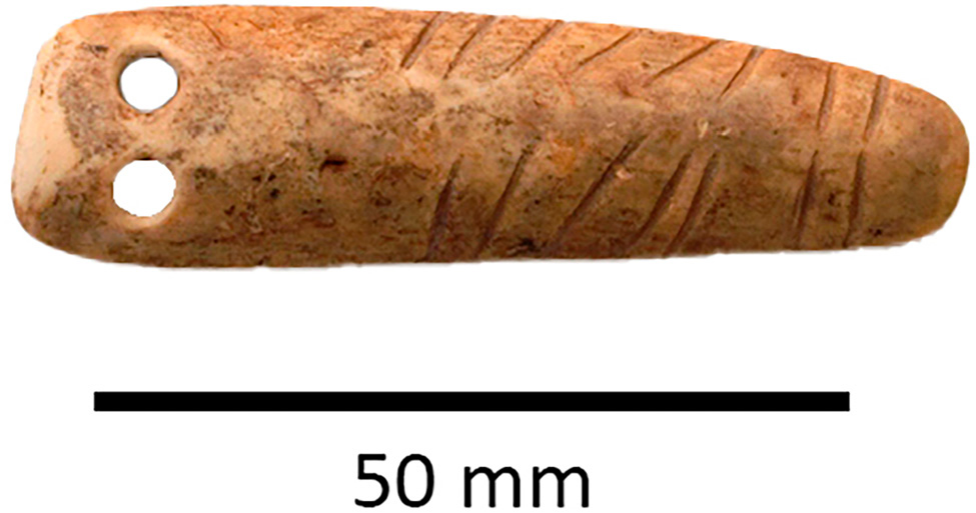
Incised bone pendant from Sheikh-e Abad.

The earliest use of tokens in Iran is heavily concentrated in the Central Zagros, first seen at Asiab in the mid-tenth millennium BC (Figure 2). Almost 200 clay tokens have been recovered from excavations, the majority of which are clay balls and cylinders, the remainder in the form of cones, discs, or ovoids (Schmandt-Besserat 1992b: 103–110). Richter’s re-excavation and expansion of Braidwood’s interventions has recovered tokens from within the fill of a building (Richter et al. 2021). In the ninth millennium BC tokens were also in use at nearby Sheikh-e Abad (Cole, Matthews, and Richardson 2013). The clay tokens recovered from the site of Sheikh-e Abad represent an early phase in the use of tokens at Neolithic sites (Figure 3). Situated in the high Zagros, the site is located close to contemporary settlements at Ganj Dareh, Asiab, and Abdul Hosein (Matthews et al. 2013). The Early Neolithic community at Sheikh-e Abad was on the cusp of early domestication, with evidence for the management and penning of animals. The earliest token present, a simple baked-clay ball shape comes from the early ninth millennium BC, a handful of small clay shapes in the early eighth millennium BC, followed by repetitive use of conical and trapezoidal tokens by the mid-eighth millennium BC, most of which were recovered from external areas (Cole, Matthews, and Richardson 2013). Also notable from Sheikh-e Abad is a bone pendant with incised lines in two distinct groups along both edges of the exterior bone surface (Figure 4). This object is highly polished from handling and may have served as a tally or time reckoner of some sort (Cole, Matthews, and Richardson 2013: fig. 11.4).

At Ganj Dareh, where the largest assemblage has been collected from late ninth and early eighth millennium BC deposits, 672 tokens were recovered, almost all of which were cones (Broman Morales and Smith 1990; Riel-Salvatore, Lythe, and Albornoz 2021). From the Central Zagros heartland, the Early Neolithic use of tokens spread southeast to the community at Abdul Hosein (Pullar 1990) and southwest to Chogha Golan (Riehl et al. 2015), formed into a range of shapes focused on cones, balls, and cylinders. In the Early Neolithic, tokens were distributed across both external and internal deposits, with emphasis on the former (Riel-Salvatore, Lythe, and Albornoz 2021). However, two tokens were recovered from burials at Abdul Hosein, indicative of a variety of purpose and significance amongst the diverse communities (Pullar 1990).

From the late eighth millennium BC, clay was used more extensively for a variety of purposes, particularly notable at the site of Sarab. Excavations recovered hundreds of figurines and shaped clay fragments, including around 300 balls, discs, cones, and tetrahedrons (Broman Morales 1990). In addition, 540 fragments of concavo-convex baked clay objects were identified, with the appearance of having been wrapped around a stick, alongside two fragments of clay with basket impressions, three with matting, and three with fabric impressions (Alizadeh 2003). The evidence suggests clay was deliberately applied to surfaces and could be considered an early form of sealing, although without seal impressions. Many of the clay objects at Sarab were retrieved from ashy external deposits (McDonald 1979). To the south, clay tokens in a variety of geometric shapes from late eighth millennium BC Chogha Bonut, in Susiana, were also recovered from fire installations (Alizadeh 2003). The frequency with which clay tokens have been recovered in proximity to fire may be a result of preservation conditions, but it may also be considered that clay devices were, for the most part, casually disposed of in external working areas once they had served their purpose. Clay tokens were a short-lived and disposable resource, easily remade and rarely baked hard for longevity in the Early Neolithic. It is worth noting that in the Early Neolithic, clay tokens were not cautiously stored or cached inside for record-keeping and their function may be predominantly associated with external, short-lived activities.

On the Deh Luran Plain, a substantial volume of clay tokens was recovered from external, ashy deposits at late eighth to early seventh millennium BC Ali Kosh. Of the 494 clay tokens recorded, 465 took the form of cylinders (Hole, Flannery, and Neely 1969). Twenty ball-shaped tokens from Ali Kosh, similar to those of upland Early Neolithic sites, have been interpreted as evidence for the development of a Neolithic “farming redistribution economy”, in part due to the concurrence with the use of non-native wheat species at Ali Kosh and the later use of the visually similar circular symbol for a measure of grain in early writing (Schmandt-Besserat 2018). A total of 34 tokens in three broad types and eight sub-types were also recovered from Late Neolithic levels at Chagha Sefid (Schmandt-Besserat 2018: 370; Hole 1977: 237). Further south, recent excavations at Tol-e Sangi have recovered 63 clay tokens, dating to the late eighth and early seventh millennium, and a possible clay sealing (Khanipour pers. comm.; Khanipour, Kordshooli, and Karami 2021).

The popular use of clay balls and cones continued on the Susiana Plain in the mid- to late seventh millennium BC at Chogha Mish (Alizadeh 2008; Delougaz, Kantor, and Alizadeh 1996) and Tulai (Hole 1974). At the latter site, clay geometric objects were accompanied by stone and bitumen balls (Schmandt-Besserat 1992b), demonstrating an experimentation with materials that were less readily available and had potentially greater permanence. Clay continued to be the preferred medium for the production of tokens throughout the sixth millennium BC, but the forms into which it was shaped demonstrated increasing complexity. In the Central Zagros, at Tepe Guran, 32 simple clay balls, cones, and discs were accompanied by two complex tokens with punctated or incised decoration across the surface (Schmandt-Besserat 1992b).

Along with the expanding repertoire in the sixth millennium BC, the use of clay tokens also spread further geographically. To the northeast, clay cone and cylinder tokens have been recovered from excavations at Kamarband Cave on the Caspian shore (Coon 1951). In northwest Iran, to the south of Lake Urmia, clay tokens were recovered from the human burials below the floors of mudbrick buildings at early sixth millennium BC Hajji Firuz (Voigt 1983). Voigt hypothesised that the figurines at Hajji Firuz were ‘vehicles of magic’ and the same may be suggested of the clay tokens, which were most often found in association with human burials. A particular concentration in a one-roomed structure included a cluster of five clay tokens over a deposit of human bone, tokens sealed between two floors, and also placed in a pit possibly as ritual caches. Elsewhere at the site, an ossuary also contained a cone-shaped token. Interpretations have suggested these tokens may have been regarded as talismans or played a role in healing magic, an interpretation echoed in Palka’s (2021) analysis of clay tokens in two burials at Tepe Guran. The evidence from Hajji Firuz supports the argument that tokens were multi-purpose tools (Bennison-Chapman 2019), not specifically created to administer agricultural produce, but rather that their use could be contextual, at times used for counting, or playing games, or kept as talismans, and that the objects, materials, and concepts that they signified could be myriad and changing.

Aside from tokens, Hajji Firuz yielded 14 clay sealings without seal impressions (Voigt 1983). These show signs of having been pressed against reeds, flat surfaces, pots, angular and cylindrical objects. Similar clay objects were observed at the earlier sites of Ganj Dareh and Sarab (Broman Morales 1990), but the clay sealings from Hajji Firuz mark the shift in the early sixth millennium BC towards the increased application of clay to seal objects prior to the use of stamp and cylinder seals. This application of clay has also been found at Tepe Khaleseh, in Zanjan Province, where hundreds of tokens were excavated in conjunction with two clay sealings bearing reed and fabric impressions (Valipour et al. 2013). Although the clay used to seal objects did not bear the traces of seal impressions, a single fired-clay stamp seal was recovered from the site. The archaeological evidence from Khaleseh has indicated wide-ranging activities, including the management of domesticated mammals, a pottery kiln, and the processing and storage of crops (Whitlam, Valipour, and Charles 2020).

By the early sixth millennium BC, the use of tokens had also extended along the southeastern corridor, to Tal-e Iblis (Caldwell 1967), Tall-e Atashi (Garazhian and Shakooie 2013) and Tepe Gaz Tavila (Schmandt-Besserat 1992b), at the latter site including incised and punctated complex tokens. At the site of Tepe Yahya in the Soghun valley, the ceramic Neolithic occupation of level VII dates to the late sixth and early fifth millennium BC. The mudbrick buildings include storage areas and there is evidence for use of copper in production of small artefacts such as pins and tacks (Thornton et al. 2002). Clay tokens in the form of cones and spheres are common at Tepe Yahya, with 64 in total and a further 20 in stone (Thornton et al. 2002). The assemblage at Tepe Yahya demonstrates increasing formalisation of the token repertoire. Two episodes are present in the Yahya token assemblage, the first in Neolithic level VII and the second in Chalcolithic level V (Beale 1986: 257–258; see below), at which time there was a shift towards deliberate firing of the clay tokens, giving greater durability and suggesting that they were in circulation for longer periods of time.

### 3.1 Summary of the Neolithic CSWO Evidence: More than Counters

**Figure 5:**
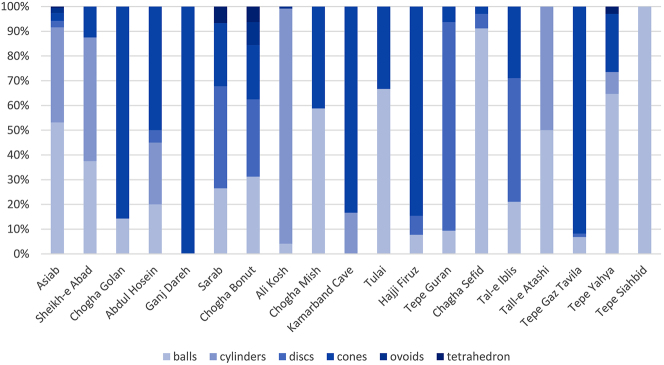
Proportions of most frequently recurring token shapes from Neolithic sites of Iran, organised chronologically.

Widespread use of simple clay tokens at a significant proportion of Neolithic sites, as attested at Sheikh-e Abad, Ganj Dareh, Ali Kosh, Tepe Yahya and other sites, may be associated with an increasing concern to record and control the agricultural economy, enjoyment of games involving counters, or their use in ritual or commemorative circumstances, such as those interred with human remains. The make-up of the token assemblages demonstrates significant variability in the proportions of different geometric shapes, indicating that these were used in highly localised systems of practice and perhaps across a wide range of activities (Figure 5). What is notable, however, is the longevity of particular forms, such as balls, cones, discs, and cylinders and their increasing regularity in form and quantity in the Late Neolithic, implying a developing formalisation of purpose. Over the course of the Neolithic, clay tokens appear to have been increasingly widely recognised, and accepted, as an abstract representation of something else, whether that be a numerical, social or material value.

Anthropological studies have clearly underlined numeracy and cultural complexity as intrinsically interlinked (McLaughlin and Schlaudt 2023; Overmann 2013, 2019, 2024), with each facilitating the other in the development of new ways of living and thinking. The management of resources demanded by the advent of agricultural practices would have increased pressure on communities to develop resilience strategies, such as the storage of surplus, creating volumes to be quantified. At Late Neolithic sites, the values conveyed by clay tokens may have been contextually specific, but the concept of the material representation of numbers was widespread and established a system that would remain in place for more than 5000 years, from the advent of agriculture until the invention of writing and arguably well beyond.

## 4 An Information Revolution: Chalcolithic Complexity, 5200–3200 BC

**Figure 6:**
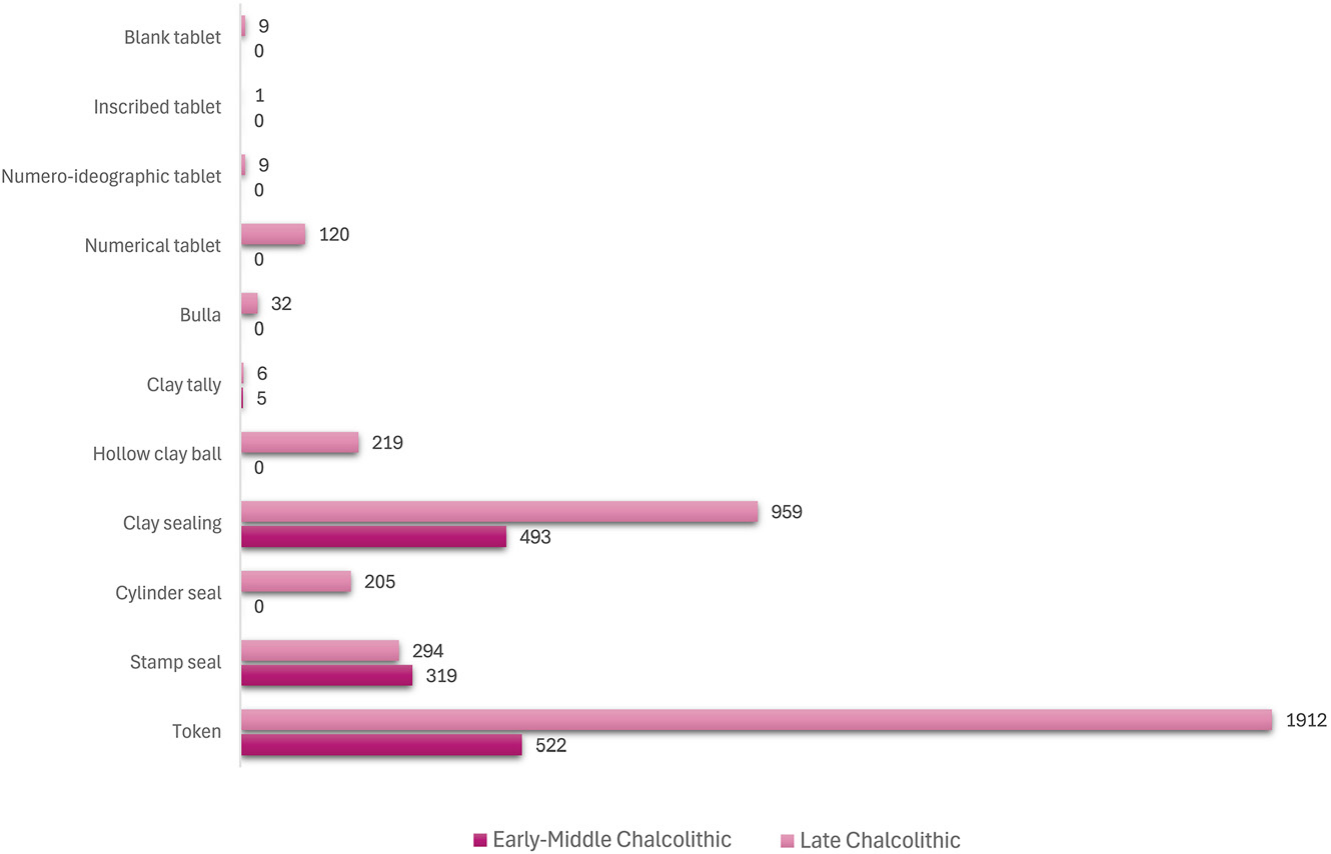
Chart to show CSWO use through the Chalcolithic period.

A second major transition, culminating in a true information revolution, can be traced through the centuries of the Chalcolithic period in Iran, with its heartbeat firmly in the major sites of Susa and Chogha Mish in Khuzestan through the Late Susa II period. A significant component of this transition is an intensification of token use through the fourth millennium BC, culminating at *c*. 3200 BC (Figure 6), succeeded by a rapid decline of their use into the Early Bronze Age, although not a complete disappearance. Major new components from the mid-fourth millennium BC onwards include the cylinder seal, enabling greater complexity in iconographic scale and scope, the hollow clay ball as both a container for tokens and a vehicle for lavish display of cylinder seal impressions, and solid clay tablets initially bearing solely numerical signs plus initial experiments with ideographic signs. While at Susa or Chogha Mish we lack evidence for the development of writing on clay tablets in the full Uruk IV–III proto-cuneiform tradition attested to the west in Lower Mesopotamia, at Susa in the late fourth millennium BC we do see the development of the Proto-Elamite writing system at least contemporary with Uruk III. We return to Proto-Elamite in the following section.

Notwithstanding a stratigraphic and cultural break between the Late Susa II and Proto-Elamite bureaucratic systems at Susa, the end-point of this information revolution at *c*. 3000 BC is the same at Susa as at Uruk (while differing in many specifics, of course), namely the use of a sophisticated and complex system of recording information through the impression or incision of styli on soft clay tablets, many of them sealed with cylinder seal impressions, which can reasonably be characterised as writing. Taken in concert with the graphic iconography of the seals and seal impressions on sealings and tablets, these artefacts point to a centralisation and institutionalisation of decision-making and administrative oversight as critical attributes of state-level societies (Wright 2013; Benati 2018). As articulated by Wright (1998, 175–176) “the polities that existed on the Susiana Plain are identified as states, albeit of an elemental form, because there is evidence of the operation of control hierarchies with contrastive specialization in the control process – specifically, specialization between aggregation of goods, information summary, information transport, information checking, adjudication, and probably policy making … able to integrate hundreds of communities and tens of thousands of people”. The evidence adduced by Wright in this argument takes the form of the clay bureaucratic objects in particular from Susa and Chogha Mish that are fully treated throughout this section.

**Figure 7:**
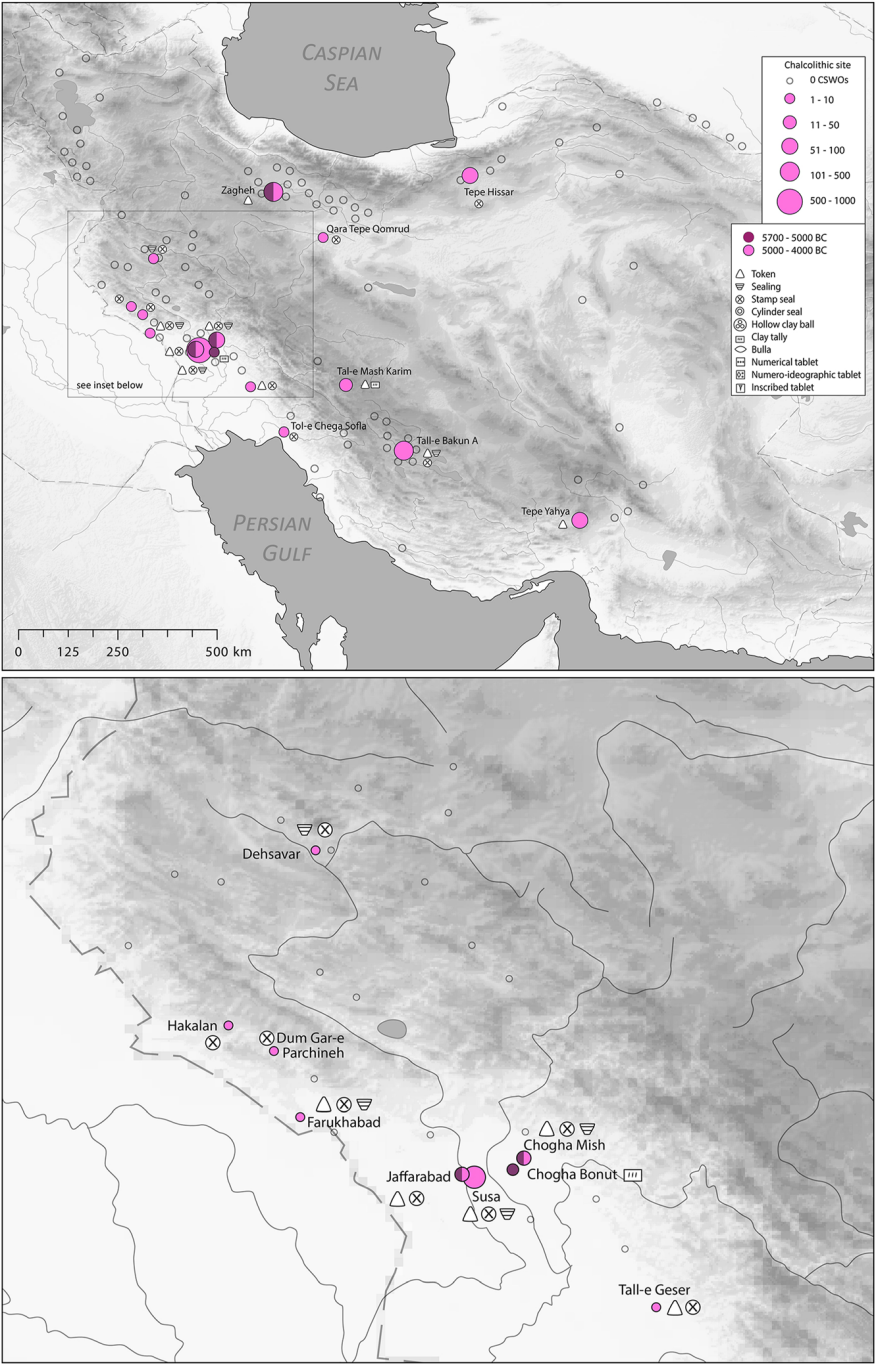
Map of CSWOs from Early-Middle Chalcolithic sites of Iran.

There is a clear step-change in the volume and complexity of evidence for bureaucratic activity through the course of the Chalcolithic period in Iran, which we can collectively characterise as signifying an information revolution. The maps (Figures 7 and 12) make clear the fundamental role played by the Khuzestan region of southwestern Iran through all stages of this revolution, while ongoing research continues to clarify the significance of developments in other regions of Iran, especially the north-central plains and foothills (Vidale, Fazeli Nashli, and Desset 2018). Let us examine all the Chalcolithic evidence in detail before drawing out some broader conclusions.

**Figure 8:**
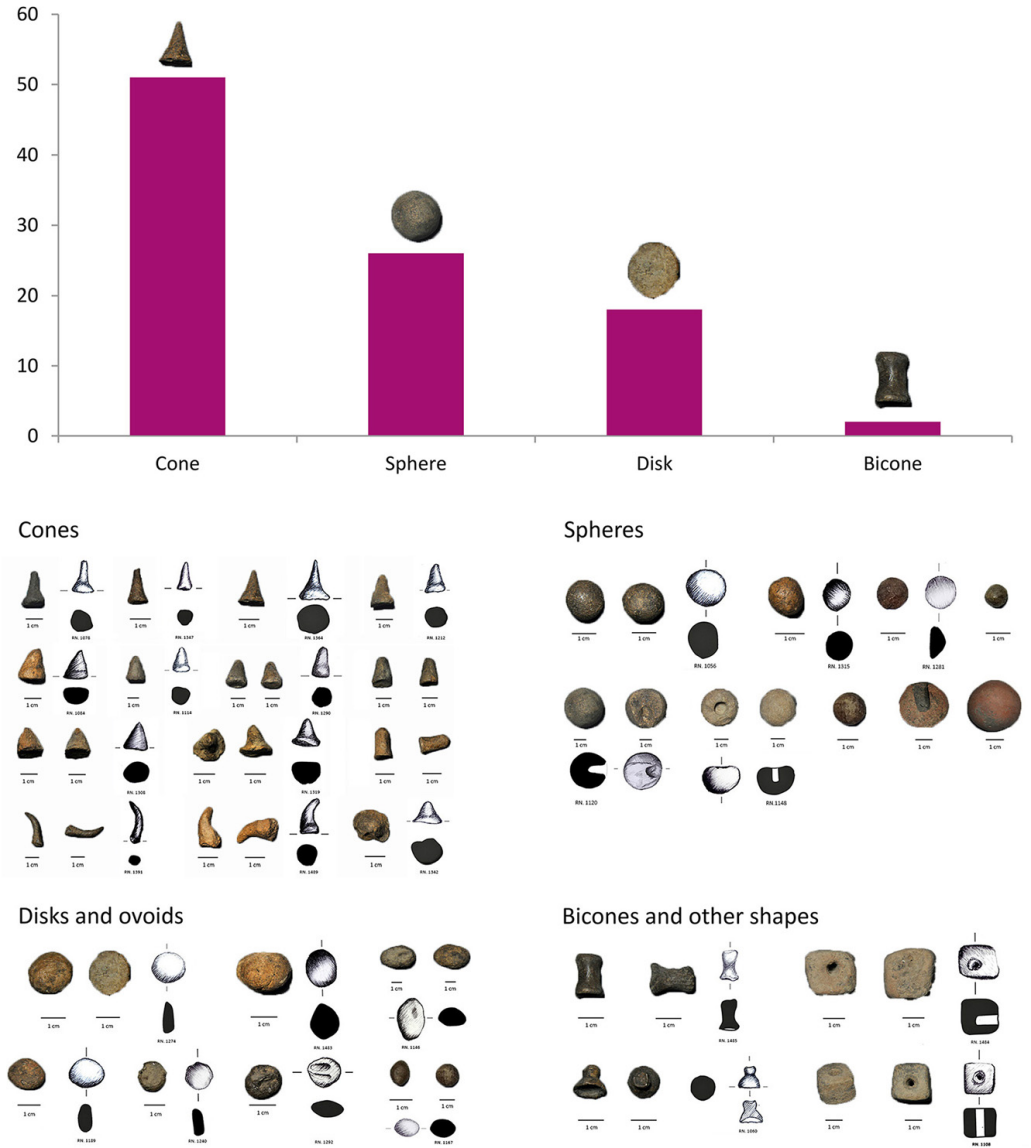
Clay tokens from the transitional Chalcolithic site of Zagheh (data and images from Fazeli Nashli and Moghimi 2013; Moghimi and Fazeli Nashli 2015).

In northern Iran, at the Transitional Chalcolithic site of Zagheh, 231 clay tokens in a range of four-five major forms may attest the control over movement of materials and commodities associated with craft production (Figure 8) (Fazeli Nashli and Moghimi 2013; Moghimi and Fazeli Nashli 2015). The sign-count of the four major forms of token – cone, sphere, disk, bicone – support an interpretation of this assemblage, found together in Trench N30 at Zagheh, as representing a basic cumulative-additive numerical system according to Chrisomalis’ (2010, 230–231) characterisation of numerical notation systems, at *c.* 5000 BC arguably the earliest convincing evidence for such a system.

**Figure 9:**
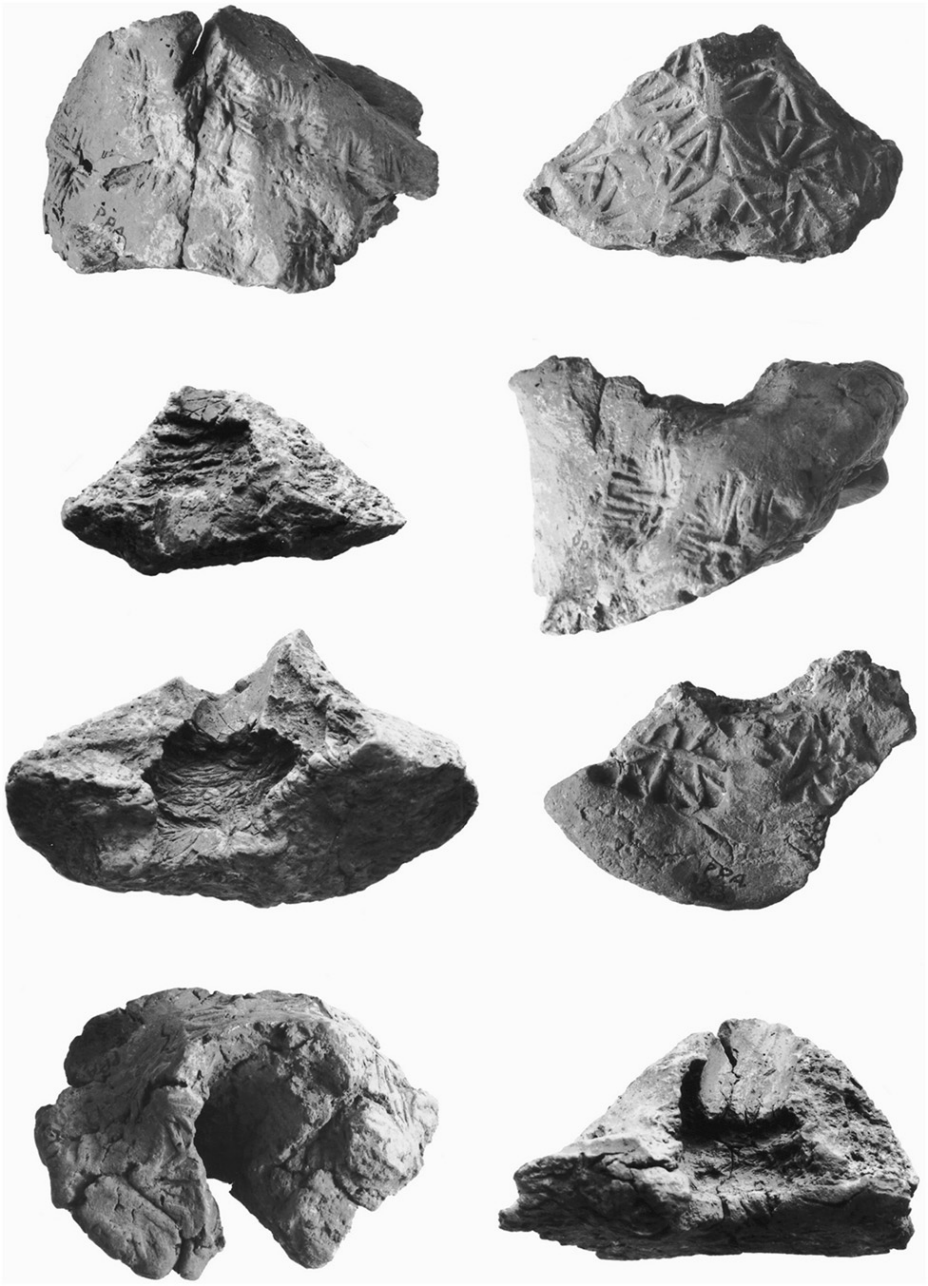
Clay sealings from Tall-e Bakun A (Alizadeh 2006: pl. 17; image courtesy of the Institute for the Study of Ancient Cultures of the University of Chicago).

By the Middle Chalcolithic, the use of tokens and stamp seals was widespread across much of Iran. But the largest numbers of items and the most complex assemblages are concentrated in three regions: north-central Iran, Fars, and Khuzestan. Outside these regions there is evidence for limited use of tokens only. In south-eastern Iran, the early adoption of token use attested in the Late Neolithic (Figure 5) is significantly reduced in the Chalcolithic, with 69 geometric tokens recovered solely from the site of Tepe Yahya, from contexts of Middle and Late Chalcolithic date (Beale 1986: 257–258). By contrast, in the Fars region, where tokens are not attested in the Neolithic period (with the probable exception of Tol-e Sangi as discussed above), we see a significant increase in bureaucratic activity evidenced by tokens, sealings, and stamp seals at a handful of sites spanning the Chalcolithic period. Some 157 clay sealings with stamp seal impressions, along with 39 stone stamp seals and 46 geometric stone and clay tokens come from the later fifth millennium BC site of Tall-e Bakun A in Fars (Alizadeh 1988, 2006: 83–90), arguably from an administrative quarter. Most sealings are from door pegs plus a range of portable containers (Figure 9), and Alizadeh interprets the practices here as signifying a disruption to traditional kinship systems by a small group of high-status individuals controlling the flow of commodities through use of seals, tokens, and sealings. Thirty-one tokens and a possible tally piece from Tal-e Mash Karim in north Fars are also indicative of basic accounting practices in this region (Niknami, Taheri, and Sardary 2018), continuing into the Late Chalcolithic at sites such as Tappeh Mehr Ali with finds of five stamp seals and 15 clay sealings, all from portable containers (Sardari 2013), suggesting a system of control over movement of (unknown) commodities in sealed containers.

Quantities of stamp seals, sealings, and tokens from highland Iranian sites suggest steadily increasing levels of at least basic accounting practices through the Middle-Late Chalcolithic (Vidale, Fazeli Nashli, and Desset 2018: 28–29). Thus, at Alou on the Qazvin plain a total of 158 tokens and 91 clay sealings, but no seals, were recovered (Niknami, Moghimi, and Davoudi 2020), while at Qara Tepe on the Qomrud plain unknown numbers of stamp seals in the style of Susa A are reported, in association with evidence for copper working and ceramic production (Kaboli 2005). At Tepe Karvansara west of the Qazvin plain, finds include three clay disk tokens, a single stamp seal, and a single clay sealing found in association with craft-working evidence relating to ceramic and metal production (Alibaigi et al. 2014; Khosravi and Niknami 2021).

These trends are evident at the same time in the Central Zagros region with the increasing use of seals and sealings at a range of sites including Seh Gabi where seven geometric stamp seals, two clay sealings, and 61 clay tokens were found (Hamlin 1974; Henrickson 1988; Schmandt-Besserat 1992a: 43–46). As at Zagheh, Qara Tepe, and Karvansara, the clay tokens at Seh Gabi, along with the seals and sealings, were found in contexts associated with craft production, including ceramics, chipped stone, textiles, and metal-working (Hole 1987a: 50; Rothman and Badler 2011). At Tepe Gheshlagh tokens include 31 clay examples in four different forms plus 14 small spheres in stone (Motarjem and Sharifi 2015: 34–35), while at Tepe Giyan 41 stamp seals, nine cylinder seals, and 14 tokens in clay and stone come from early excavations at the site (Contenau and Ghirshman 1935; Caldwell 1976). The lack of clay sealings from Giyan may reflect the focus on large-scale exposure excavation at the expense of fine-scale recovery of clay objects. It is also likely that some of the seals originate from post-Chalcolithic levels at the site. The briefly excavated site of Dehsavar, southeast of Kermanshah, yielded eight clay sealings with stamp seal impressions plus two stamp seals of later fifth to mid-fourth millennium BC date (Pollock et al. 2020). The practice of depositing stamp seals in human burials, a strong Iranian tradition, is early attested in the Middle Chalcolithic by seven seals at Dum Gar-e Parchineh, nine seals at Hakalan, both sites in southern Luristan (Vanden Berghe 1974; Haerinck and Overlaet 1996), and by five seals from burials of similar date at Tol-e Chega Sofla on the Zohreh plain (Moghaddam 2016).

At Tepe Sialk level IV_1_ of Late Chalcolithic date, relevant finds include some 18 numerical or numero-ideographic tablets, six clay and two stone tokens (Schmandt-Besserat 1992b: 49–50), 11 clay sealings, and seven cylinder seals (Pittman 2013: 329; Abbasnejad Seresti and Tashvigh 2016). These finds align Tepe Sialk in some manner with the wider world of Late Susa II/Late Uruk in the latter centuries of the fourth millennium BC, interpreted by Algaze as an Uruk control post concerned to monitor movement of cherished materials and commodities (Algaze 1993: 55–56). To the northeast at Tepe Hissar, Chalcolithic levels have yielded 57 stamp seals from Hissar IA-IC, seven stamp seals from Hissar IIA–IIB, and at least 14 clay and stone tokens in a range of forms (Schmidt 1937). There are no examples of Late Chalcolithic or Proto-Elamite inscribed tablets from Tepe Hissar (Dahl, Petrie, and Potts 2013: 354). A single clay tag or label with possible incised signs from Tepe Hissar comes from a context that may pre-date the Proto-Elamite levels at the site, but it cannot be regarded as a convincing inscribed document (Damerow and Englund 1989: 2 fn 8).

**Figure 10:**
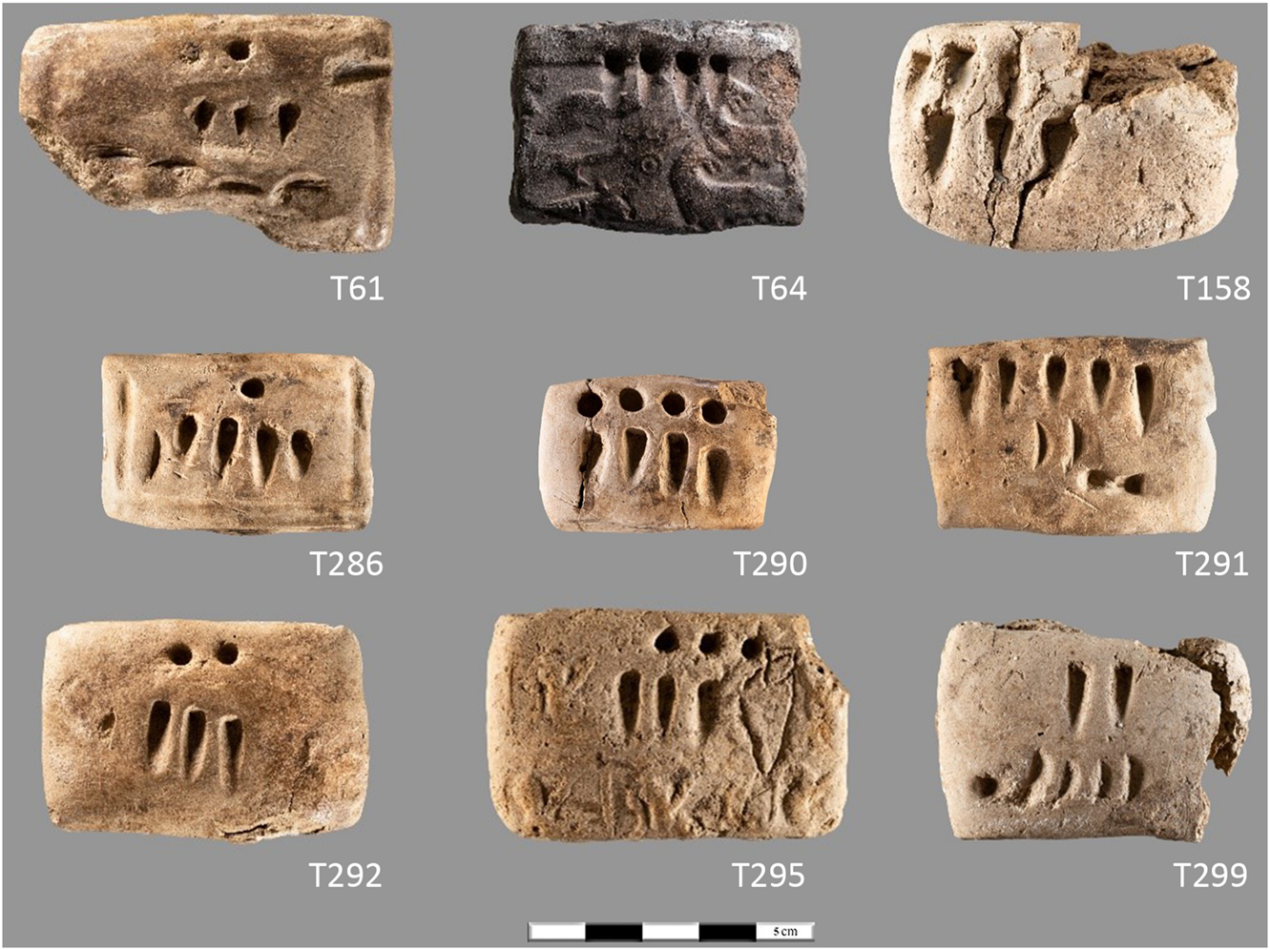
Late Chalcolithic numerical tablets from Godin Tepe (image courtesy of Ali Khayani).

Godin Tepe level VI:1 constitutes one of the most productive arenas for investigation of Late Chalcolithic practices of counting, sealing, and writing (Rothman and Badler 2011; Matthews 2013; Khayani and Matthews in prep.). Within the compound known as the Oval Enclosure, along with ceramics and other objects affiliated to Late Susa II/Late Uruk styles, administrative objects include 43 numerical tablets and fragments, of which four appear to be numero-ideographic and twelve have seal impressions (Figure 10) (Pittman 2011). Additionally, two cylinder seals, eight clay sealings, and two geometric tokens were found in the Oval Enclosure, which is likely to have functioned as a node of regional control for storage and redistribution of agricultural produce from the region, while keeping a protective eye on the important trade routes enabling movement of valued commodities from the rich highland zones to the east to the burgeoning urban centres of Khuzestan and Mesopotamia to the west. Iconographic parallels for the glyptic assemblages at Godin VI:1 appear to span the Late Susa II-Proto-Elamite worlds, suggesting a highly transitional nature to this period of occupation at Godin.

The Chalcolithic bureaucratic assemblages from Chogha Mish and Susa in Khuzestan are of exceptional significance in any discussion of counting, sealing, and writing in early Iran. At both sites we can trace a long trajectory of bureaucratic development, in the case of Chogha Mish starting in the Late Neolithic or Archaic Susiana phase, *c*. 6500–5700 BC, with 19 clay tokens in a range of geometric forms (Alizadeh 2008: 77–78). In the Early Susiana levels of Trench XXI at Chogha Mish, *c*. 5700–5400 BC, at least ten clay and stone tokens associated with an architectural complex of storage and residential structures may relate to the receipt and distribution of foodstuffs stored in large vessels (Delougaz, Kantor, and Alizadeh 1996: 120–125; Alizadeh 2008: 77–78). Middle Susiana levels at Chogha Mish, *c*. 5400–5000 BC, yielded 38 geometric clay tokens also in association with significant architecture, and three geometric stamp seals from uncertain contexts. In the Late Middle Susiana phase, *c*. 5000–4600 BC, a massive monumental building was constructed, with rich evidence for chipped stone production within its rooms (Alizadeh 2008: 42), with five clay tokens and three clay sealings the earliest attested at the site. In the Late Susiana phase at Chogha Mish, *c*. 4600–4000 BC, nine stamp seals and three clay sealings with stamp impressions were found, mainly from insecurely dated contexts. From Middle Susiana Chogha Bonut, four possible counting ‘tablets’ in the form of flat pieces of clay with impressions of fingernails and spheres may constitute basic administrative artefacts (Alizadeh 2003: 85–89).

**Figure 11:**
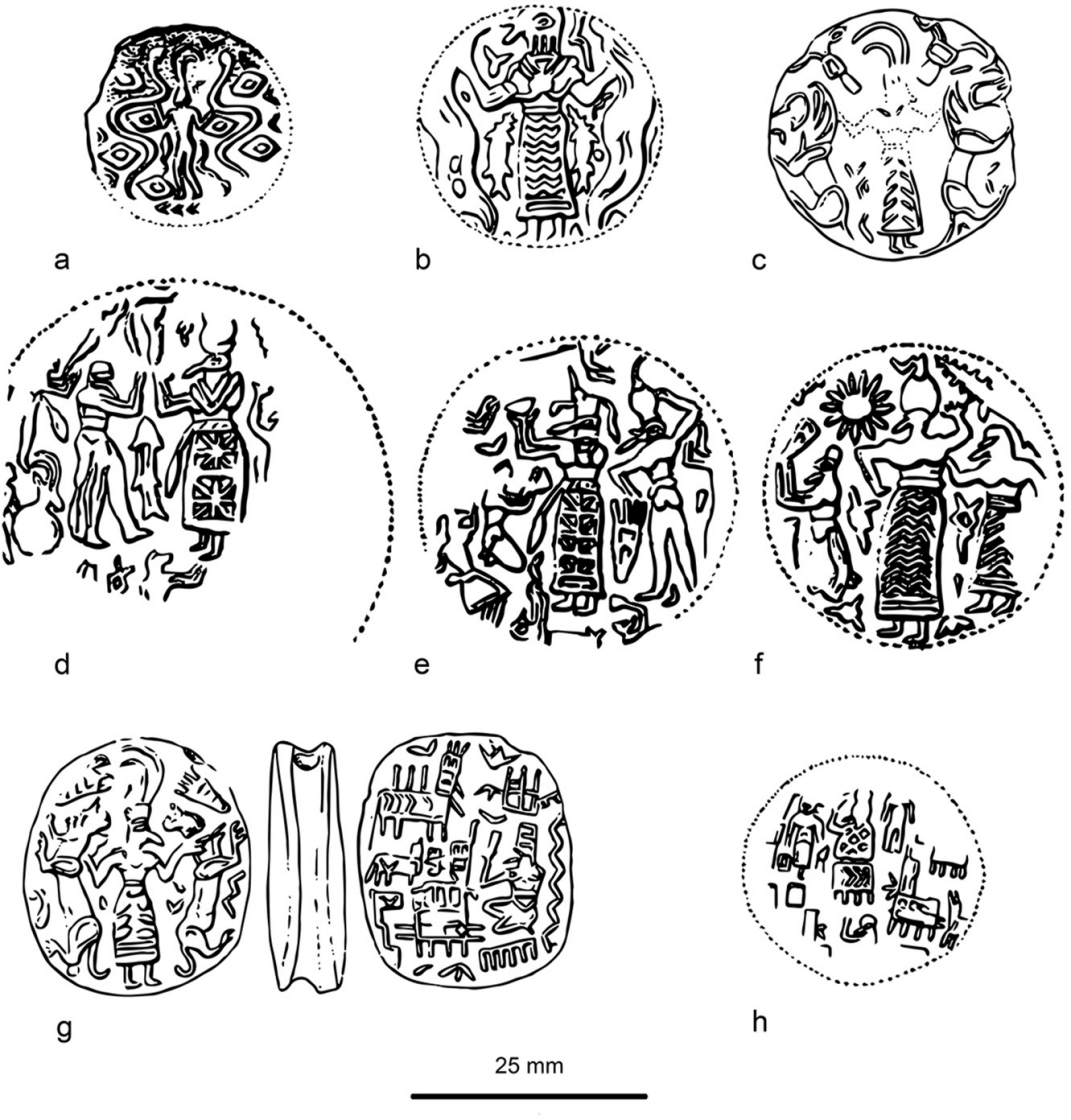
Susa I stamp seal impressions (after Hole 2010: figure 15.8e-l).

Through the later fourth millennium BC, Susa steadily replaced Chogha Mish as the major site of the Susiana plain, a position it retained for several millennia, only being again challenged by Chogha Mish in the so-called Protoliterate or Late Susa II period, discussed below. At Susa during the Susa I period, *c*. 4350–3800 BC, approximately 300 clay sealings, including bullae, with stamp seal impressions were found in a range of contexts, but not in the Susa I cemetery. The largest group was found near a pair of large storage jars and a kiln, as well as in association with both domestic and public architecture (Álvarez-Mon 2020: 20). At least 260 different seals appear to have been in use (Amiet 1972; Hole 2010; Piran 2013), with sealings mainly fixed to pot mouths covered with cloth, or to wooden pegs or knobs securing store-room doors (Amiet 1988; Charvát 1988). Sealings may also be associated with a range of storerooms on top of the *haute terrasse* at Susa. The whole assemblage is arguably associated with the ‘institutionalisation of religion’ through the control of agricultural production on behalf of the temple (Hole 1983: 315). The depiction of deities or high priest figures on the stamp seals and their impressions on the sealing obverse faces lends support to this idea (Figure 11) (Álvarez-Mon 2020: 20–24). A total of some 180 geometric and figurative stamp seals are assigned to Susa I at Susa, although many of them come from insecure provenances and are dated solely on stylistic grounds (Amiet 1972: 38–46, 1973).

Significant evidence in the mid-fifth millennium BC also comes from Jaffarabad on the Susiana plain, including five geometric stamp seals and 72 geometric clay tokens in the form of cones, spheres, disks, and cylinders, once again in association with evidence for craft activity in the form of ceramic production (Dollfus 1973, 1975; Hole 1987b: 86; Schmandt-Besserat 1992b: 23–27, 2018: 370). On the Deh Luran plain, in the Late Susiana period (*c*. 4600–4000 BC), two stamp seals, two sealings, and five tokens at Farukhabad (Wright 1981) indicate village-level involvement in agricultural accounting.

**Figure 12:**
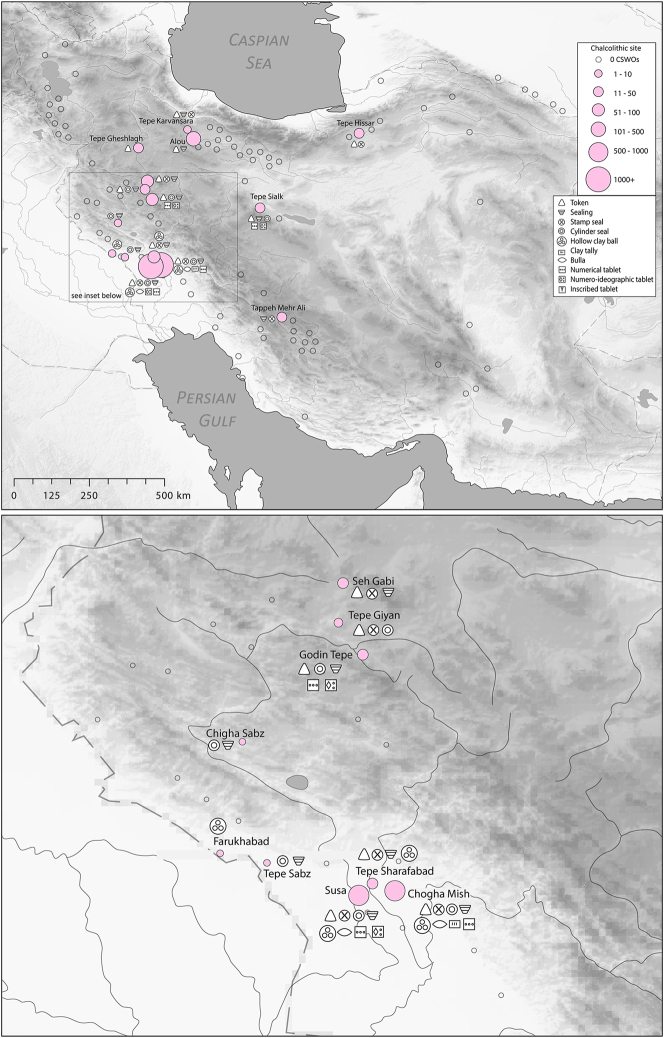
Map of CSWOs from Late Chalcolithic sites of Iran.

In the Late Chalcolithic period, *c*. 4000–3300 BC (Figure 12), four jar stopper sealings with cylinder seal impressions, and two cylindrical bead seals were found at Tepe Sabz (Hole, Flannery, and Neely 1969: 247, 365). At Sharafabad in the Middle Susa II period, *c*. 3500 BC, excavation of a large rubbish pit yielded detailed evidence in the form of eight geometric tokens, three stamp seals, 31 clay sealings with stamp and cylinder seal impressions, largely from containers, and 11 fragments of probable hollow clay balls, together indicating involvement of a small village community in practices of storage of commodities within wider networks connecting them with sites such as Susa and Chogha Mish, possibly concerned with the production of grain by small villages for transport to Susa, as a component of early state formation and control (Wright, Miller, and Redding 1980). Such evidence bolsters Wright’s (1977, 1998, 2013) and Wright and Johnson (1985) notion of the specialisation of centralised decision-making as key to early development of the state, enabling a major expansion in the information processing capacity of the authority in control, while restricting the ability of lower-level social components to challenge authority. The evidence from Khuzestan through the fourth millennium BC fits this picture with the flourishing of technologies of administration and control in the form of seals, sealings, hollow clay balls, and tokens operating as an integrated system of accounting and control. Overall, the Khuzestan evidence can be seen as attesting the rise to dominance of ‘institutions’ capable of fulfilling a role as centralised decision-makers (Benati 2018).

At Susa itself in the Early Susa II period, *c*. 3900 BC, in the Northern Acropolis a possible grain storage facility with charred wheat is associated with complex clay tokens, a stamp seal, and clay sealings with at least seven different seals attested, suggesting control over the storage and distribution of grain (Wright 1985: 732, 1998: 186–188; Wright 2013: 60), an engagement frequently interpreted as fundamental to the development of the earliest states through control of agricultural surplus (Paulette 2013, 2016; Scott 2017). The Late Susa II period, *c*. 3500–3100 BC, as attested at both Susa and Chogha Mish, includes some of the most important developments, of global significance, in the deep-time narrative of counting, sealing, and writing in Iran and its Mesopotamian neighbours. Before the end of the fourth millennium BC, an integrated system of tokens, seals, and tablets enmeshed in a bureaucratic system culminated in the development of true writing in two major cuneiform scripts – proto-cuneiform at Uruk and Proto-Elamite at Susa. The former of these scripts formed the foundation for a 3000 year-long scribal tradition that expanded well beyond its Mesopotamian origins (Englund 1998), while the latter of these scripts, the Iran-based one, endured for a mere few centuries before completely disappearing and leaving no detectable legacy in subsequent scribal and bureaucratic traditions in Iran or anywhere else (Dahl 2019: 56), except perhaps as reference materials for what Dahl calls the schismogenesis of writing in the linear Elamite form almost 1000 years later (debate in Desset et al. 2022; Kelley et al. 2022; Dahl 2023a; see below).

Regarding tokens, at Susa in the Late Chalcolithic exact figures are challenging to arrive at as most of them were recovered in early excavations. Schmandt-Besserat (1992b: 52–91, 2018: 370–372; see also Bennison-Chapman 2023: Table 2) calculates a total of 783 tokens in 16 types and 178 sub-types from Acropole I contexts of Susa II date (levels 23–17 in total), the majority of which appear to come from levels of Late Susa II date, *c*. 3500–3100 BC, including 41 tokens from Le Brun’s 1970s excavations in levels 19–18 of the Acropole I Sounding (Le Brun 2021: 59–62). During Late Susa II, the material culture of Susa shows strong alignment with that of Uruk and other sites of Lower Mesopotamia to the west (Nissen 1985), including in the styles and types of both simple and complex tokens. Many of the Susa tokens were found south of the main Acropole shrine, in an area of workshops and storage facilities, in association with bevelled-rim bowls and clay sealings with cylinder seal impressions (Amiet 1988), thus continuing a long prehistoric tradition of use of tokens within the context of craft and storage activities. The increasing use of complex tokens, with lines and dots incised on a range of shapes, arguably introduced to Susa and Chogha Mish from Uruk to the west, enabled a “quantum jump in the complexity of administration of Greater Susiana” (Schmandt-Besserat 2018: 374), even if their direct connection to the earliest stages of true writing remains under debate.

**Figure 13:**
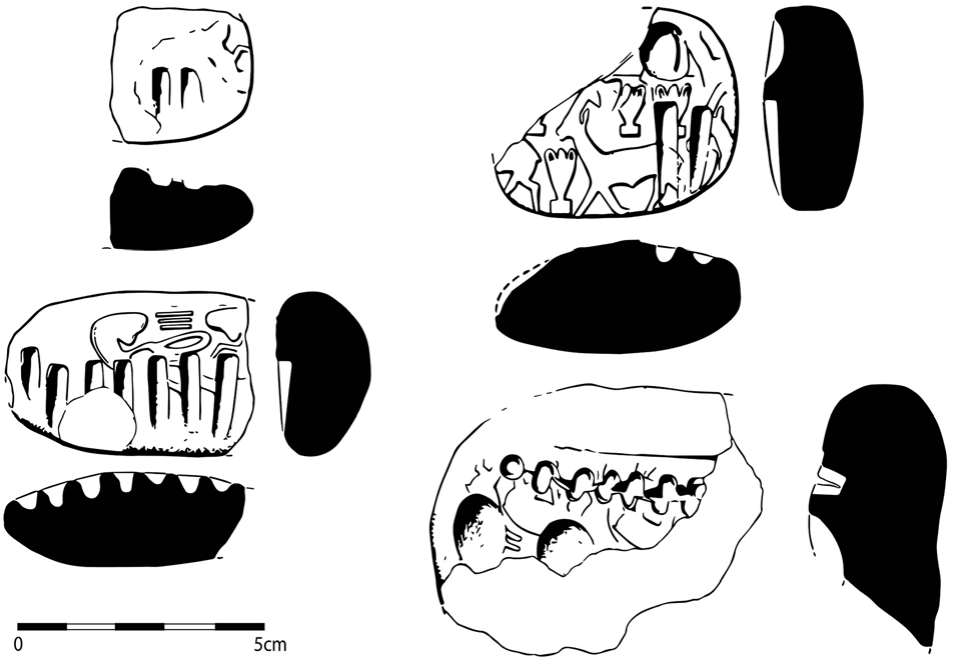
Numerical tablets from Late Susa II levels at Susa (after Le Brun 2021: fig. 16).

The recovery of complex tokens and other elements of bureaucratic control from rather modest, apparently domestic, mudbrick architecture in Le Brun’s excavations of Acropole I levels 21–18 (Le Brun 2021: 51–52) suggests the involvement of individual households in certain forms and levels of administrative activity, once more potentially connected to monitoring of craft production, in this case working in stone (Álvarez-Mon 2020: 35). Le Brun’s careful excavations recovered an intriguing sequence of clay bureaucratic objects from within rooms and in open areas and pits (Le Brun 2021: 59–82; Le Brun and Vallat 1978). A single stamp seal and three clay sealings with stamp seal impressions come from the earliest excavated level, level 21, succeeded in levels 19–18 by a plethora of evidence in the form of 41 clay tokens, 24 sealed bullae (one with impressed number signs), 44 clay sealings mainly from jar necks and all with cylinder seal impressions, one hollow clay ball bearing two different cylinder seal impressions, and six clay tablets bearing number signs and cylinder seal impressions (Figure 13). The Le Brun tablets are similar in all respects to numerical tablets recovered from Acropole I levels 18–17 at Susa in earlier campaigns (Le Brun and Vallat 1978). As Friberg (1994, 485), Englund (1998, 50–55), Potts (1999, 60–61) and others have pointed out, the Susa numerical tablets employ three different numerical notation systems, as opposed to the thirteen attested in contemporary tablets from Uruk, including the sexagesimal S system, the bi-sexagesimal B system and the Š system of cereal capacity notation. Numero-ideographic tablets, bearing number signs plus a single ideographic sign, were found in small quantities (five?) from Acropole I level 17 (Dahl 2013: 242). The finding of a hollow clay ball in Acropole I level 18 fits chronologically with other finds of hollow clay balls at Susa, Chogha Mish, and Farukhabad (Schmandt-Besserat 2018: 375). Finds of hollow clay balls at other Iranian sites, including Sofalin, Qoli Darvish, Tepe Yahya, and Shahdad are less securely dated (Schmandt-Besserat 2018: 375).

**Figure 14:**
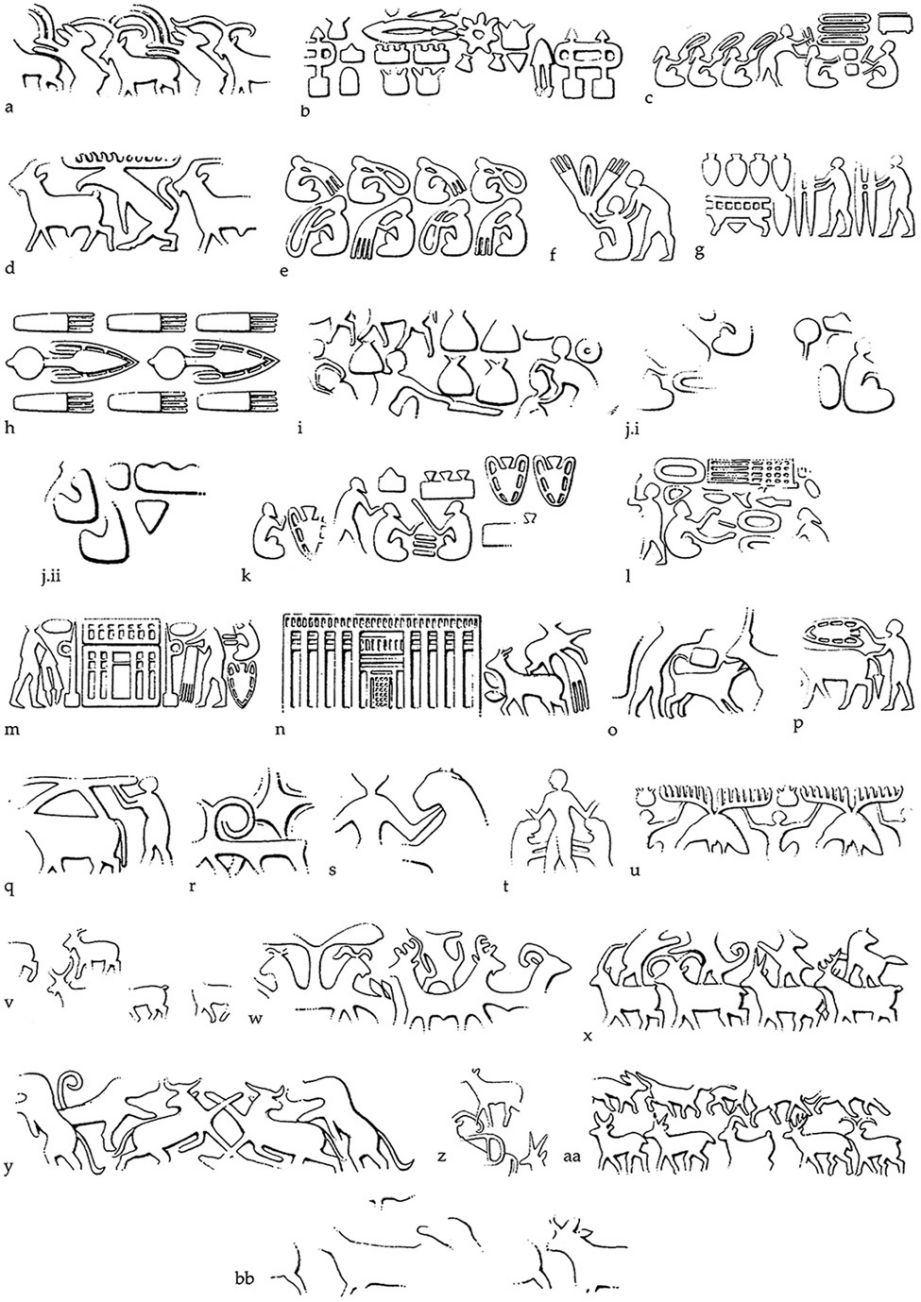
Seal impressions from Late Susa II levels at Susa (after Pittman 2013: fig. 16.10; images courtesy of Holly Pittman).

The Susa II period at Susa sees the steady supplanting of the stamp seal by the cylinder seal, attested from Acropole I level 20 onwards (Pittman 2013: 297). Cylinder seals were used to great effect at Susa, as attested by large quantities of clay sealings bearing seal impressions, the iconography “showing a level of interest in depicting daily activities that would not be matched again in any other period of the ancient Near East” (Álvarez-Mon 2020: 38). Pittman’s (2013) detailed analysis of the glyptic of Susa in this period identifies the earliest scenes as belonging to the so-called ‘baggy style’, also attested at Sharafabad and Farukhabad in the Middle Susa II period. These scenes depict workers making pots, human figures in procession, and conflicts between humans. Many seals show an iconographic development from the Susa I stamp seal styles, but there is a greatly increased range in sealing practices, such as sealing of a wide range of containers, door pegs, hollow clay balls, and numerical tablets (Dittmann 1986, 2012; Charvát 2019). Many scenes depict building construction, grain storage, and the manufacture of goods (Figure 14). Pittman (2013, 319) distinguishes Susa and Chogha Mish glyptic scenes of Late Susa II date from those of contemporary Late Uruk Mesopotamia, above all from Uruk itself as follows: “emphasis in Susa is on production of commodities, while in Mesopotamia the emphasis is more on the movement of goods to an institution and on the activities surrounding the control of a workforce that was ultimately under the supervision of the paramount ruler.” A further distinction between Susa and Uruk in the Late Chalcolithic is that at Susa hollow clay balls frequently bear impressions of clay tokens on their outer surfaces while at Uruk hollow clay balls do not bear such impressions (Boehmer 1999; Englund 2006; Schmandt-Besserat 2018: 376).

From Amiet (1972) and the many other publications of the Susa glyptic materials, it is challenging to arrive at precise numbers of seals and sealings likely to originate from Late Susa II levels at the site in the pre-1970s excavations. We have calculated the following figures for administrative objects from pre-1970s excavations at Susa, likely to be of Late Susa II date: 44 numerical tablets; a total of 112 hollow clay balls or fragments thereof (40 complete, 15 fragmentary, 57 fragments according to Schmandt-Besserat 2018: 375; see also Dittmann 2012; Charvát 2019); four numero-ideographic tablets; at least two baked clay tags with single proto-cuneiform signs; *c*. 220 stamp seals; *c*. 500 clay sealings of various types with cylinder seal impressions in a range of styles, including bullae; *c*. 175 cylinder seals in basic repetitive so-called ‘Jemdet Nasr’ style (Amiet 1972: no’s 715–916).

Chogha Mish is also a key site for the development of early recording and administrative technologies in the Late Susa II period. The site benefits from the fulsome and interpretive publications of the 11 seasons of excavations at the site, spanning 1961–1978 (Delougaz, Kantor, and Alizadeh 1996; Alizadeh 2008). During Late Susa II, Chogha Mish developed into a planned town with public and private buildings, streets, drains, wells, and craft areas for pottery production, in particular. There are traces of a massive thick-walled structure, probably a tower on the High Mound. Many clay tokens and sealings were found close to the tower, suggestive of control over movement of goods in this quarter. The East Area of the Lower Terrace has formal architecture built of classic Uruk *Riemchen* bricks, probably a temple with associated storage facilities. In its surrounding contexts, as detailed below (see also Bennison-Chapman 2023: 224–228), there were major finds of clay sealings, tokens, hollow clay balls, and numerical tablets, suggesting the receipt, storage, and distribution of a range of commodities on behalf of the temple. A group of numerical tablets was found in a small room in a storage facility to the southwest of the temple, which supports this interpretation.

**Figure 15:**
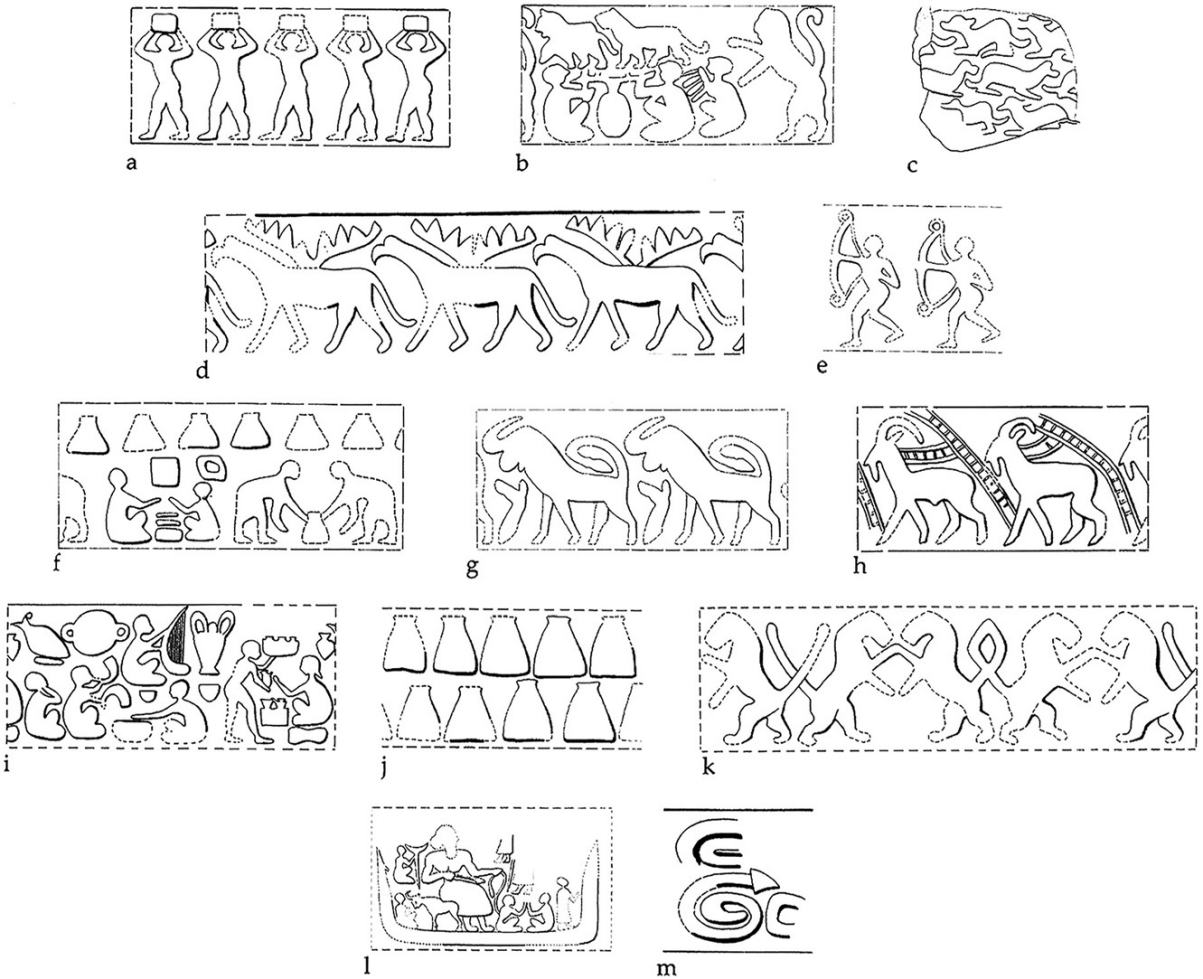
Seal impressions from Late Susa II levels at Chogha Mish (after Pittman 2013: fig. 16.11; images courtesy of Holly Pittman).

Drawing on the evidence published and summarised in Delougaz, Kantor, and Alizadeh (1996) and Alizadeh (2008) (see also Schmandt-Besserat 2018: 370; Bennison-Chapman 2023: 224–225), bureaucratic objects from Chogha Mish during Late Susa II comprise: 901 tokens, mainly of clay, in 10 types and 45 subtypes; 11 stamp seals, mainly with geometric designs; 10 cylinder seals, all with basic repetitive designs; 38 complete and 56 fragmentary hollow clay balls, almost all bearing impressions of more than one different cylinder seal; a total of some 253 clay sealings (including eight bullae; 21 jar neck sealings, 76 door sealings, 70 flat sealings possibly from bales, 78 of unidentified function); six ‘tally slabs’; and 20 numerical tablets. The clay sealings bear the impressions of at least 10 different stamp seals and 125 different cylinder seals, according to the figures in Delougaz, Kantor, and Alizadeh (1996). The designs depicted on the Chogha Mish Late Susa II glyptic show elaborate figured scenes similar but not identical in style and composition to those attested at Susa (Figure 15). They clearly suggest involvement in similar socio-political processes, including the formalisation of cultic practice, as manifest in formal architectural styles, underpinning authority of control over rural production and associated craft activity. Also striking, as at Susa, is firstly the extremely low number of actual cylinder seals recovered from Chogha Mish in Late Susa II, as compared to the high quantities of sealings with cylinder seal impressions, and secondly the lack of stylistic match between the actual seals and the cylinder seal impressions. We must assume either differential disposal of seals as opposed to sealings (perhaps as cherished items in human burials, which have not been located and excavated), use of perishable materials such as wood for seal manufacture, and/or retention of seals through time, including probable recutting for use by future generations of administrators.

### 4.1 Summary of the Chalcolithic CSWO Evidence: Steps Toward Statehood

In summary, the two millennia of the Chalcolithic period across Iran witness a major intensification of administrative activity, manifest both in increased numbers of sites yielding CSWOs and in an increased spectrum of types of CSWO at those sites, that together can be characterised as the material residues of a true information revolution. By the Late Chalcolithic period we can delineate a hierarchy of activity, with Susa and Chogha Mish dominant in terms of quantities and variety of CSWOs, a second tier of sites with up to 100 items, a lower tier with fewer than 50 CSWOs, and contemporary sites from which no evidence for CSWOs has been recovered. Moreover, only at Susa and Chogha Mish do we see a major elaboration of the range of CSWOs in use within their bureaucratic systems, including the intensive adoption of complex tokens in a range of forms that likely reflect increasing precision in accounting for an enhanced diversity of raw materials, finished goods, animals, and animal and agricultural products including wool, textiles, garments, beer, oil, bread, and wood (Schmandt-Besserat 2018: 371).

We do not view these sites across Iran as all participating in a single integrated bureaucratic system, but rather as mainly independent nodes of engagement betokening a groundswell of socio-cultural development region by region. For specific geographical, historical, and cultural reasons, the area of Khuzestan hosted developments unmatched in scale and scope by any other region of Iran, which can plausibly be associated with the early development of state-level societies in this region and in neighbouring Lower Mesopotamia, above all at Uruk. This episode can reasonably be characterised as a genuine information revolution.

## 5 Bronze Age Bureaucracies, 3300–1200 BC

The Bronze Age of Iran, *c.* 3200–1200 BC, hosts a vast panorama of socio-cultural developments, (extensively covered in Matthews and Fazeli Nashli 2022: 188–392), within which the shifting roles of counting, sealing, and writing, as attested by the material evidence, form an intriguing arena for this research. We treat the Bronze Age here in three broad chronological sections: Proto-Elamite/Early Bronze Age, Mid-later third millennium BC, and Second millennium BC/Late Bronze Age.

### 5.1 Proto-Elamite/Early Bronze Age

**Figure 16:**
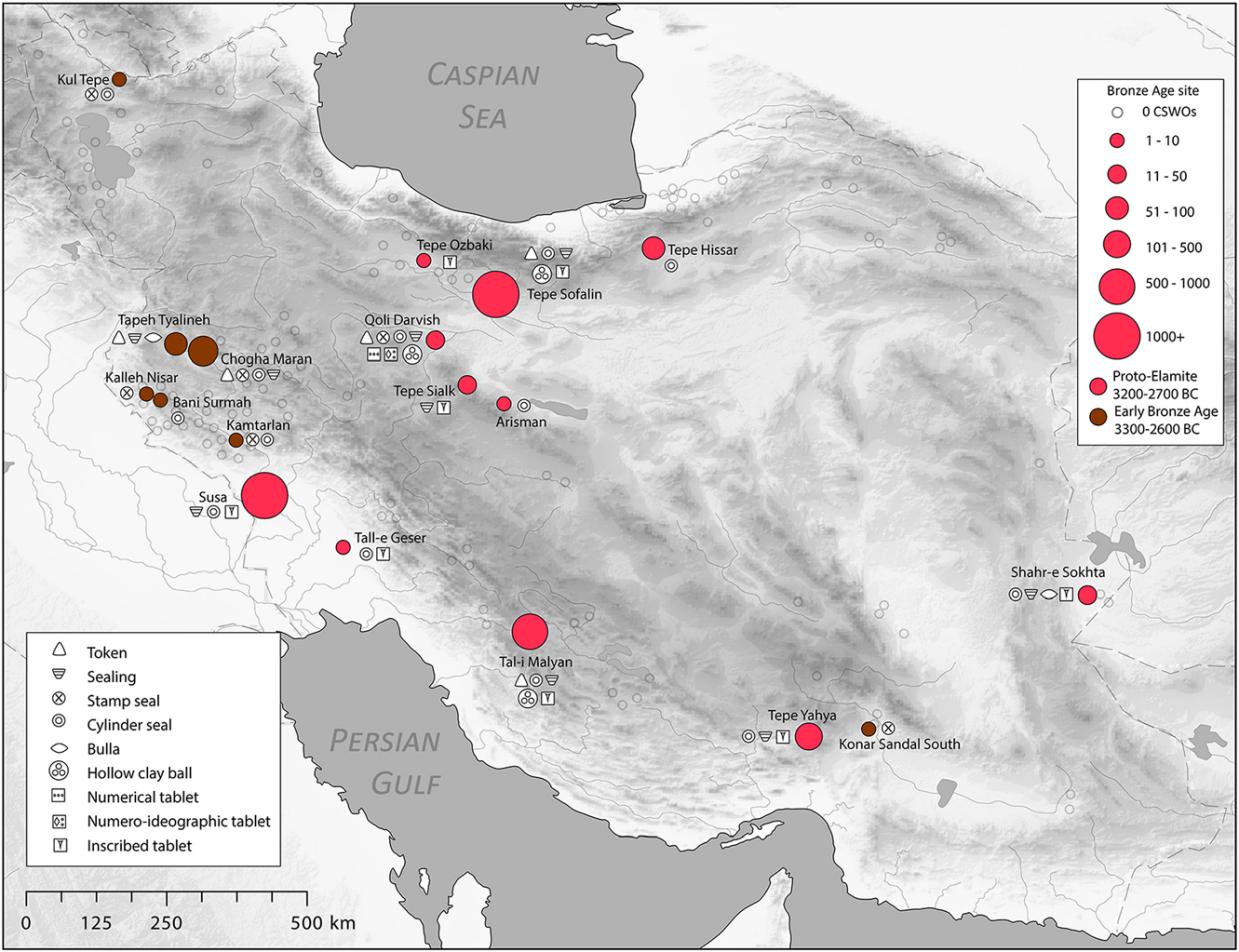
Map of CSWOs from Proto-Elamite and Early Bronze Age sites of Iran.

We commence with a discussion of the evidence from the Proto-Elamite phenomenon that spans the transition from the fourth to the third millennium BC, the early centuries of the Early Bronze Age. As with so much of Iran’s ancient past, the site of Susa is key. Following a short episode of abandonment, at least of the Acropole I area in level 17B-A, levels 16-14B comprise distinctive architecture and material culture that defines the Proto-Elamite culture, designated as the Susa IIIA phase (Abdi 2003). De Morgan’s excavations in 1898–1901 recovered the vast majority of the inscribed tablets that are such a marked feature of the Proto-Elamite world, whose extent reached across much of the Iranian highland zone (Figure 16). Later excavations in the Ville Royale area of Susa also recovered ceramics, seals, and sealings typical of the Proto-Elamite period (Carter 1980). Meanwhile, at Tall-e Geser southeast of Susa, a single Proto-Elamite tablet, four stone geometric tokens, and one cylinder seal were excavated from internal and external architectural contexts (Alizadeh 2014: 45).

While the Late Susa II bureaucratic system is focused heavily on Susiana and Khuzestan, with outliers at Godin Tepe and elsewhere, the Proto-Elamite system is attested across broad swathes of Iran, from Susa to Shahr-i Sokhta. We do not yet understand the mechanics of operation of this administrative system, underpinning as it did a pan-Iranian state-like society with clear codes of conduct as regards its diet (pig taboo? Mashkour 2006), its iconographic representation (pig and human taboo; Dahl 2018: 386), and the formal content and style of its bureaucratic devices in the form of cylinder seals and inscribed tablets.

**Figure 17:**
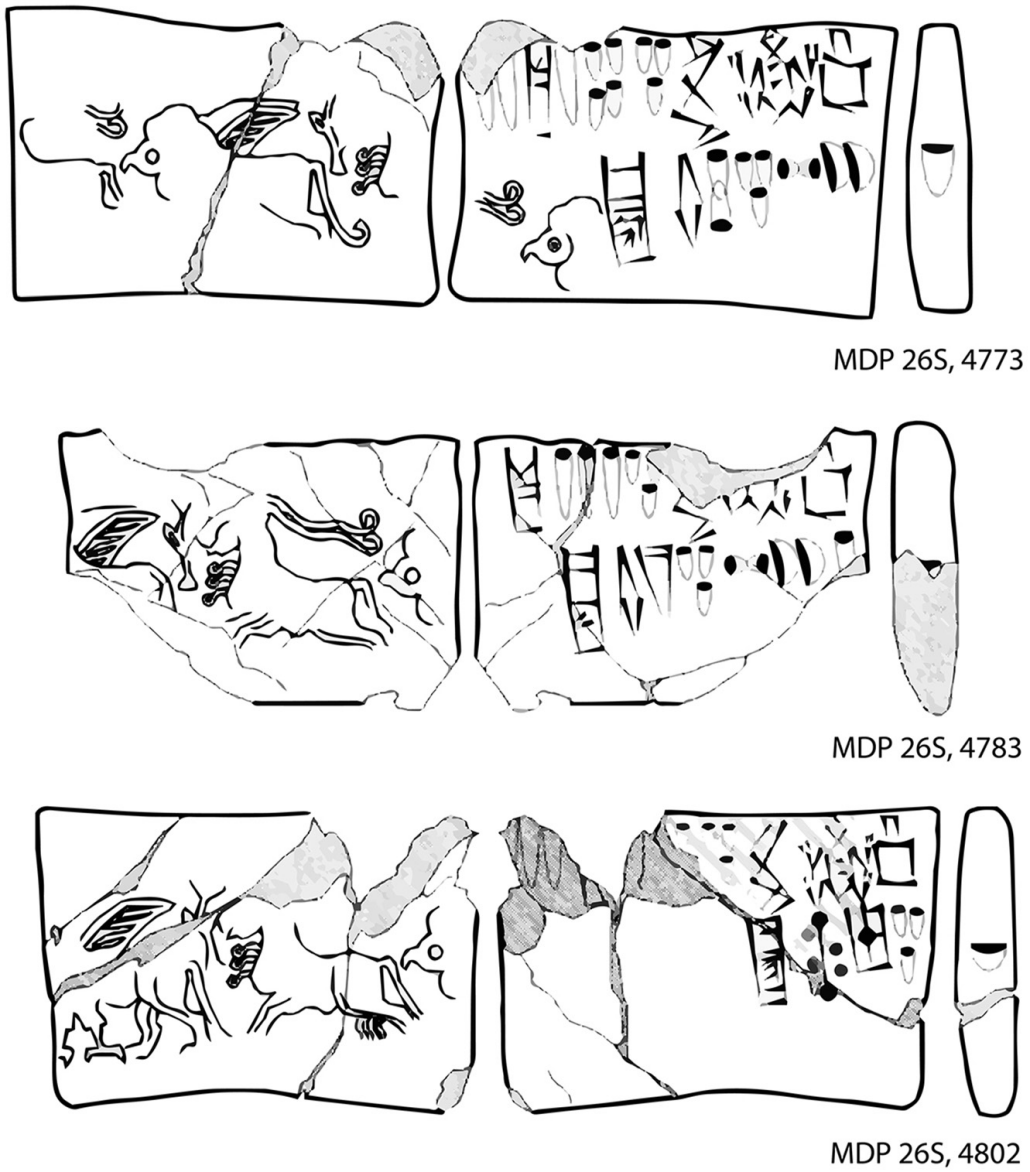
Proto-Elamite tablets from Susa (after Dahl 2012: fig. 4).

In all, some 1560 clay tablets or tablet fragments in Proto-Elamite script were excavated from Susa, almost all of them recorded with minimal or no information on archaeological context (Figure 17). Proto-Elamite tablets from all other sites in Iran added together number fewer than 100 texts (Dahl 2013, 2018, 2019). Proto-Elamite texts, from Susa and from other sites such as Malyan, Yahya, and Sofalin, deal above all with the administration of rural production, including cereal production, flocks of animals, and gangs of labours, occasionally in very large quantities with up to 26,000 animals and >1750 workers attested at Susa, only. Almost all these texts, where understood, are concerned with basic accounting of commodities, with only two metro-mathematical texts known. Counting systems attested in the Proto-Elamite texts show some continuity from texts of Late Uruk/Late Susa II type, employing a total of five basic counting systems (with some additional variants attested at Susa), including three for counting discrete objects, one of which is a decimal system not attested in Uruk IV–III texts of Lower Mesopotamia, and one each for capacity and area measurements (Damerow and Englund 1989: 22–28). A few of the inscribed signs, notably the ‘hairy triangle’ and ‘chunky cross’, have parallel motifs in contemporary glyptic representation, possibly signifying a ruler of Susa or an important institution (Lamberg-Karlovsky 1986; Kelley 2025). Only 16 of the *c*. 1900 distinct Proto-Elamite written signs, built around some 500 basic shapes, occur more than 100 times each across the corpus (Dahl 2002, 2005), indicative of a young and under-developed writing system. The failure of a rigorous scribal tradition to take hold doomed this writing system to disappear along with its supporting political structures at *c*. 2900 BC.

From the same general levels at Susa as the texts, at least 500 clay sealings with seal impressions and *c.* 336 cylinder seals have been recovered (Amiet 1972; Pittman 1997), while some 275 of the inscribed tablets also bear seal impressions. In total, 625 different Proto-Elamite seal images have been identified at Susa, including those attested on tablets and on clay sealings, of which *c.* 50 % are in a figurative style, 40 % in the glazed steatite style, and 10 % in basic wheel-cut and incised styles (Pittman 1994: 83, 1997). Figurative scenes (Matthews and Fazeli Nashli 2022: fig. 7.12) veer sharply away from representation of humans, so common in Late Uruk/Late Susa II glyptic art, to depictions of animals such as goat, sheep, lions, and bulls, but not pigs which are also absent from the textual and archaeological record at Proto-Elamite sites (Mashkour 2006; Dahl 2015, 2023b; Kelley 2025). Susa participated fully in the world of the glazed steatite style, attested across vast regions of Early Bronze Age southwest Asia, from Sistan in the east to the Habur valley in the west, from Nineveh and beyond in the north to Fara and Telloh in the south (Pittman 1994). Only three of 275 sealed Proto-Elamite tablets from Susa have seals in the glazed steatite style, which is much more commonly attested on clay sealings and as actual seals. Human figures are also absent from this style, its motifs typically including hatched arches, crosses, and circles, with stylised depictions of wild goat. Pittman (1994, 212) argues for a Susa origin for the glazed steatite style, with subsequent spread far beyond this core region. The occurrence of stylised hairy triangle and chunky cross motifs in the glazed steatite repertoire connects the glyptic style with the written system of the Proto-Elamite world, a connection also evident in the fact that both the glyptic style and the Proto-Elamite texts disappear at about the same time in the early third millennium BC. The differing geographical distribution of these two mechanisms of administration and identity suggest distinct, but overlapping, networks of engagement across extensive regions of Iran and southwest Asia beyond.

At Susa, the frequency of applying cylinder seal impressions to inscribed tablets of Proto-Elamite type decreases from that attested on Late Susa II numerical tablets, from 61 % (27/44) to 18 % (275/1560) (Pittman 1994: 226). This trend is matched in the transition from Uruk IV to Uruk III proto-cuneiform texts from Lower Mesopotamia (Matthews 1993: 27), and indicates the increased capacity of writing to convey information previously contained in the glyptic iconography (Scott 2018). Associated with this trend, in Lower Mesopotamia as in Iran, is a massive rise in the use of cylinder seals, and occasionally stamp seals, in the practices of impressing clay sealings used to secure store-room doors and a wide range of mobile containers. Major collections of clay sealings have been recovered from many Proto-Elamite sites, including Susa, Malyan, Yahya, Sofalin, Sialk, and Shahr-i Sokhta.

The site of Tal-i Malyan has yielded significant evidence relating to Proto-Elamite administration. In area ABC on the Upper Mound, three cylinder seals, at least 232 clay sealings, mainly from jars, with 34 different seals attested by the impressions, and 15 inscribed clay tablets were excavated from a large building with geometric wall paintings (Pittman 1997: 143). Evidence for craft activity and storage in large vessels may provide the context for the use of accounting devices in this formidable building (Sumner 2015). In area TUV in the Lower Town, more well-built structures contained evidence for craft activity and living quarters (Nicholas 1990). Three cylinder seals, 200 clay sealings, nine hollow clay balls, a few tokens, and 19 inscribed tablets came from this area. In all, 88 different seals are attested in the TUV administrative evidence. Significant variations in both the seal iconography and the clay composition of the sealings from ABC and TUV are suggestive of “a high value placed on controlling and accounting for extremely localized allotments of goods” (Zeder and Blackman 2003: 136), a characterisation probably valid for the full range of Proto-Elamite bureaucratic practices, and supported by geochemical analysis of sealings from Malyan and other Proto-Elamite sites (Yeganeh et al. 2025). A total of 34 Proto-Elamite tablets, nine of them with seal impressions, were found across areas ABC and TUV, their concern to record and account for purely local agricultural and economic activities (Stolper 1985).

**Figure 18:**
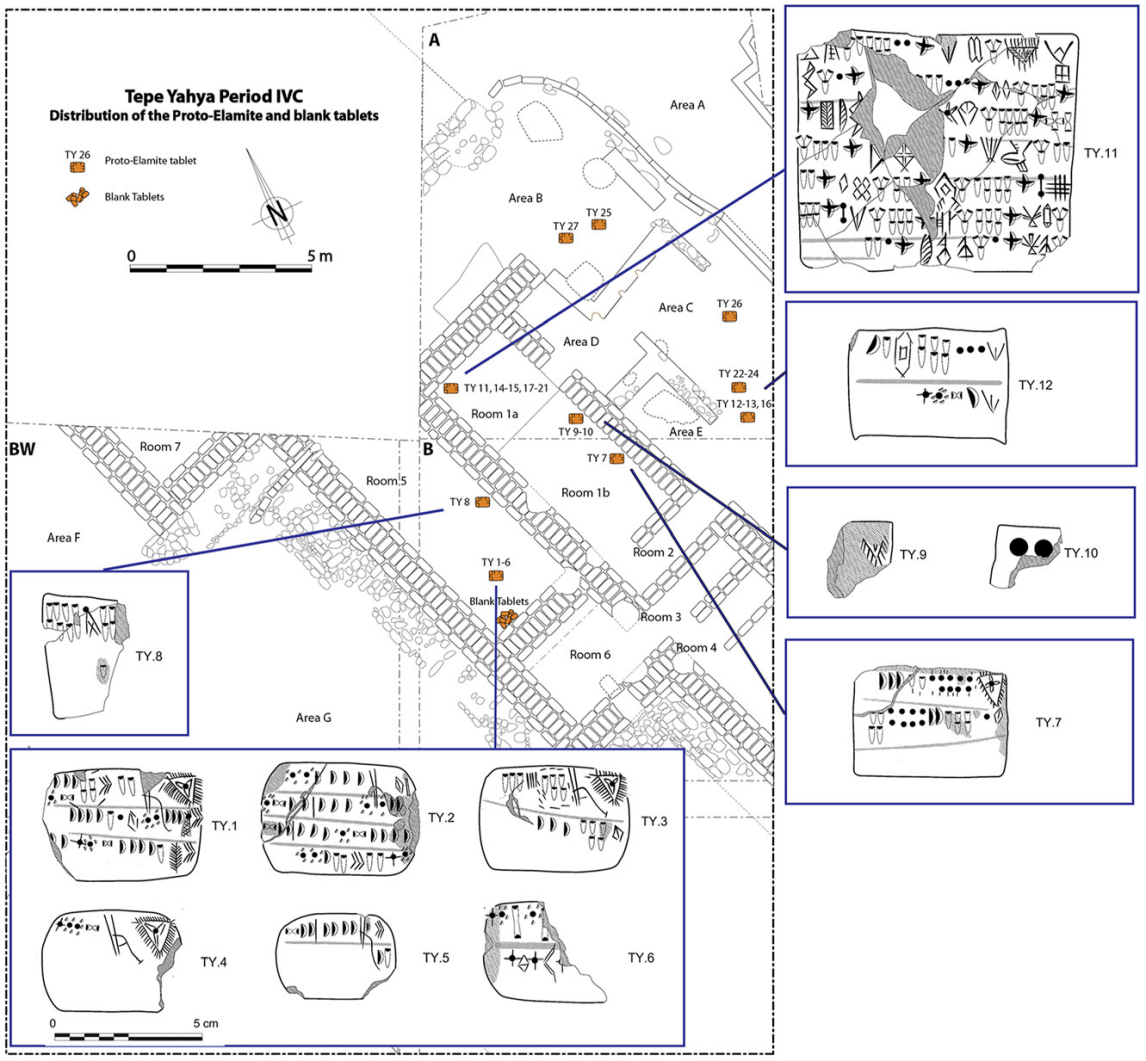
Tepe Yahya, selected Proto-Elamite tablets from period IVC and plan of period IVC architecture with findspots of Proto-Elamite tablets and blank tablets (after Damerow and Englund 1989; Mutin 2013: fig. 5.1; Mutin 2013: fig. 5.3; image courtesy of Benjamin Mutin).

Excavations at Tepe Yahya in Kerman province are also hugely important in providing materials relevant to our study. A major building of level IVC, dating to *c*. 2900–2800 BC (Dahl, Petrie, and Potts 2013; Mutin 2013; Mutin, Lamberg-Karlovsky, and Minc 2016: 851), included on its floors inscribed clay tablets, clay sealings and ‘slingballs’, ceramic storage vessels, and much else besides. Stylistic and material analysis of the ceramics shows considerable levels of trans-regional movement of containers underpinning a high degree of connectivity (Mutin 2013). A total of 27 Proto-Elamite clay tablets, plus up to 88 tablet blanks, were recovered from rooms 1 and 5 of the IVC building and adjacent spaces (Figure 18), fully published by Damerow and Englund (1989). As elsewhere, the texts appear to deal above all with the small-scale management of the rural economy. Only two of the 27 tablets display seal impressions, and only three cylinder seals, in glazed steatite style, were found, as well as some 43 sealings, bearing impressions in both the glazed steatite and classic figured style (Pittman 1994: 98–102, 1997: 144–145; Mutin 2013: 170–172). A total of 44 different seals are attested on the sealings and tablets. Both door and container sealings were identified, and a concentration of jar sealings in rooms 3–4 indicates their use as storerooms. Further east still, at the site of Shahr-i Sokhta in Sistan a single Proto-Elamite tablet and some 35 clay sealings, mainly from door pegs rather than containers, with cylinder seal impressions, one clay bulla, and at least one cylinder seal were found amongst domestic architecture (Amiet and Tosi 1978; Dahl, Petrie, and Potts 2013: 359–360; Ameri 2022: 13–14). The glazed steatite and other styles are attested on the seal impressions (Amiet 1983).

In north-central Iran, along the northern fringes of the central plateau, a single Proto-Elamite tablet has been found at Tepe Ozbaki (Madjidzadeh 2001: 141–145), while larger quantities of tablets (at least 16), seals, sealings (probably in the hundreds), one hollow clay ball containing tokens, and *c*. ten geometric tokens have been excavated at Tepe Sofalin 35 km southeast of Tehran (Hessari 2011; Dahl, Hessari, and Zoshk 2012; Hessari and Yousefi Zoshk 2013). The tablets appear to deal entirely with local agricultural concerns. At Qoli Darvish five numerical tablets, one numero-ideographic tablet, one clay token, one hollow clay ball containing three tokens, one stamp seal, one cylinder seal, and five clay sealings were found within a substantial rectilinear structure (Alizadeh, Aghili, and Sarlak 2013). At Tepe Sialk some seven tablets are in basic Proto-Elamite style, along with unknown numbers of clay sealings (Dahl 2018: 383–384), while three Proto-Elamite style cylinder seals come from Arisman (Helwing 2011: 274–276). At Tepe Hissar two cylinder seals show Proto-Elamite affinities (Pittman 1994: 107). Surface collection at Tapeh Tyalineh on the Mahidasht west of Kermanshah, at the western limits of the Proto-Elamite world, recovered an assemblage of clay sealings from probable trash deposits, comprising 52 jar sealings, 12 door sealings, and three clay bullae (Khosravi et al. 2024). In total, some 27 different seals, the majority cylinder with a few stamps, are attested on the obverse faces of these sealings, which compare well with Proto-Elamite glyptic scenes from excavated sites such as Susa, Malyan, Yahya, and Sofalin. Several of the Tyalineh sealings bear impressions of cylinder seals in the glazed steatite style. Three clay tokens were also found at Tyalineh.

**Figure 19:**
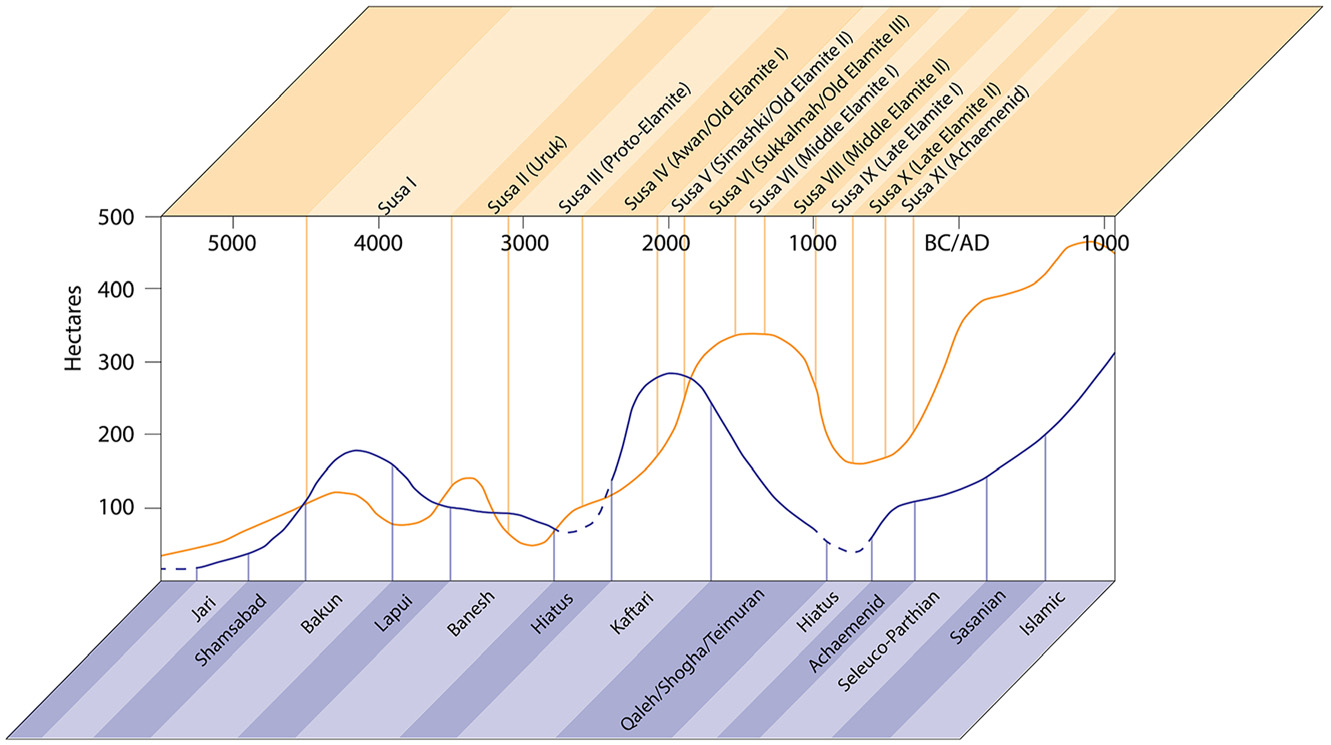
Settlement trajectories through time for the Kur River basin (lower) and Susiana (upper), showing occupied areas, in hectares, per period (adapted from de Miroschedji 2003: figs. 3.2–3.3).

A notable feature of the Proto-Elamite settlement pattern across Iran is the apparent abandonment of rural settlement, matched by a trend of agglomeration into major regional centres (Figure 19), a pattern which has been seen as representing responses to a sudden onset of adverse climatic conditions at *c*. 3200 BC (Staubwasser and Weiss 2006). Proto-Elamite communities living in these regional centres were clearly concerned to record and account for, above all, the agricultural production of their hinterlands, utilising an integrated system of bureaucracy involving cylinder and stamp seals, clay sealings, hollow clay balls, clay tokens, and inscribed clay tablets (Matthews and Fazeli Nashli 2022: 230–235). Only at Susa do the texts suggest involvement of more than local scales of commodities, animals, or human labourers.

Several aspects of Proto-Elamite society suggest significant connectivity throughout the major centres of the time, across much of Iran. Architectural parallels in building styles, including standardised mudbrick shapes and sizes as well as building plans, commonalities in ceramic forms and decorative tastes, in seal styles and even in dietary practices (a heavy focus on goat and sheep with a probable taboo on pig and pork) are all suggestive of widely accepted templates of behaviour across the Proto-Elamite world. Also fundamental to Proto-Elamite identity is the pervasive use of inscribed clay tablets as a form of control and administration of rural production, above all (Yeganeh et al. 2025). While the vast majority of texts come from Susa (*c*. 1560), such tablets have been widely found across the Iranian plateau (Figure 16) (Tall-e Geser 1; Malyan 34; Sialk *c*. 7; Ozbaki 1; Sofalin >16; Yahya 27; Shahr-i Sokhta 1). The location of these sites along the northern and southern routes skirting the central Iranian plateau suggests a concern to protect routes of communication and perhaps also valued commodities being moved along these routes, although the texts stay firmly silent on what those commodities may have been. Archaeological evidence, however, shows considerable movement of cherished materials such as lapis lazuli, carnelian, turquoise, obsidian, and seashells from outside into the Proto-Elamite sphere.

The collapse of the Proto-Elamite world can be placed at *c*. 2900–2800 BC, attested at Susa by a resurgence of Lower Mesopotamian influence in pottery and seal styles with the rise to regional dominance of the Early Dynastic Sumerian city-states (Potts 1999: 90). At the same time, the evidence for writing in the Proto-Elamite style disappears entirely and apparently abruptly, its demise hastened by the absence of a scholarly scribal tradition in contrast to Mesopotamia where lexical texts existed from the very start of proto-cuneiform writing (Dahl, Petrie, and Potts 2013: 375). After *c*. 2900 BC, writing disappears from Iran for half a millennium, longer still in central and eastern Iran. This hiatus in evidence for writing is matched by major falls in human settlement across much of Iran for the period 2900–2300 BC (Figure 19), a significant rupture in village and urban life that is likely connected to environmental factors including severe and lengthy cold and dry spells in the early third millennium BC (Schmidt et al. 2011; Islam, Amirkhiz, and Niknami 2020). It is also plausible that the individual people comprising the Proto-Elamite world decided themselves to reject state-level involvement in their everyday affairs with its pernickety fussing around their agricultural activities, and deliberately chose to return to less intensive and more flexible modes of socio-economic existence (Lamberg-Karlovsky 2003), including occasional reversions to seasonally-based pastoral nomadism and other forms of food procurement and curation that have left so little trace in the archaeological record.

Chronologically overlapping with the Proto-Elamite phenomenon, across much of north-western Iran similarities in settlement patterns, architecture, and ceramics together define the Early Transcaucasian Culture (ETC) also known as the Kura-Araxes Horizon (Matthews and Fazeli Nashli 2022: 236–259). This distinctive cultural phenomenon is attested from the later fourth millennium BC onwards across vast regions of the Caucasus, western and north-central Iran, and eastern Anatolia, and is characterised by mainly village-level settlement, and a lack of evidence for complex, multi-functional settlements. Especially notable is the very limited evidence for even basic bureaucratic or administrative practices across the entire ETC world, with notable exceptions in northwestern Iran including one stamp seal and one cylinder seal from Kul Tepe (Abedi 2016, 2022), despite such practices being well-established within neighbouring cultural systems, including the worlds of Late Susa II/Late Uruk and Proto-Elamite. We have virtually no seals, stamp or cylinder, no tokens, and no numerical, proto-cuneiform, or Proto-Elamite tablets from known sites of this period. One interpretation is that the low-key, household-based, heterarchical nature of ETC society meant that there was simply no role for writing or other forms of accounting to fulfil.

Strategically located on the Great High Road, the Early Bronze Age site of Chogha Maran has yielded a remarkable collection of 50 clay tokens, 149 clay sealings with seal impressions, plus 55 without seal impressions (Renette, Khayani, and Levine 2021: 41), used principally to seal portable containers including baskets, pots, and bags, with a few door-peg sealings also evident, all dating to *c*. 2900–2700 BC (Khayani and Niknami 2020, 2023; Renette, Khayani, and Levine 2021). The seal impressions are made principally by cylinder seals with a few stamp seals also attested. Iconographically, the impressions are largely in a local style, with a small proportion of glazed steatite impressions. A single stamp seal and four fragments of baked clay and shell cylinder seals are the only actual seals found. As far as can be detected, each of the 149 sealings has been impressed by a different seal, 12 stamps and 137 cylinders in total, with no evidence for repeated use of any of the attested seals, a pattern suggestive of “a communal administration with many participants, rather than a centralised system with a few powerful officials” (Khayani and Niknami 2023: 2). But the extremely fragmentary nature of the assemblage clearly renders it difficult to match up small segments of individual seal impressions into more integrated iconographic reconstructions. This corpus attests the presence at Chogha Maran of an administrative system involving the receipt, storage, and redistribution of unknown commodities within a sophisticated economic network, likely engaged in movement of materials along the Great High Road connecting the uplands of Iran with the Mesopotamian plains to the west. The iconography of the seal impressions (Pittman 2013), with their connections to the nascent urban elites of Mesopotamia as well as to the much broader ‘glazed steatite’ trans-Tigridian world, all support such a cosmopolitan interpretation.

### 5.2 Mid-Later Third Millennium BC

**Figure 20:**
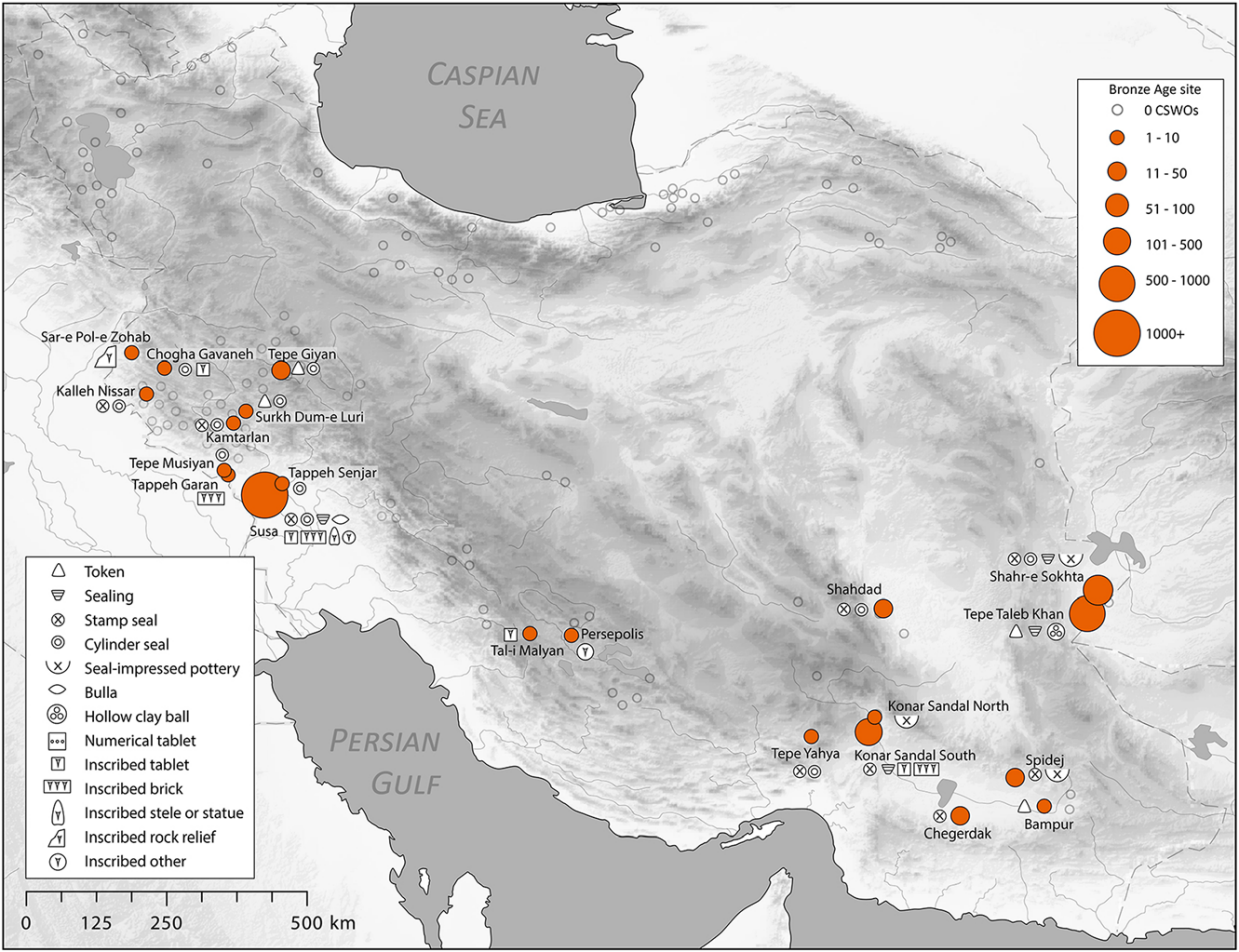
Map of CSWOs from sites of mid-later third millennium BC in Iran.

During the Early Bronze Age, the societies of south-eastern Iran participated fully within a world of trans-regional ‘interaction spheres’ connecting Upper Mesopotamia in the west with the Indus valley in the east, and Central Asia in the north with both shores of the Persian Gulf in the south, together characterised by Possehl (2007) as the Middle Asian Interaction Sphere (Frachetti and Rouse 2012). Archaeological investigations of sites across the region demonstrate mobility of considerable quantities of desirable raw materials and finished goods, including alabaster, chlorite, carnelian, turquoise, lapis lazuli, agate and, above all, metal ores (Weeks 2016). What is the evidence for bureaucratic control or monitoring of this highly productive activity (Figure 20)? Notably, there appears to have been virtually no role for writing in administering this episode of trans-regional trade and exchange. By contrast, the use of seals was clearly of major importance, as stressed by Pittman (2018a, 34): “in an environment where writing, if it existed at all, was not used administratively, seals played a vital role in differentiating the various actors who came to central places or markets to acquire, disperse, or exchange raw or semi-processed materials and certainly also finished goods.”

At Konar Sandal South, some 342 clay sealings in rubbish deposits were associated with an administrative quarter and craft activities, dating to the mid-third millennium BC (Madjidzadeh and Pittman 2008). They come mainly from containers such as bags, boxes, baskets, and jars, with seal impressions made by both cylinder seals and stamp seals. About 12 stamp seals were found but few or no actual cylinder seals. The variety in iconographic styles of the impressed seals, more than 150 in total, underline the interregional connectivity of the site’s occupants, suggesting the presence at the site of agents, merchants, or representatives of diverse communities ranging from Lower Mesopotamia to Central Asia, using their regionally distinctive seals in process of control and administration of storage and movement of highly- valued commodities, such as the semi-precious stones so well attested at Konar Sandal South (Pittman 2019). Much less clear is the possible significance of four incised Geometric and Linear Elamite objects from Konar Sandal, probably locally devised and dating to the late third millennium BC (Desset 2014, 2018a). Four pottery sherds bearing impressions of stamp seals found at Konar Sandal North have their closest parallels in levels of later third millennium BC date at Shahdad (Madjidazeh and Pittman 2008: 101).

At Tepe Yahya in levels IVB-IVA, *c.* 2400–1900 BC, seals show a local style less influenced by Mesopotamian iconography as well as connections eastwards to Central Asia (Ascalone 2013, 2018), with seven cylinder seals and three stamp seals all manufactured of locally available chlorite. These delicately crafted seals illustrate the role of Tepe Yahya in the later third millennium BC as a key node in the production of carved chlorite artefacts in the *série ancienne* or Halil Rud/Jiroft style (Pittman 2018b). The lack of clay sealings from excavated levels of Yahya IVB stands in stark contrast to the seals versus sealings ratio of the preceding Proto-Elamite level IVC at Yahya, and suggests a decentralised, low-key organisation of chlorite artefact production at the site in level IVB, in keeping with the picture proposed for Shahr-i Sokhta as discussed below. Later third millennium BC graves at Shahdad have yielded six cylinder seals and two chlorite stamp seals, as well as copper stamp seals comparable to examples from Shahr-i Sokhta in Sistan (Baghestani 1997; Winkelman 2000; Ascalone 2011) and Spidej and Chegerdak in the eastern Jazmurian basin (Heydari, Desset, and Vidale 2018). There is no evidence for bureaucratic use of any of these seals.

Lavish use of incised and impressed pot-marks is attested at Shahdad, with some 1100 sherds bearing over 600 different signs (Hakemi 1997: 64–69). While a few of the pot-marks bear superficial resemblance to inscribed Proto-Elamite signs, the discrepancy in both date and physical medium renders any significant connection highly improbable. Similar painted, impressed, and incised marks occur on ceramics from third millennium BC levels at Shahr-i Sokhta (Tosi 1983: 144–145). Ongoing systematic study of the full Iranian corpus of incised and impressed pot-marks of the Early Bronze Age, as for example conducted by Glatz (2012) on pot-marks of Late Bronze Age Anatolia, will certainly aid in understanding how such marks may have been situated, if at all, within broader structures of administration and craft production.

Significant evidence for seals and their uses comes from third millennium BC levels of the major site of Shahr-i Sokhta in Sistan (Baghestani 1997; Cattini 2000; Ameri 2022). Some 225 stamp seals and 11 cylinder seals have been recovered from surface collection at the site, most of which can be dated stylistically. Following the Proto-Elamite collapse at the site (see above), a shift in use of cylinder seals in period I phase 10 to exclusive use of stone and bone stamp seals in period I phases 9–8 suggests a significant rearrangement of administrative activity (Ferioli, Fiandra, and Tusa 1979; Fiandra and Pepe 2000). Especially striking is the fact that the elaborate and extensive craft activity at the site in period II, *ca*. 2800–2600 BC, focused on copper and bronze metallurgy, calcite vessel production, and large-scale working of lapis lazuli, carnelian, and turquoise (Lazzari and Vidale 2017), is not accompanied by any evidence for systematic control of that activity: “As far as the archaeological record is concerned, the craft activities may well have been carried out by independent groups or individual craftsmen without any control, accounting concern or formalized duty” (Vidale 2017: 309).

By contrast, in other areas of Shahr-i Sokhta during period II there is rich evidence for use of stamp and cylinder seals. Contextual analysis of that evidence suggests “a system where sealing was used primarily for local storage and commerce” (Ameri 2022: 12). For seals recovered from excavated contexts of period II at Shahr-i Sokhta, the vast majority of them come from graves, with both stamp and cylinder seals deposited with individual bodies. In contrast to the preponderance of burial contexts for actual seals, clay sealings in period II Shahr-i Sokhta come from architectural contexts such as the House of the Stairs in the Eastern Residential Area and the House of the Jars in the Central Quarters or in stratified fill deposits overlying these houses, which also contain unknown quantities of clay tokens, principally cones, bicones, and spheres (Cattini 2000). In the House of the Stairs, a total of 46 clay sealings were found with a range of stamp seal impressions, interpreted by Ameri (2022, 16–17) on the basis of iconography as evidence for female administrative activity in or near this building. In total, around 140 clay sealings come from period II contexts, along with a total of 45 stamp seals, eight cylinder seals, and one seal-impressed sherd. Ameri’s detailed contextual analysis of the Shahr-i Sokhta period II glyptic evidence proposes a major role for women in administrative activity across household and neighbourhood scales, also suggested by the prevalence of seals in female burials at sites in Turkmenistan. Detailed analysis of human remains from the main cemetery at Shahr-i Sokhta supports an interpretation of women as holding high social status within a matrilocal society devoted to long-range trade (Vincenti et al. 2024). In later levels of the site, reduced administrative activity is evidenced by a few baked clay stamp seals only (Tusa 1977).

The site of Tepe Taleb Khan, only 20 km south of Shahr-i Sokhta, appears to have functioned as a major storage and administrative centre, *c*. 2450–2350 BC (Kavosh 2022; Kavosh and Oveisi-Keikha 2024), with a mudbrick storage depot and an adjacent pit containing some 650 clay objects relating to the counting of commodities in pots, baskets, and other types of containers. Objects include tokens, hollow clay balls, sealings, miniature vessels, and human and animal figurines, apparently used together in a system of input, storage, and output of unknown materials and commodities. Most of the sealings lack seal impressions but a couple of stamp impressions show connections to the Bactria-Margiana region to the north-east, while a few stamp seals have extremely basic designs.

From later third millennium BC graves at Spidej in the eastern Jazmurian basin of far south-eastern Iran, six copper and silver stamp seals show connections to Shahdad, Tepe Yahya, and Konar Sandal South (Heydari, Desset, and Vidale 2018), while surface recovery of 19 potsherds bearing impressions of stamp seals suggests a role for these seals in the marking of pots for purposes of identification or ownership of some form. These stamped pots are also likely to have been deposited in graves, recently looted, so may connect with specific funerary practices rather than with administrative practices of the living. Twenty-three copper stamp seals with geometric designs from disturbed contexts at Chegerdak, to the southwest of Spidej, may also originate from burial contexts of later third millennium BC date (Heydari et al. 2015, 2018). Two geometric clay tokens were found at Bampur (Schmandt-Besserat 1992b: 7).

Overall, the evidence from Bronze Age eastern Iran for bureaucratic activity is largely restricted to stamp and cylinder seals with no evidence for use of writing on clay as a means of administrative control. The absence of evidence for state-level or even city-level engagement in control of economic activity suggests that trade and exchange may have been conducted at lower-scale levels of the household and neighbourhood, each of whom may have owned and used seals as marks of authority within a low-key system of local and regional administration. It is also notable how high a proportion of actual seals from sites across south-eastern Iran come from human burial contexts, rather than from excavated architectural or other types of settlement contexts. This facet further suggests a strong connection between seal use and individual identity, persisting through life and beyond into the grave, which helps to explain the high numbers of different seals attested at these sites.

While the major use of seals in the past of Iran, as elsewhere, appears to be related to administrative activity of some kind, there is also significant evidence for a role for seals as status and votive items. This practice is suggested by the deposition of seals in often elaborate tombs or graves in a range of places and periods of Iran’s past. Continuing a Chalcolithic practice (see above), in the Early Bronze Age of Luristan, nine cylinder seals in a range of Jemdet Nasr-Early Dynastic III styles were placed in early third millennium BC tombs at Bani Surmah (Tourovets 1996), while five cylinder seals and one stamp seal were found at the small settlements of Kamtarlan I and II (van Loon 1989a). Seven Mesopotamian-style cylinder seals and two stamp seals of early second millennium BC type were deposited in reuse of older tombs at Kalleh Nissar (Haerinck and Overlaet 2008: 47–51). At the Late Bronze Age site of Bayazid Abad in north-western Iran, 55 cylinder seals in Mitanni style were deposited in a stone-lined burial containing 15 adult and child individuals and vast quantities of ceramics and metal objects (Amelirad and Khanmohamadi 2016). Late Bronze Age finds at Surkh Dum-e Luri of 30 cylinder seals and 12 stamp seals in Kassite, Middle Assyrian, Middle Elamite, and Mitanni styles are likely to attest cultic and votive depositions within a sanctuary that continued in use into the Iron Age (Schmidt 1989; van Loon 1989b). No clay sealings have been found at any of these sites to indicate actual use of these seals in administrative activity of any kind.

Following the collapse of the Proto-Elamite state at *c*. 2800 BC, there are increasing Sumerian and Akkadian impacts on the material culture of Susa, in levels designated Susa IIIB and IIIC. Ceramics and a few cylinder seal impressions, precise quantity unknown, show strong connections with Lower Mesopotamian sites, including Nippur and Tell Asmar (Voigt and Dyson 1992: 133). During period IVA at Susa, *c*. 2600–2450 BC, at least 22 cylinder seals and 133 clay sealings with seal impressions are characteristic of Mesopotamian Early Dynastic I–III styles (Amiet 1972: 28–33; Álvarez-Mon 2020; pl. 42), and are possibly associated with suites of storerooms on the Acropole. Six cylinder seals from the Vase à la Cachette are locally executed versions of Early Dynastic IIIA Mesopotamian styles (Carter et al. 1992: 108–110).

Some 500 years after the collapse of the Proto-Elamite system, the Akkadian conquest of Susa brought about a resurgence in writing and bureaucracy at Susa, underpinning elite control and administration (Desset 2017: 12–13), accompanied by the adoption of silver as the main medium of trade and exchange (Helwing 2018: 126). Introduction of the Akkadian script to Susa, in period IVB, is vividly illustrated through two inscribed brick fragments of the Akkadian king Naram-Sin which may have marked the construction of a major palace comparable in scale to that attested at Tell Brak in north-eastern Syria (McMahon 2020). Thereafter through the Bronze and Iron Ages, royal inscriptions in various forms and frequently bilingual are produced on behalf of multiple rulers of at least parts of Iran (Malbran-Labat 2018). Some 85 Old Akkadian legal and administrative texts from Susa are augmented by 15–20 inscribed statues, stone stelae, bronze axes, stone mace-heads, and brick fragments from building activity by Naram-Sin (De Graef 2013; Basello and Giovinazzo 2018). This pattern of adding elite dedicatory and self-promoting inscriptions to baked clay and stone objects starts in Iran in the Akkadian period, as a Mesopotamian import, and persists more or less without break into the late Iron Age and beyond. An increased use of cylinder seals in Mesopotamian and Jiroft styles (Ascalone 2018: 628–629, 2019; Álvarez-Mon 2020; pl. 50) is also well attested, with 135 seals of shell and stone (Amiet 1972: 140–153). Only 13 clay sealings come from Susa IVB contexts, of which three bear impressions of seals with Akkadian inscriptions (Álvarez-Mon 2020: 131–132). These changes in sealing practices through time, as attested at Susa in the mid-later third millennium BC, fit within a broader picture of shifting bureaucratic practices across the Akkadian world, doubtless stimulated by the enhanced role of the imperial state in overseeing and controlling people and their productive activities over extensive and diverse geographical regions (Rakic 2018).

The brief episode of Susa VA, including the career of the Elamite king Puzur-Inshushinak, *c*. 2150–2000 BC, sees the introduction at Susa of yet another writing system, so-called Linear Elamite, occasionally used alongside Akkadian (Amiet 1966: 227; Steve and Gasche 1971: 61; Álvarez-Mon 2020: 152). While it may have originated in southern Iran within Kerman province (Desset 2018a: 412), Linear Elamite was strongly promoted by Puzur-Inshushinak and is attested in 32 inscribed objects, comprising 18 objects from Susa (including statue fragments, baked clay cones, stone cultic steps, and two clay/gypsum tablets), one ceramic vessel from Shahdad cemetery, four clay tablets from Konar Sandal South, plus nine objects without provenance, one of which may have been found near Persepolis (Desset 2014, 2018a: table 20: 1). Unprovenanced inscribed silver alloy vessels of so-called *gunagi* form may have been used in special funerary ceremonies (Desset 2018b). Before long, Linear Elamite disappeared as mysteriously as it had appeared at Susa, with the reassertion of Akkadian as the sole significant written language (Stolper 1992). Recent claims of decipherment of Linear Elamite are hotly debated (Desset et al. 2022; Kelley et al. 2022; Dahl 2023a). We here accept Dahl’s (2023b: 117–118) proposal that the invention of Linear Elamite was a form of schismogenesis, “where scribes in Susa, confronted both with Mesopotamian cultural expansion in the late 3rd millennium BC and informed through Puzur-Inshushinak’s conquest of northern Babylonia, used signs from an ancient local writing system [i.e. from Proto-Elamite tablets encountered through digging activities at Susa] as the basis for the signs of their new script … by scribes reacting against the Mesopotamian cultural expansion” (see also Daneshmand 2024 regarding intentional deviation by Elamite scribes from contemporary Mesopotamian scribal practice).

Rare finds of Indus-style stamp seals, with or without brief inscriptions in the Indus script, one each at Susa and Tepe Yahya, plus two examples from somewhere in western Iran, likely date to the time of Puzur-Inshushinak (Laursen 2010). Use of Akkadian for formal inscriptions also continued at Susa during Puzur-Inshushinak’s reign, with dedications to the temple of SHU-GU attested on six baked clay nail fragments, and a series of inscriptions in both Akkadian and Linear Elamite on the stone steps of a podium of the temple of Inshushinak (Álvarez-Mon 2020: 143).

During Susa VB, Susa was incorporated into the Ur III imperial state, with a reversion to texts and seals of Mesopotamian types, now largely in Sumerian rather than Akkadian (De Graef 2013), including 65 cylinder seals, 11 stamp seals, and 54 clay sealings, of which 22 have inscriptions (Amiet 1972: 153–160; Álvarez-Mon 2020: 166). The Sumerian rulers of the Ur III empire, starting with Ur-Nammu, promoted major building programmes at Susa (Malbran-Labat 1995; Steve, Vallat, and Gasche 2002), partly attested by a total of 49 Ur III texts principally in Sumerian with a few in Old Akkadian (De Graef 2013: 268, 2015). Of the 49 Ur III Susa tablets, an archive of 38 tablets was recovered from two rooms of a well-appointed house of a scribe named Igibuni in Ville Royale Chantier B at Susa, dating from Shu-Shin year 4 to Ibbi-Sin year 1, *c*. 2034–2027 BC (De Graef 2005, 2015). These texts are concerned with issues of household economy including loans of barley and domestic expenditures. Construction of temples on the Acropole by king Shulgi, 2094–2047 BC, dedicated to the local deity Inshushinak and to the Mesopotamian deity Ninhursag, are attested by ten inscribed copper foundation nails (Tallon 1987: 308–310; Álvarez-Mon 2020: 164–166), while a bronze hammer and a stone mace-head each bear a Sumerian inscription of Shulgi (Álvarez-Mon 2020: 170). Two Ur III texts in Sumerian found at Malyan in Fars indicate ongoing importance of this region within the context of Ur III administration (Desset 2012: 133–134; De Graef 2013: 269). At about the same time, inscriptions in Akkadian occasionally accompany carved rock relief scenes such as those of Anubanini, king of the Lullubi, at Sar-e Pol-e Zohab (De Graef 2013: 269; Alibaigi et al. 2020). Around six fragments of inscribed bricks, probably of Ur III date, have been found in survey at Tappeh Garan on the Deh Luran plain (Jotheri and Zeynivand 2021), while an inscribed cylinder seal, with an Amorite name, was found at nearby Musiyan (Zeynivand 2019).

### 5.3 Second Millennium BC/Late Bronze Age

Through the period of the Grand Viziers in the Old Elamite period, *c*. 1950–1500 BC, a total of 1438 tablets from Susa, almost entirely in Akkadian, with only three examples in Old Elamite, together attest significant ongoing Mesopotamian influence through these centuries, including school exercise texts in the Mesopotamian tradition (De Graef 2007; Tanret and De Graef 2010; Malayeri 2013). Eleven of the tablets bear seal impressions (Amiet 1972: 160–168). Of these texts, more than 100 were recovered from the house of Temti-Wartash, *c*. 1745 BC, Great Chamberlain of the Elamite palace during the reign of Kutir-Nahhunte I, including several with seal impressions of that king (Álvarez-Mon 2020: 186, pl. 67). A further seven of the 1438 Susa texts relate to pre-burial funerary rites in association with a group of adjacent tombs (Tavernier 2013). Nine monumental brick inscriptions from Old Elamite Susa (Potts 1999: 174) attest ongoing elite commissioning of temple construction. Cylinder seals of the Old Elamite period at Susa can broadly be divided into those showing Old Babylonian Mesopotamian influences and those oriented more towards the Elamite sphere, characterised as ‘Anshanite’. According to Amiet’s (1972) treatment of these seals from Susa, 67 can be classed as Old Babylonian in style, 183 as Anshanite, and 17 as somewhere in between. In all classes, seals are made of a range of materials, including bitumen, faience, lapis, and chlorite. The modest numbers of sealings from Old Elamite Susa include two bullae and 37 clay sealings (Amiet 1972: 176–178). A single Anshanite cylinder seal also comes from Tappeh Senjar, 18 km from Susa (Sardari and Attapour 2019).

**Figure 21:**
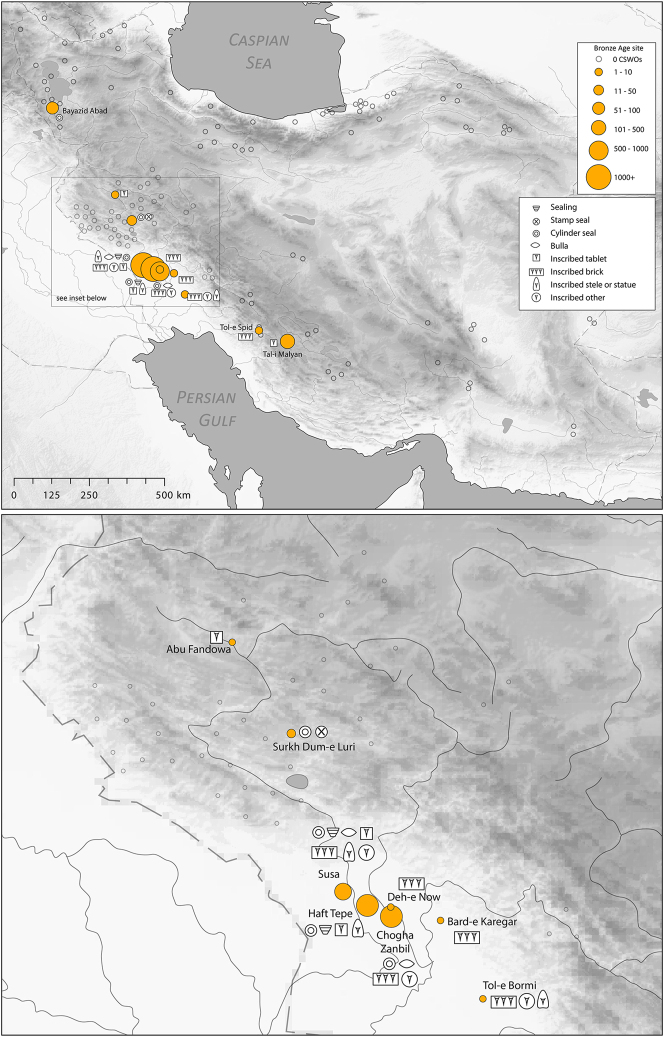
Map of CSWOs from Late Bronze Age sites of Iran.

Resurgent Mesopotamian impact in the Iranian uplands is also attested by an archive of 84 early second millennium BC Akkadian tablets excavated from a single room of a large building at Chogha Gavaneh in the central-west Zagros region (Abdi 1999; Abdi and Beckman 2007; Potts 2020). Of the 84 tablets, five bear the impression of the same cylinder seal. The tablets include lists of rations, and persons including soldiers and slaves. The site of Chogha Gavaneh was clearly significant as a node of highland-lowland communication, arguably involving the movement of tin from Afghanistan or other sources westwards along the Great Khorasan Road with access beyond to major sites such as Eshnunna, Assur, and Mari (Figure 21) (Gentili 2012). A single cylinder seal, found in the same room as the archive, depicts a female figure paying obeisance to a representation of the Storm God Adad (Abdi and Beckman 2007).

The Middle Elamite period at Susa, *c*. 1500–1100 BC, saw ongoing use of inscribed bricks as dedicatory components of massive temple building programmes (Potts 2010; Roche-Hawley 2012), as well as a gradual shift in emphasis from Akkadian to Elamite as the language of high-status pronouncements (De Graef 2013: 276–278). Ghirshman’s excavations in the Ville Royale at Susa recovered more than 120 tablets of early Middle Elamite date, as yet unpublished (Steve et al. 1980: 122–128; De Graef 2013: 276). According to Amiet (1972, 36–37), 42 cylinder seals, 27 clay sealings, and two clay bullae were found in various Middle Elamite contexts at Susa.

Most significantly, the site of Haft Tepe (Kabnak), 10 km southeast of Susa, yielded at least 371 clay sealings with cylinder and stamp seal impressions, many inscribed (Amiet 1996; Mofidi-Nasrabadi 2011; Ascalone 2018: 641; Álvarez-Mon 2020: 234–237). Most of the sealings appear to have sealed jars rather than store-room doors. Actual seals were found in small quantities at Haft Tepe, including two with Akkadian inscriptions from a probably female burial. Some 650 Akkadian clay tablets from Haft Tepe record receipts and disbursements of daily provisions and cherished commodities including metals to workshops (Herrero 1976, 1991; Herrero and Glasner 1990; Basello and Giovinazzo 2018; Helwing 2018: 120). Of the 650 Haft Tepe tablets, at least 14 bear cylinder seal impressions (Álvarez-Mon 2020: 237). Some of the tablets appear to have been stacked on shelfing in a room that may have functioned as a scribal workshop (Mofidi-Nasrabadi 2013: 162). Three fragments of monumental stelae from Haft Tepe also bear inscriptions in Akkadian (Reiner 1973). Four Akkadian tablets of this date were also found at nearby Abu Fandowa (Herrero 1976, 1991), while ‘several’ examples of an Akkadian inscription of the Middle Elamite II king Igi-halki were found at Deh-e Now, 20 km east of Haft Tepe (Steve 1987).

During the reign of the Elamite king Untash-Napirisha, *c*. 1340–1300 BC, extensive evidence of his building activities comes in the forms of inscribed bricks in Akkadian and Elamite from across Elam, including at Susa where at least 25 such bricks were found (Stolper 1992; Potts 1999: 212–215; De Graef 2013: 276; Tavernier 2018) and Tol-e Bormi, ancient Huhnur, in the Ram Hormuz region where at least one Middle Elamite inscribed brick was found (Mofidi-Nasrabadi 2005). At Susa inscriptions are also found on stone beads, stelae, and statues such as the massive bronze statue of queen Napir-Asu. The extraordinary site of Chogha Zanbil (Al Untash-Napirisha), 35 km southeast of Susa, has yielded vast numbers of inscribed clay bricks (up to 6500 of them: Potts 1999: 223), clay nails (several hundred), and glazed clay objects of the king Untash-Napirisha, attesting construction of at least 25 temples (Mofidi-Nasrabadi 2003–2004; Basello 2012). In contrast to other Middle Elamite sites such as Susa, Haft Tepe, and Malyan, no inscribed tablets have been found at Chogha Zanbil. More than 185 cylinder seals from the site, many with inscriptions, appear to come principally from votive deposits (Porada 1990; Álvarez-Mon 2020: 304–311), while small clay ovals or bullae with Elamite inscriptions were found in vaulted tombs of the *palais hypogée* (Steve 1967: 103). There is no evidence for use of the cylinder seals in bureaucratic activities of any kind.

The final phase of the Middle Elamite period, Middle Elamite III, sees a further boom in temple construction at Susa and beyond, attested by more than 450 bricks with inscriptions of Shutruk-Nahhunte and his sons found at Susa and beyond (Potts 2010, 2016: 225), including at Bard-e Karegar east of the Karun River (Moghaddam and Miri 2007: 40). These inscriptions are more commonly in Elamite than Akkadian (De Graef 2013: 277; Tavernier 2018). Inscribed objects found at Susa in this period include many classic monuments taken from Babylonia as booty, such as the victory stele of Naram-Sin, Hammurabi’s Law Code stele, and Kassite boundary stones (*kudurrus*) (Harper and Amiet 1992), as well as inscribed items brought to Susa from Al Untash-Napirisha and Anshan (Garrison 2012). Around 70 late Middle Elamite tablets were found in Ghirshman’s excavations in the Ville Royale at Susa, none of which have been published (Steve et al. 1980: 119–122; De Graef 2013: 278).

In Late Bronze Age Fars, a massive collapse in regional settlement (Figure 19) – site numbers halved, occupation at Malyan down from 130 to 40 ha – is accompanied by the occurrence of writing as attested by 282 cuneiform tablets found in the EDD building at Malyan, dated to *c*. 1100 BC, a return of writing to the region some 900 years after the Ur III episode and 1700 years after the end of the Proto-Elamite system. The EDD Elamite texts deal with receipt, storage, and disbursement of materials such as metals and finished goods (Stolper 1984; Basello 2011; Basello and Giovinazzo 2018; Tavernier 2018), while at Tol-e Spid a single inscribed brick, a temple dedication of Shilhak-Inshushinak (1150–1120 BC), hints at a wider distribution of literacy across Fars (McCall 2013).

### 5.4 Summary of the Bronze Age CSWO Evidence: Diverse and Divergent Pathways

In summary of the mass of highly diverse CSWO evidence from Bronze Age Iran, several key points stand out. Firstly, Iranian societies’ engagement with the written word continued to be episodic and focused above all on Susa, doubtless the origin of writing in the Proto-Elamite style and then successively impacted by waves of literacy both imported from the west in the form of texts in Sumerian and Akkadian cuneiform and generated locally in the form of texts in Linear Elamite and Old Elamite scripts. With rare exceptions such as Chogha Gavaneh in the west and Tal-i Malyan in the east, the practice of writing in Bronze Age Iran was hugely focused on Khuzestan and the site of Susa above all, in particular as impacted by its near neighbours in Lower Mesopotamia to the west, including the Akkadian and Ur III imperial powers. Even during the Proto-Elamite phenomenon, when writing is attested at a range of sites more broadly across Iran, Susa stands out as by far the most significant source of inscribed material. But even at Susa, the trajectory of writing appears to have been tenuous and fragile, with significant breaks in the written tradition attested throughout the Bronze Age and beyond. Across Iran beyond Susa, the picture is even more fragmentary. We return to this point in our broader discussion at the end of the article.

Secondly, the practice of using seals in Iran across the Bronze Age shows some notable features, including a significant focus of activity at Susa and Chogha Mish in Khuzestan, with a major shift in emphasis from stamp seals to cylinder seals enabling a massive expansion of iconographic scale and scope. Through the Bronze Age we can also delineate a significant detachment of the practice of sealing from that of writing, with a steady decline in the proportions of inscribed tablets bearing seal impressions. This decline is clearly associated with a rise in the use of seals, cylinders above all, in non-writing administrative activities such as the widespread use of clay sealings for securing both immobile facilities such as storeroom doors and possibly windows as well as mobile containers such as pots, bags, sacks, and baskets.

A detachment of sealing from writing is especially notable in the urban communities of south-eastern Iran, at sites including Konar Sandal South, Tepe Yahya, and Shahr-i Sokhta, where rich cultures of seal use are attested by large assemblages of clay sealings completely separated from any system of writing. The lack of writing across this richly networked region in the Bronze Age, with the minor exception of a few non-administrative Linear Elamite texts, suggests a less centrally organised system of economic activity, with craft and trade organised at sub-state levels, arranged between families, households, and neighbourhoods without state-level oversight. The diversity of glyptic iconography attested at these sites might further support the notion of them as “intermediary markets” (Casanova 2019: 308), attracting individuals representative of peoples from across Southwest and Central Asia to engage in social and economic intercourse, bringing their highly distinctive seals with them along with their commodities and products for exchange. A systematic materials analysis programme of the clays of sealings from sites such as Konar Sandal South, Tepe Yahya, and Shahr-i Sokhta could greatly enhance our understanding of such issues. A specific detachment of the world of seals from any form of bureaucratic activity is also illustrated by the common practice of burying seals with the dead, as most persistently attested from the Chalcolithic to the Iron Age in the cemetery sites of Luristan, above all.

## 6 Iron Age Imperial Impacts, 1200–330 BC

The material evidence for counting, sealing, and writing across the centuries of Iran’s Iron Age, *c*. 1250–300 BC, continues as diverse, episodic, regionally distinctive, and inter-regionally impacted as in preceding ages of Iran’s tumultuous past. We here treat Iron Age Iran in two broad periods: Early-Middle Iron Age *c*. 1200–550 BC, and Later Iron Age *c*. 550–330 BC.

### 6.1 Early-Middle Iron Age, *c*. 1200–550 BC

The practice of depositing seals in elaborately furnished tombs, already attested in the Chalcolithic and Bronze Age of Luristan (above), continued in the Iron Age with 14 cylinder seals, one of which has a short inscription, and five stamp seals deposited amongst the wealth of objects buried with the dead in the Early Iron Age cemetery at Marlik in Gilan province (Negahban 1996; Abdi 2010). As with the earlier examples of this practice, there is no evidence to indicate use of these seals as anything other than cherished prestige objects rather than active components of an administrative system. It is possible that at least some of such seals were manufactured specifically for the purpose of burial with the dead (Mucheshi and Tala’i 2012). Similarly, 21 cylinder seals and three stamp seals occur in burials at Sialk in Cemetery B (Ghirshman 1939), and a single cylinder seal comes from Qara Tepe (Sagzabad) on the Qazvin plain west of Tehran (Dehpahlavan et al. 2019). At Hasanlu in level IVC a frit cylinder seal had been carefully placed in the mouth of an adult male burial, variously interpreted as an act of ‘sealing’ the mouth of the deceased, a magic amulet to support safe passage into the next world, or a gift or payment to the guardians of that world, or all of the above (Cifarelli 2013: 315).

**Figure 22:**
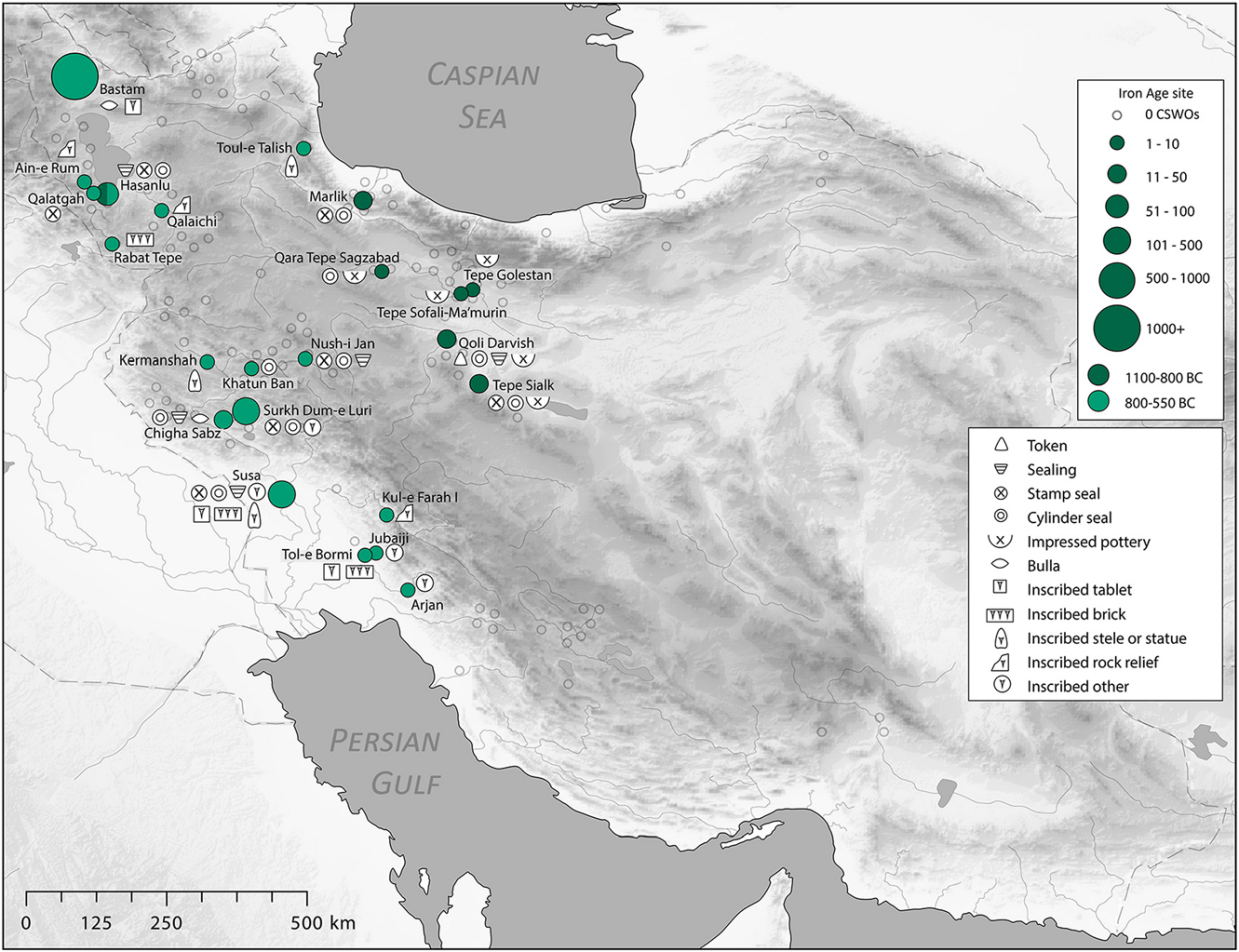
Map of CSWOs from Early-Middle Iron Age sites of Iran.

At Qoli Darvish near Qom two cylinder seals, at least six clay sealings with cylinder seal impressions, and eight spherical clay tokens were excavated from a room containing vessels for grain storage, associated with a shrine on a mudbrick platform of Early Iron Age date (Sarlak and Malekzadeh 2005; Sarlak 2011; Alibaigi and Khosravi 2014; Sarlak and Hessari 2018). Another characteristic of the Early Iron Age of the central plateau, attested at several sites, is the practice of rolling cylinder seals across the shoulders of large storage vessels prior to firing, with figurative designs featuring humans and animals engaged in activities such as ploughing (Alibaigi and Khosravi 2014). Such sherds are found, singly, at Qoli Darvish, Tepe Golestan, Qara Tepe (Sagzabad), Tepe Sofali-Ma’murin, and Tepe Sialk. The impression of cylinder seals on vessel shoulders is rare in Iran, and here may relate to the nature of vessel contents or as badges of ownership, or both. Overall, this modest but distinctive assemblage of administrative artefacts from a range of Early Iron Age sites of north-central Iran together may attest a concern to account for receipts, storage, and disbursements of agricultural produce, perhaps grain above all, by communities within a local, low-key administrative system (Figure 22) (Alibaigi and Khosravi 2014).

In north-western Iran, the major site of Hasanlu in its period IVB has yielded a wealth of material culture due to its violent and sudden destruction at *c*. 800 BC (Dyson and Muscarella 1989). Amongst the many finds from the destruction level, IVB, Danti and Cifarelli (2016, 362–363) list 52 cylinder seals, 19 stamp seals, and 31 clay sealings mainly with impressions of Local Style cylinder seals, at least some of which were applied to knobs securing boxes or store-room doors (Marcus 1989, 1996; Maras 2005). Two of the 31 sealings have stamp seal impressions. The majority of the seals and sealings from Hasanlu IVB come from Building BBII, a monumental columned-hall temple, and relate to the securing of temple storerooms or treasuries (Danti and Cifarelli 2016).

In Iron Age III, *c*. 800–550 BC, the increasing regional dominance of the kingdom of Urartu sees the appearance at sites of north-western Iran of writing in the form of rock-cut inscriptions, singly, at Qalatgah and Ain-e Rum (van Loon 1975; Salvini 2019). From the major fortress site of Bastam (Kleiss 1979, 1988), come five clay tablets in Urartian cuneiform script, all with cylinder seal impressions, and some 1416 clay bullae, formed over knots of string, the vast majority impressed with an inscribed cylinder seal of king Rusa II, as well as with other cylinder and stamp seals (Zimansky 1988; Dara and Shirzadeh 2017; Dara 2019, 2022). These sealings were associated with a mass of animal bones, arguably attesting regular episodes of accounting of meat stocks by officials operating on behalf of the king. Four cylinder seals and one stamp seal were also found at Bastam.

Less impacted by Urartu, Assyrian inscriptions on five glazed brick fragments at Rabat Tepe on the Lower Zab close to the Iraqi border (Kargar and Binandeh 2009; Reade and Finkel 2014; Ebrahimipour 2019), likely relate to a temple dedication. A single inscription in Aramaic occurs on a stele from Qalaichi, capital of the kingdom of Mannea (Lemaire 1988). At the cemetery site of Toul-e Talish in Gilan, a bronze bracelet with an Urartian inscription of Argishti was found in one grave, probably a late insertion (Vahdati 2007).

In Luristan, ongoing deposition of seals in sacred contexts is most richly attested in Iron Age levels of the sanctuary site at Surkh Dum-e Luri where some 125 cylinder seals, out of 175 found at the site, were deliberately incorporated into the walls and floors of the shrine (van Loon 1989c; Maras 2005). Many of the seals pre-date the sanctuary by some margin and reflect hoarding or curation of seals over generations. Thirty-three stamp seals and six inscribed beads or amulets were also found at this site (Brinkman 1989: 475–476). Eleven cylinder seals in provincial Neo-Assyrian style were found in graves at nearby Chigha Sabz (van Loon 1989d) plus a single cylinder seal from Khatun Ban to the north (van Loon 1989e: 413). As usual with the Luristan sites, no sealings were found at Surkh Dum-e Luri or Chigha Sabz, with the exception of a possible bulla and a stamp impressed sealing at the latter site (van Loon 1989f: 210–211).

In Iron Age Elam, seals are sporadically attested in tombs at Susa (de Miroschedji 1982), while 298 Neo-Elamite tablets from the Susa Acropole date to the seventh or early sixth century BC, as do two inscribed bricks, an inscribed door socket, and a stele fragment (Potts 2016: 291–295; Tavernier 2018: 420). The tablets relate to the movement of textiles, containers, weapons, and tools, with at least 355 different individuals named. One hundred and six Neo-Elamite cylinder seals, 37 stamp seals, and seven clay sealings with seal impressions from Susa show a blend of Elamite and Assyrian elements that strongly influence subsequent Achaemenid seal styles (Amiet 1972: 184–189; Garrison 2018). Carved reliefs at Kul-e Farah I include a lengthy Elamite inscription of a local prince who pays homage to the Elamite king Shutur-Nahhunte (Gorris and Wicks 2018; Álvarez-Mon 2020: 366–368). Neo-Elamite inscriptions dated to *c*. 600–570 BC are also attested on some of the spectacular finds from the tombs at Arjan (six inscribed objects) and Jubaji (four inscribed objects) on the Behbehan and Ram Hormuz plains respectively (Álvarez-Mon 2004; Shishegar 2008). One inscribed Neo-Elamite brick and one clay tablet fragment were also found on the surface at Tol-e Bormi on the Ram Hormuz (Wright and Carter 2003).

As yet we have no evidence for the indigenous use of writing within the Median kingdom – all our written sources originate from outside Media, from Assyria above all (Matthews and Fazeli Nashli 2022: 454–467). From within Media, finds of inscribed Assyrian stelae at a range of sites in west-central Iran, including Kermanshah and Najafabad, clearly mark major intrusion into Media by a succession of Assyrian kings (Alibaigi 2017; Alibaigi et al. 2020). At the Median site of Nush-i Jan, one cylinder seal, two stamp seals, and six clay sealings with impressions were found in the Western Temple (Curtis 1984: 24–25), which may relate to temple receipt and storage of offerings.

### 6.2 Later Iron Age, 550–330 BC, the Achaemenid Empire

**Figure 23:**
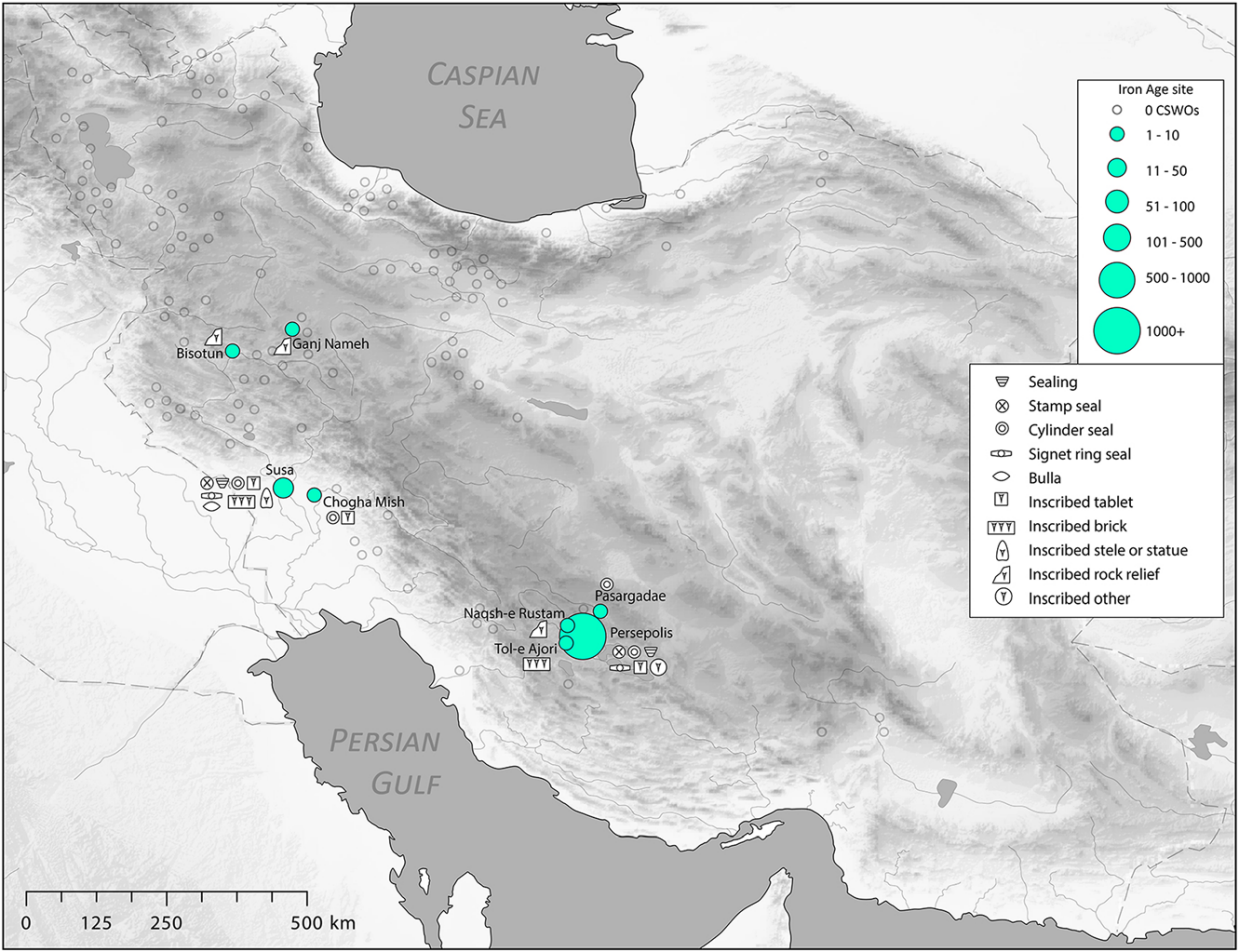
Map of CSWOs from Later Iron Age sites of Iran.

Through the course of the Achaemenid empire, *c*. 550–330 BC, writing and sealing became enmeshed in an imperial discourse at a range of socio-cultural levels, including the projection of power through monumental inscriptions (Figure 23). Table 1 summarises the types, media, and uses of writing within the Achaemenid empire. The table demonstrates clear connections between written language, inscribed medium, and the roles of writing, including the intended audiences. The empire expressed itself through its written records, including cuneiform clay tablets and monumental inscriptions (Stolper 2005; Kuhrt 2007; Henkelman 2013; Tavernier 2017), typically displayed in three, sometimes four, of the major imperial languages. Most famously, the inscriptions of Darius I at Bisotun near Kermanshah are in three languages (Garrison 2013): Old Persian, the Indo-European language of the ruling elite; Elamite, the ancient language of Khuzestan and Fars in southern Iran, used also in administrative texts; and the Babylonian dialect of Akkadian, an ancient Mesopotamian language of high learning. Garrison (2013, 575) has characterised the Bisotun relief and its associated inscriptions as “the only Achaemenid monument that unambiguously seeks to commemorate known and specific historical events”, and indeed we are not aware of another example from any of the other periods and regions featured within the remit of this study. At Ganj Nameh near Hamadan another trilingual inscription of Darius praises Ahura Mazda (Curtis 2005: 39), while a triplet of inscriptions adorns Gate R at Cyrus the Great’s city of Pasargadae but was probably added later by Darius (Boucharlat 2013: 510). Otherwise, no archives of texts and only one cylinder seal was found at Pasargadae (Stronach 1978: 178–179; Root 1999).

**Table 1:** Languages and writing systems of the Achaemenid Persian empire (information largely from Stolper 2005).

	Language group	Writing system	Writing media	Role and status	Exemplar
Old Persian	Iranian, Indo-European	Cuneiform; adapted script devised specifically for royal inscriptions; mixture of consonantal and syllabic signs	Royal inscriptions on stone, glazed bricks, cylinder seals; rare copies on clay tablets of royal inscriptions on stone	Used exclusively by Achaemenid kings “for the great king’s display of his presence and power” (Stolper 2005: 19–20)	Stone panel, Persepolis 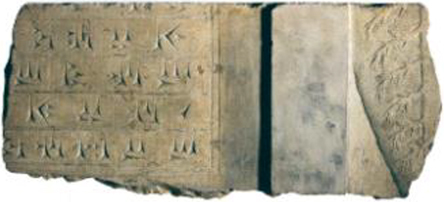
Elamite	No agreed known relatives, ancient or modern	Cuneiform; adapted script; ancient language of Elam	Clay tablets; occasional royal inscriptions on stone, glazed bricks	Administration; royal inscriptions; “language of practical literacy in Iran” (Stolper 2005: 20)	Wine ration text, Persepolis 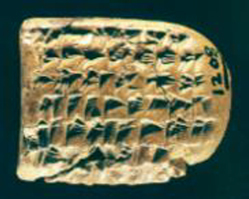
Babylonian dialect of Akkadian	Semitic	Cuneiform; Akkadian script	Clay tablets and cylinders; royal inscriptions on stone	“Language of learning that was ancient, manifold and still productive…it connoted domination over the world beyond Iran” (Stolper 2005: 21)	Cyrus cylinder, Babylon 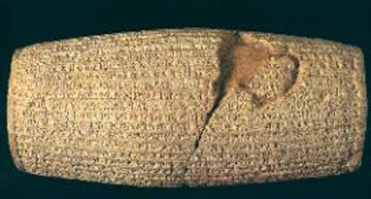
Aramaic	Northwest Semitic	Alphabetic/consonantal script with 22 characters	Inked on parchment, papyri, ostraca, occasionally on clay tablets; on portable objects such as seals, weights and coins; very rare copies on papyrus of royal inscriptions on stone (Bisotun)	Imperial *lingua franca*; used for legal and administrative matters; “under the Achaemenids its use spread to the remotest corners of the empire, from Egypt and Anatolia to central Asia” (Stolper 2005: 21)	Ostracon, Elephantine 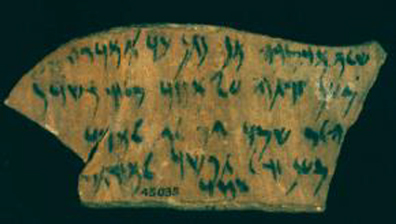
Egyptian	Semitic	Hieroglyphic	Royal inscriptions on stone stelae, stone statues (of Darius from Susa), stone vessels	Used rarely, always on stone objects with high royal status	Base of statue of Darius, Susa 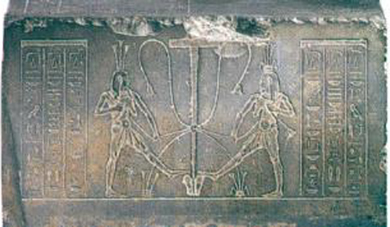

Beside these languages, all written in various forms of cuneiform script, the language of Aramaic was the single most important medium of communication across most of the empire. Aramaic is a northwest Semitic language of Syria and Upper Mesopotamia (Greenfield 1985; Joisten-Pruschke 2010), written alphabetically and most commonly on perishable materials such as leather and papyrus, occasionally on objects of clay or metal. Aramaic rapidly assumed its position within the empire as a *lingua franca* of legal and administrative activity, doubtless because of the relative ease of learning and reading its simple alphabetic script (Ostler 2006: 47–49). The rise to dominance of Aramaic probably accounts for the demise of cuneiform writing in Iran a decade or so prior to the collapse of the Achaemenid empire, as cuneiform texts are not attested for the reigns of Artaxerxes IV (338–336 BC) and Darius III (336–330 BC) (Black 2008). The destruction by fire of Persepolis during Alexander’s rampage enhanced the survivability of the clay cuneiform texts while at the same time potentially destroying thousands of Aramaic documents inscribed on highly combustible materials.

The richest source for Achaemenid writing and sealing, by far, is the major imperial capital site of Persepolis. According to Azzoni et al. (2017); see also Kuhrt (2007) and Jones and Stolper (2008), the scale and scope of the various types of administrative artefacts found at Persepolis are as follows. Firstly, the Persepolis Fortification Archive (PFA), excavated as a deposit from two rooms of the Fortification Walls at the north end of the Terrace, consists of 20,000–25,000 clay tablets and fragments representing up to 18,000 original documents, comprising those in Elamite cuneiform script (*c*. 10,000 documents), those in Aramaic script and language (*c*. 1000 documents), and those with only seal impressions and no texts (*c*. 6000 documents). There are also single texts in Greek, Old Persian, Akkadian, and Phrygian. This remarkable archive gives unique insight into the administration of the empire and its agricultural and craft production (Hallock 1969; Razmjou 2008; Henkelman 2013; Basello and Giovinazzo 2018: 489–492). Dating to 509–493 BC, the PFA texts deal solely with administrative matters such as receipt, storage, and disbursement of goods and animals to members of the royal family and the court, plus the organisation of temple staff, labourers, craft workers, and farmers.

Secondly, 198 tablets plus 548 small fragments, perhaps altogether representing *c*. 300 documents, were found in the so-called Treasury of Persepolis, dated to 492–457 BC, mainly dealing with payment of silver and food rations to skilled craft workers (Hallock 1960; Basello and Giovinazzo 2018: 492–494). Almost all these texts are in Elamite cuneiform. Also found with this archive were 199 clay sealings with impressions of cylinder, stamp, and ring seals. The sealings had been affixed to assorted boxes and bags secured with string. The majority of tablets from the Treasury archive had been formed around knots in string suggesting attachment to sealings or other tablets (Azzoni et al. 2017). Thirdly, a group of 52 clay sealings was found, along with three cylinder seals, in one of the fortification towers east of the Persepolis terrace, at least some of which may have secured folded or rolled documents of papyrus or parchment, which have not survived (Tadjvidi 1970). Additional evidence for writing at Persepolis comes in the form of two gold and two silver tablets with trilingual royal inscriptions of Darius I, found in an Apadana foundation deposit (Nimchuk 2010), and a trilingual monumental inscription of Xerxes. A monumental rock inscription of Darius I adorns the royal necropolis at Naqsh-e Rustam, close to Persepolis (Boucharlat 2013: 516–517).

Up to 4000 different seals are attested on the sealed tablets and sealings, “one of the largest bodies of imagery from anywhere in the ancient world” (Azzoni et al. 2017), and a vivid indication of the numbers of officials involved in sustaining the empire’s economic and craft sectors (Garrison 2017, 2018; Garrison and Root 2001; Root 2008). Of the Elamite tablets, about 85 % of them have seal impressions as well as text, with up to six different seals on a single document. All the Aramaic texts have seal impressions, with up to four different seals on a single tablet but more commonly with either one or two seals only. All the uninscribed tablets have seal impressions, mostly one or two but occasionally five different seals. Cylinder seals are more commonly attested than stamp seals across all types of document, ranging from 85 % on Elamite tablets, to 65 % on Aramaic documents, and 70 % on uninscribed tablets. Around 170 of the *c*. 4000 different seals, as attested by their impressions, have inscriptions, mainly in Elamite with a few in Aramaic, Akkadian, and Greek. Perhaps surprisingly, actual seals have been rarely found in excavations at Persepolis. In total 54 seals of all types – cylinder, stamp, signet ring – have been recovered from the site, a tiny proportion compared to the thousands of seals attested solely by their impressions (Root 1999: 181). Perhaps the seals were deposited in their owners’ graves in cemeteries which have yet to be discovered, and a proportion of the sealed documents may have originated from outside Persepolis.

At Tol-e Ajori, 3 km west of the Persepolis Terrace, a remarkable monumental gateway in Neo-Babylonian style includes short painted inscriptions on two glazed bricks (Basello 2013, 2017). Modest text and seal impression assemblages also come from Achaemenid contexts at Susa, including monumental inscriptions relating to the construction of Darius’s palace at Susa (Briant 2013), four inscribed clay tablets, two clay sealings, and eight sealed bullae (Amiet 1972: 37; Henkelman 2012: 954). One each of cylinder, stamp, scarab, and ring seals were also found at Susa (Amiet 1972: 37). The famous statue of Darius found at Susa (Yoyotte 2013) includes a unique inscription in four languages: Akkadian, Elamite, Old Persian, and Egyptian hieroglyphic.

Outside the great palatial Achaemenid sites of Persepolis and Susa the evidence for significant use of writing elsewhere in Iran, at least as materially manifest, is minimal, although assemblages of seals and sealings occur in quantity at sites as geographically scattered as Daskyleion in western Phrygia (Kaptan 2002) and Artashat in Armenia (Kuhrt 2001: 115). A few items of administrative activity in the form of an Elamite Achaemenid tablet, a cylinder seal, and a non-textual clay tablet with seal impression in the style of those from Persepolis were all found close to a circular probable granary at Chogha Mish on the Susiana plain (Delougaz, Kantor, and Alizadeh 1996: 10–12, 17–18). Overall, the administrative evidence from across the Achaemenid empire serves to stress the immense significance and reach of the site of Persepolis as the imperial capital of the Persian state.

### 6.3 Summary of the Iron Age CSWO Evidence: From Village to Empire

Through the Early-Mid Iron Age, *c*. 1200–550 BC, once more the site of Susa hosts the richest evidence, in terms of both quantity and range of CSWOs, including stamp seals, cylinder seals, sealings, plus a range of inscribed objects as detailed above. Much of the Susa evidence relates to engagement with the world of Mesopotamia to the west but also in terms of a strengthening sense of Elamite identity distinctive from the cultural traditions of Babylonia and Assyria (Álvarez-Mon 2020). Otherwise, bureaucratic activity across Iran can be characterised as modest and low-level through the Early-Mid Iron Age, with the only exception being the Urartian site of Bastam in the far north-west, an outlier from the state of Urartu with its core to the west. The scattered and scarce occurrence of practices such as rolling cylinder seals over pots before firing, attested at several sites across north-central Iran, and the occasional appearance of a few seals at these and other sites do not qualify as indicators of significant bureaucratic activity. Strikingly notable is the complete absence of material evidence for counting, sealing, and writing across the entire eastern half of Iran through the whole Iron Age, even where occupation has been identified. While the Iron Age of eastern Iran has been less studied than in western Iran, the same argument applies to earlier periods where we do find at least some traces of bureaucratic activity, as discussed in preceding sections. We can surmise that throughout the whole Iron Age the human societies of eastern Iran broadly eschewed any engagement with administrative practices involving the use of clay bureaucratic objects – they may have considered themselves fortunate to do so.

Another notable feature of the Iron Age evidence is the ongoing intensification in elite use of a range of media for expression of statements of royal power, manifest in the form of inscribed bricks, stelae, statues, and rock reliefs, with a focus on the far western reaches of Iran, north and south. This practice, emblematic of significant social inequality (Fochesato, Bogaard, and Bowles 2019), can be traced back to the mid-third millennium BC introduction at Susa from Mesopotamia by the Akkadian king Naram-Sin of the practice of using inscribed bricks to mark the construction of major buildings such as palaces and temples (McMahon 2020). All these features of the Early-Mid Iron Age evidence align to underpin the dramatic transition to the full imperial state of the Achaemenid domination of Iran, and well beyond, in the last centuries of the Iron Age. As we have seen, the highly focused distribution of Achaemenid CSWOs on the key site of Persepolis serves to highlight the unique significance of that site in all respects. This was a true information revolution of a type and intensity not previously experienced across the centuries of Iran’s past.

## 7 Revolutions and Transitions: Synthesis and Discussion

**Figure 24:**
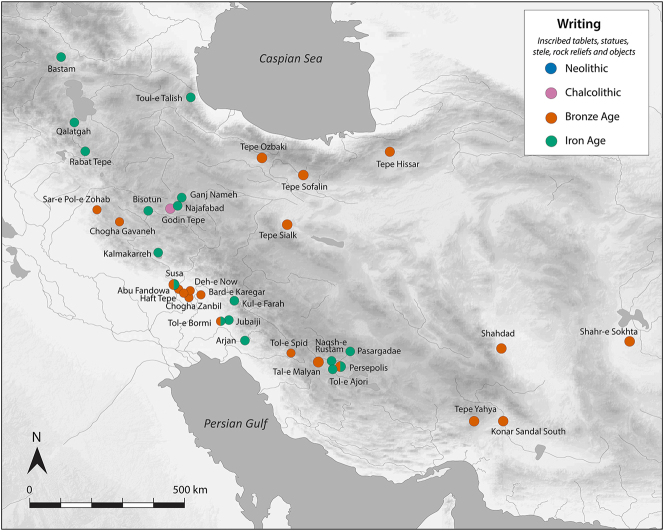
Writing in Iran through time.

**Figure 25:**
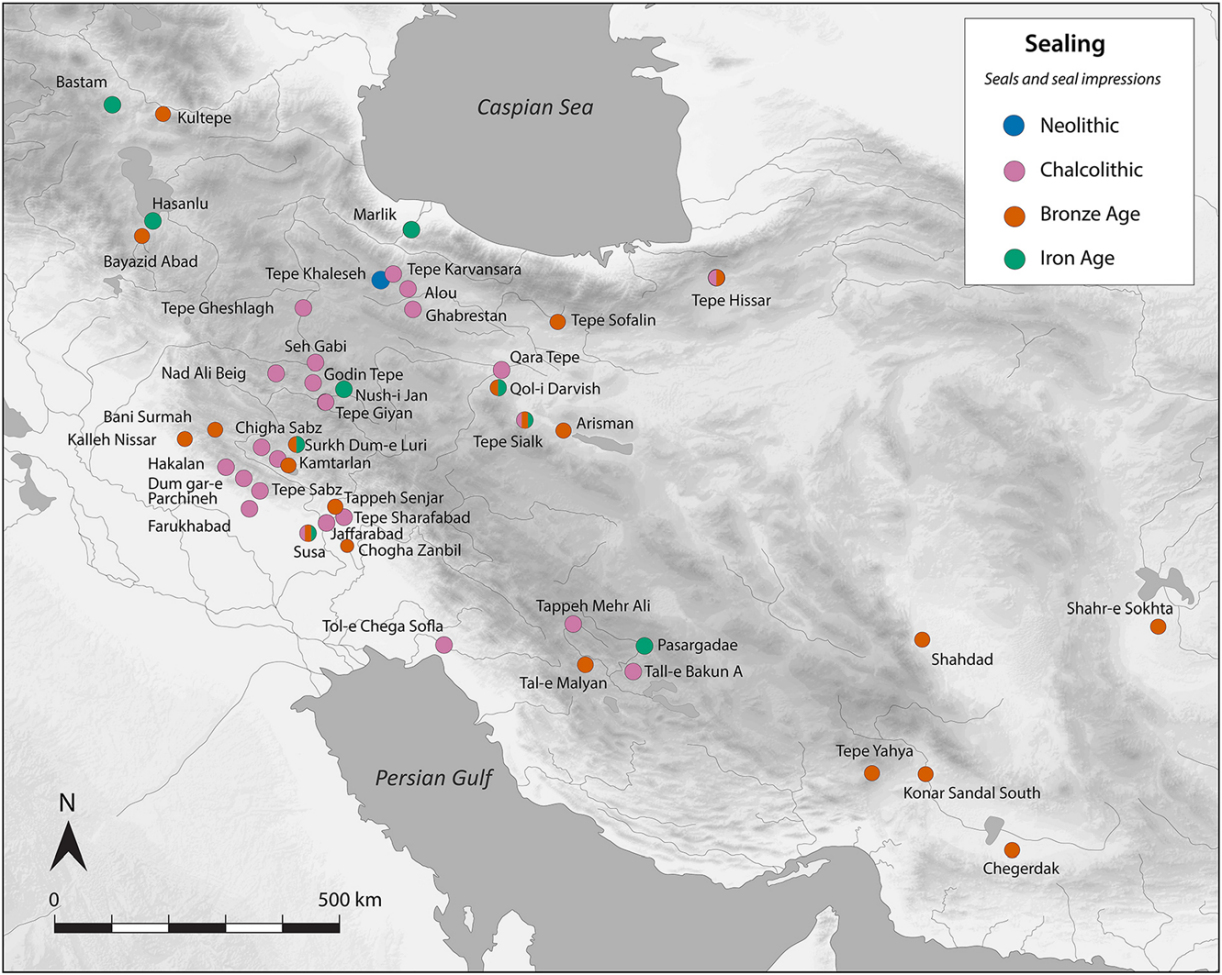
Sealing in Iran through time.

The CSWO evidence presented and discussed *in extenso* above enables us to articulate distinctive trends and patterns in the material traces of the practices of counting, sealing, and writing in Iran across a total time-depth of approximately 9000 years, as summarised in the following maps and diagrams (Figures 24 and 25; Table 2). While the period- and region-specific evidence has been thoroughly discussed in the relevant sections above, in this final section we pull together some of the deeper-time strands in an attempt to consider what factors might have shaped the distinctive trajectories of the Iranian CSWO evidence.

**Table 2:** Summary of the evidence for CSWO use from 10,000 to 330 BC.

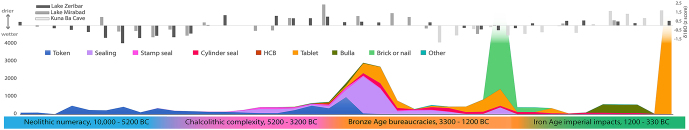

	Neolithic	Chalcolithic	Proto-Elamite/Early Bronze Age	Mid-later third millennium BC	Second millennium BC/Late Bronze Age	Early-Middle Iron Age, *c*. 1200–550 BC	Later Iron Age, 550–330 BC
Total CSWOs	3009	5094	3566	1277	10,652	2298	18,583
CSWO materials	Clay, stone, bone, bitumen.	Clay, stone, bone, gypsum, bitumen, frit, faience, chlorite, copper.	Clay, stone, bone, gypsum, bitumen, faience, chlorite, copper, shell, bronze.	Clay, stone, bone, bitumen, faience, copper, shell, bronze, silver, ivory.	Clay, stone, bone, bitumen, frit, faience, chlorite, bronze, gold, lapis lazuli.	Clay, stone, bone, bitumen, frit, faience, chlorite, bronze, gold, silver, shell, coral.	Clay, stone, gold, silver, bronze.
CSWO types	Clay tokens from *c*. 9800 BC. First sealings present at seventh millennium BC Sarab, sixth millennium BC Hajji Firuz and Khaleseh.	Tokens more complex. Impressed sealings from mid-fifth millennium BC, and Hollow Clay Balls from mid-fourth millennium BC at Susa and Chogha Mish. Cylinder seals and numerical tablets from *c*. 3500 BC.	Proto-Elamite tablets from *c*. 3200–2900 BC. Rapid increase in use of sealings; cylinder seals outnumber stamp seals. Decline in use of tokens.	Shift from tablets to seals and sealings in mid-third millennium BC. Pottery with seal impressions in southeastern Iran. Old Akkadian on stele and statues at Susa. Linear Elamite 2150–2000 BC.	Resurgence of tablets at Susa, almost entirely in Akkadian, *c*. 1950–1500 BC, followed by increase in inscription on bricks, nails, seals, beads, statues, and stele in Middle Elamite.	Cylinder seals in Luristan tombs. CSWOs and storage in north-central Iran. Urartu: bullae, rock-cut inscriptions and inscribed bracelet. Inscribed bricks. Seal-impressed pottery.	Clay tablets, monumental inscriptions, sealings impressed with cylinder, stamp, and ring seals, gold and silver tablets at Persepolis, inscribed statues, glazed bricks.
Bureaucratic practices and social change	Intensification in recording practices, storage, and management of agricultural produce from the early sixth millennium BC.	Growing administrative activity at independent and interconnected nodes. Concentrated at emerging state-level societies at Susa and Chogha Mish by Late Chalcolithic.	Agglomeration in regional centres until collapse of the proto-Elamite state *c*. 2900 BC. Administrative focus on control of agricultural production.	Sumerian influence until Akkadian conquest of Susa *c*. 2300 BC, followed by abrupt collapse. No evidence for state-level control in eastern Iran.	Ongoing Mesopotamian influence, focused in Khuzestan. Temple building programmes and high-level pronouncements.	Local, agricultural administration in EIA. Rising Urartian and Neo-Elamite, Neo-Assyrian, Achaemenid and Median empires exerting influence.	Intensified elite expressions of royal power. Achaemenid CSWOs massively concentrated at Persepolis.
Regional variation	Use of tokens common in the Central Zagros from Early Neolithic, extending along northeast and southeastern corridors in sixth millennium BC.	Strong emphasis on Khuzestan as key zone of complex recording systems. More modest indications of complexity in other regions, including Central Zagros, central plains, and northern highlands. Minimal engagement across eastern Iran.	Proto-Elamite phenomenon extends across Iran from Susa to Shahr-i Sokhta, with heavy focus on Susa as regards writing and sealing records. ETC/Kura Araxes Horizon in northwestern Iran with almost no evidence for administrative activity. Wide-ranging connections of western Iran richly attested in sealings at Chogha Maran. Early evidence for deposition of seals in graves in Luristan.	‘Middle Asian Interaction Sphere’ connects Iran with Mesopotamia and Indus Valley, appears to operate without writing but with major use of seals. Ongoing deposition of seals in Luristan tombs, but no evidence for their use as administrative artefacts.	Bureaucratic practices concentrated in Elam. Outpost of Mesopotamian impact at Chogha Gaveneh in Central Zagros. Collapse in settlement in Fars with writing absent until *c*. 1100 BC.	Localised systems in various languages, with focus on Susa once more. Seal deposition continues in Luristan tombs. Seal impressions on pottery vessels in north-central Iran. Absence of CSWO evidence from all of eastern Iran.	Heavy focus on Persepolis. Ongoing absence of CSWO evidence from all of eastern Iran plus northern Iran.
Environmental conditions	Increasingly warm and wet conditions of the Early Holocene, with rapid climate change events at 9.2 and 8.2 ka BP.	Expansion and intensification of CSWO systems, at least in Khuzestan, coincides with episode of drier climate spanning late fourth-early third millennium BC.	Increased aridity from *c*. 3200 BC contemporary with abandonment of rural settlements and increased agricultural bureaucracy.	Collapse of the Akkadian empire coincides with the 4.2 ka BP event and increased aridity. Societies of eastern Iran also collapse at approximately the same time.	Collapse at end of Late Bronze Age, arguably associated with climatic downturn attested elsewhere across Southwest Asia.	Spread of imperial powers coincides with propitious climate (wetter).	Intensification of CSWO use in Achaemenid empire coincides with climate downturn (drier).

The evidence indicates that the societies of ancient Iran engaged with the practices of sealing and writing more hesitantly and episodically than their neighbours in Mesopotamia to the west, where the tradition of cuneiform writing on clay tablets and the bureaucratic use of seals persisted almost constantly as integral features of urban society following the invention of writing there at *c*. 3300 BC and the invention of the cylinder seal a few centuries earlier. As expressed by Vidale (2018, 277), “Writing technologies appeared on the Iranian Plateau as a rare, discontinuous variable; their evolutionary trajectory is unknown, and the two main systems presently under study (so-called Proto-Elamite and Linear Elamite) are still far from being deciphered.” Similarly, Dahl’s (2018, 393–394) comment that “Writing is invented more times in Iran than in any other place in the world”, taken as evidence for “an extraordinary ingenuity rarely matched in other ancient civilizations”, emphasises the episodic nature of writing traditions in Iran.

Why should the deep-time pattern of CSWO activity in Iran appear so different from that of Mesopotamia, immediately to the west, at least in the latter millennia of the long period featured in this study? One answer may lie in the essential fragility of the agricultural systems that underpinned Iranian societies from the Neolithic onwards. Diachronic studies of human-environment interactions in Iran, including the impacts of climate change (Kehl 2009; Sharifi et al. 2015; Fallah et al. 2017; Gurjazkaite et al. 2018; Vaezi et al. 2019; Matthews and Fazeli Nashli 2022; Kehl, Rafiei-Alavi, and Lahijani 2023; Rafiei-Alavi, Kehl, and Lahijani 2023), agree in highlighting the potential for sometimes minor fluctuations in levels of precipitation and temperature to impact upon the agriculturally based societies of early Iran. Such impacts could be especially catastrophic in those parts of Iran where the environmental and climatic parameters for productive agriculture were rather restricted, including much of the central and eastern regions of the country, where episodes of settlement collapse are frequently attested.

We have repeatedly seen that the key role of counting, sealing and, especially, writing in early Iran was to administer and control the agricultural economy, its fields, crops, animals, and human labour, often on behalf of central organising institutions (Giraudeau 2017). In those frequent episodes when agriculture collapsed as a result of climate change, human communities either moved elsewhere or they changed their basic food-producing lifeways from sedentary farming to more mobile forms of animal management, in which case sealing and writing lost all relevance and were abandoned. As Lamberg-Karlovsky (1999, 185) asserts: “Cultures lacking a centralised bureaucracy had little use for an invention dedicated to administering a bureaucratic hierarchy. In rejecting the use of the inscribed tablet, they simply rejected an artifact that had neither function nor utility in their own society.”

Only with the adoption of Akkadian cuneiform writing in the mid-later third millennium BC, at Susa, Malyan, and Chogha Gavaneh, at least, and much later still with the development of the cosmopolitan Achaemenid Persian empire and its use of multiple scripts and languages, is bureaucratic activity in Iran associated with concerns beyond the essentially agricultural. By contrast, in Mesopotamia with its ecologically less fragile cities and states, textual activity had developed to include multiple areas of life including legal concerns, business records, letters, royal inscriptions, treaties, and literary texts (Postgate 1992: 66, fig. 3.13).

At the same time, with some exceptions, in early Iran there is a persistent elite connection of writing, especially, and often of sealing too. But elites were only as stable as the kingdoms, states, and empires under their control: “in the earliest states, writing developed first as a technique of statecraft and was therefore as fragile and evanescent an achievement as the state itself” (Scott 2017: 148). As complex hierarchical societies came and went, writing came and went with them as they departed. With some exceptions in Akkadian writing from Susa, the practice of writing was not embedded in non-elite strata of early Iranian society, for example amongst merchant or property-owning classes, in contrast to contemporary Assyria and Babylonia, and was therefore easy to cast off as an undesirable characteristic of controlling elites, when times got tough. As phrased by Scott (2017, 139) “the first act of many peasant rebellions has been to burn down the local records office where these documents are housed.” A similar sentiment underpins Lamberg-Karlovsky’s (2001, 221) proposal that certain societies, including those adjacent to the newly literate cities and states of Lower Mesopotamia in the third millennium BC opted not to engage with writing, thus avoiding “the cage of the state for another half-millennium.”

Even within expansive imperial contexts, such as the Achaemenid empire, writing led a delicate and flexible existence. Black’s (2008) study of the demise of cuneiform writing in Elam stresses how evidence for the cuneiform tradition in Achaemenid Iran, employed to write Elamite, Akkadian, and Old Persian, came a halt up to 10 years *prior to* the fall of the empire, with no cuneiform texts known from the reigns of Artaxerxes IV (338–336 BC) and Darius III (336–330 BC), a demise probably connected with the rise to dominance over the preceding decades of Aramaic as a spoken and written language within and beyond the Achaemenid empire. A key issue here is the differential survival of the inscribed evidence: while destruction by fire is generally good for clay tablets, baking them to durable hardness and long-term survivability, the impact on Aramaic documents is devastating: “it is possible that thousands of fifth to fourth century BC Aramaic parchment documents went up in smoke when the Persepolis archives were destroyed by fire” (Black 2008: 59).

Alongside the episodic trajectory of the practice of writing within Iranian complex societies, the trajectory through time of seal use in Iran, often closely associated with writing since their first co-occurrence at the invention of writing, is also of note. Supporting earlier overviews of seal use in Eurasia (Laurito 2000; Rahmstorf 2011), the Iranian evidence reveals a geographical expansion of seal use through time, a proportional shift through time from stamp to cylinder seal use, and a major phase of activity associated with the transregionally engaged craft production and trading centres of the third millennium BC, as detailed above.

In addition to the persistent elite and institutional connections, there is significant evidence from Iran attesting the use of CSWOs within private, domestic households, even after the rise to dominance of major institutions at Susa and Chogha Mish, suggesting that the seals and sealing use evidence from Iran cannot readily be characterised as either Mesopotamian or Indus style in the degree of its association with elite socio-political elements, as broadly schematised by Green (2020). The Iranian evidence needs to be evaluated on its own terms. In earlier periods, from the origins of CSWOs in the Neolithic to the start of the Late Chalcolithic period in the mid-fourth millennium BC, tokens, seals, and sealings operated within what Lamberg-Karlovsky (1999, 168), in his overview of seals and sealing practices across Mesopotamia from the sixth millennium BC onwards, has characterised as “basically egalitarian communities” where the household was the focus of production and consumption. In Mesopotamia this situation changed dramatically with the invention of writing at Uruk and its subsequent adoption across much of Mesopotamia and beyond. Writing at Uruk in its early centuries of use, *c*. 3300–3000 BC, was focused heavily on institutional control over the receipt, storage, and distribution of materials, products, and commodities, including people, land, and animals, together indicative of an increasingly centralised and hierarchical society epitomised by the massive public architectural contexts of Late Chalcolithic Uruk within which much of the CSWO evidence was recovered (Englund 1998; Boehmer 1999; Liverani 2006). In south-western Iran, however, while there is a wealth of evidence to suggest close contacts between Uruk and Susa in the Late Chalcolithic (Algaze 2001), the contexts at Susa of the immediate precursors of writing – complex tokens, cylinder and stamp seals, sealings, hollow clay balls, and numerical tablets – reveal on ongoing focus of CSWO activity within private, domestic households, in marked contrast to the excavated evidence from Uruk (Englund 1998).

In this article, we aimed to explore the interpretive potential of the Iranian bureaucratic evidence for investigating the interactions between social, cultural, political, economic, and environmental factors in structuring societal formations across millennia of the Iranian past, *c*. 10,000–300 BC. Furthermore, a future aim would be to situate the deep-time Iranian bureaucratic evidence within the broader context of ambitious, deep-time, big-data projects that are currently shedding new light on issues germane to ancient Iran and the wider geographical situation of the Middle East and well beyond. Such diachronic studies include those of the archaeological distribution of cuneiform inscriptions (Rattenborg et al. 2021, 2023), demography and palaeoclimate (Palmisano et al. 2021; Kehl, Rafiei-Alavi, and Lahijani 2023; Rafiei-Alavi, Kehl, and Lahijani 2023), emergent inequality and imperial development (Squitieri and Altaweel 2022), human societal development (Carmel 2023), animal exploitation, landscapes, and climate (Gaastra et al. 2021), and cereal production, climate, and social formation (Ghahremaninejad, Hoseini, and Jalali 2021).

To aid in future synthetic and analytical studies of the CSWO evidence from Iran, and from elsewhere across Southwest Asia, we conclude with the following recommendations (see also Basello and Giovinazzo 2018: 497–498):

– Further development of theoretical frameworks for the analysis of early bureaucratic systems, for example through the adaptation and application of current anthropological approaches to contemporary and historical bureaucratic systems (e.g. Bierschenk 2019; Bierschenk and de Sardan 2019).
– Consideration of lessons learnt from study of materialities of communication and administration from other places and periods, including the potential for the integration of socio-linguistics and material culture, as practiced for example in Mullen’s studies of Latin documents (Mullen 2023) or Boyes’ work on writing and society at Late Bronze Age Ugarit (Boyes, Steele, and Astoreca 2021).
– Full publication of CSWO assemblages, at least as freely available online resources, with high-resolution images as well as in Open Access pdf/hard copy volumes.
– Consistent and full cataloguing of CSWO assemblages in a standardised manner, precise details to be agreed upon through future work, including, e.g., qualitative and quantitative data on reverse sides of clay sealings to assist with determining their function (such as store-room door sealing vs container sealing).
– Integrated publication of related components of CSWO administrative systems, e.g. seal impressions on tablets to be published alongside tablets, seal impression images alongside those of clay sealings as objects in their own right (see Jans and Bretschneider 2012 for exemplar), to enable correlation of aspects of obverse and reverse faces of sealings in order to identify specific roles for individual offices/officers within bureaucratic systems.
– Where resources allow, archaeometric analysis of the physical media of the CSWO evidence, e.g. by chemical characterisation and provenance studies of the clays used in the production of sealings and tablets.
– Integration of all the above into coherent narratives within appropriate anthropological frameworks.

## Supplementary Material

Supplementary Material
